# *Conocarpus lancifolius* (Combretaceae): Pharmacological Effects, LC-ESI-MS/MS Profiling and In Silico Attributes

**DOI:** 10.3390/metabo13070794

**Published:** 2023-06-27

**Authors:** Muhammad Khurm, Yuting Guo, Qingqing Wu, Xinxin Zhang, Muhammad Umer Ghori, Muhammad Fawad Rasool, Imran Imran, Fatima Saqib, Muqeet Wahid, Zengjun Guo

**Affiliations:** 1 School of Pharmacy, Xi’an Jiaotong University, Xi’an 710061, China; khurmmuhammad411@stu.xjtu.edu.cn (M.K.); gggyutingiii@stu.xjtu.edu.cn (Y.G.); wuqingq@stu.xjtu.edu.cn (Q.W.); 2 Department of Bioinformatics and Biotechnology, Government College University, Faisalabad 38000, Pakistan; umerghori90@gmail.com; 3 Department of Pharmacy Practice, Faculty of Pharmacy, Bahauddin Zakariya University, Multan 60800, Pakistan; fawadrasool@bzu.edu.pk; 4 Department of Pharmacology, Faculty of Pharmacy, Bahauddin Zakariya University, Multan 60800, Pakistan; imran.ch@bzu.edu.pk (I.I.); fatima.saqib@bzu.edu.pk (F.S.); muqeet.soomro@bzu.edu.pk (M.W.)

**Keywords:** *Conocarpus lancifolius*, antioxidant, cardioprotection, anxiolytic, antidepressant, memory enhancement, secondary metabolites, molecular docking, network analysis

## Abstract

In folklore medicine, *Conocarpus lancifolius* is used to treat various illnesses. The main objective of this study was a comprehensive investigation of *Conocarpus lancifolius* leaf aqueous extract (CLAE) for its antioxidant, cardioprotective, anxiolytic, antidepressant and memory-enhancing capabilities by using different in vitro, in vivo and in silico models. The in vitro experimentation revealed that CLAE consumed an ample amount of total phenolics (67.70 ± 0.15 µg GAE/mg) and flavonoids (47.54 ± 0.45 µg QE/mg) with stronger antiradical effects through DPPH (IC_50_ = 16.66 ± 0.42 µg/mL), TAC (77.33 ± 0.41 µg AAE/mg) and TRP (79.11 ± 0.67 µg GAE/mg) assays. The extract also displayed suitable acetylcholinesterase (AChE) inhibitory (IC_50_ = 110.13 ± 1.71 µg/mL) activity through a modified Ellman’s method. The toxicology examination presented no mortality or any signs of clinical toxicity in both single-dose and repeated-dose tests. In line with the cardioprotective study, the pretreatment of CLAE was found to be effective in relieving the isoproterenol (ISO)-induced myocardial injury in rats by normalizing the heart weight index, serum cardiac biomarkers, lipid profile and various histopathological variations. In the noise-stress-induced model for behavior attributes, the results demonstrated that CLAE has the tendency to increase the time spent in the central zone and elevated open arms in the open field and elevated plus maze tests (examined for anxiety assessment), reduced periods of immobility in the forced swimming test (for depression) and improved recognition and working memory in the novel object recognition and Morris water maze tests, respectively. Moreover, the LC-ESI-MS/MS profiling predicted 53 phytocompounds in CLAE. The drug-likeness and ADMET analysis exhibited that the majority of the identified compounds have reasonable physicochemical and pharmacokinetic profiles. The co-expression of molecular docking and network analysis indicated that top-ranked CLAE phytoconstituents act efficiently against the key proteins and target multiple signaling pathways to exert its cardiovascular-protectant, anxiolytic, antidepressant and memory-enhancing activity. Hence, this artifact illustrates that the observed biological properties of CLAE elucidate its significance as a sustainable source of bioactive phytochemicals, which appears to be advantageous for pursuing further studies for the development of new therapeutic agents of desired interest.

## 1. Introduction

Globally, cardiovascular diseases (CVDs) continue to be the leading cause of death and are a significant contributor to health loss and excessive health system costs. An estimated 17 million deaths globally are attributed to CVDs each year. The majority of CVD fatalities occur in low- and middle-income nations [1]. The root cause of CVDs is the abnormalities of heart and blood vessels, which may include congenital heart disease, myocardial infarction (MI), stroke (cerebrovascular disease), hypertension (high blood pressure), peripheral artery disease (PAD) and rheumatic heart disease (RHD) [2]. Likewise, noise or sound pollution has become the most widespread environmental and occupational threat to human health. When the noise level exceeds 100 dB, it becomes a stressor source [3]. Uncontrolled exposure to environmental noise involves the etiopathogenesis of severe physiological effects including anxiety, metabolic changes in neuronal functions, depression, learning and memory deficits, annoyance and other cognitive failures [4,5]. According to the recent reports of the World Health Organization (WHO), neurological disorders affect around 450 million people worldwide. These mental conditions have a ‘ripple effect’ on patients and their family members [6].

In various cardiovascular and mental disorders, a well-recognized role of reactive oxygen species (ROS)-related damage has been already established [7,8,9]. The production of ROS or antioxidant dysfunction can initiate several mechanisms such as the activation of metalloproteinase, alterations in vasomotor functions, lipid peroxidation, smooth muscle cell migration and cell proliferation. These pathways commonly lead to several CVDs [7]. Similarly, disruptions in ROS balance in neuronal cells, variations in neuroinflammatory processes along with neurodegenerative abnormalities within the hippocampus and prefrontal cortex and apoptotic cell death seem to be the possible molecular mechanisms involved in anxiety, depression and impaired memory and learning triggered by noise stress (NS) [4,8,9]. Several scientific studies have shown that chemo-deterrence using medicinal herbs can be used to counteract and neutralize various ROS-induced cellular damages because these herbs contain a variety of natural antioxidants including polyphenols, especially flavonoids, alkaloids, glycosides, tannins, stilbenes, etc., which provide a defense mechanism against multiple life-threatening disorders, such as atherosclerosis, cancer, MI, Parkinson’s disease and Alzheimer’s disease (AD) [10,11]. Therefore, in today’s era, the therapeutic importance of plant medicines fosters the need to hunt for new medicinal agents capable of slowing the progression of numerous CVDs [12,13] and neurological disorders [14,15,16,17,18,19,20]. 

*Conocarpus lancifolius* Engl. (*C. lancifolius*) is one of the two species of genus *Conocarpus* in the family Combretaceae. This flowering tree is widely distributed among the coastal areas of Africa and the Arabian Peninsula and also cultivated in several constituencies of Pakistan [21,22]. Traditionally, *C. lancifolius* is used for the treatment of catarrh, fever, diabetes, diarrhea and skin ulcers [23,24]. Different studies have examined the antibacterial [21,25], antidiabetic [23,26], antioxidant [11,22,23], antiurease, phytotoxic [22], cytotoxic, anti-inflammatory and peroxisome-proliferator-activated receptor (PPAR) agonistic [27] effects of this plant. Al-Musayeib et al. [28] evaluated the antiplasmodial, antileishmanial and antitrypanosomal potential of *C. lancifolius*. Its acetylcholinesterase (AChE) and lipoxygenase (LOX) inhibitory attributes were also investigated by Raza et al. [29]. In line with the chemical composition, the high-performance liquid chromatography (HPLC) quantification of aerial parts and roots of *C. lancifolius* displayed the presence of diverse phenolics and flavonoids such as caffeic acid, chlorogenic acid, ferulic acid, gallic acid and quercetin [22]. The ultra-high-performance liquid chromatography–quadrupole time-of-flight mass spectroscopy (UHPLC-Q-TOF-MS/MS) and proton nuclear magnetic resonance (^1^H-NMR) analysis identified gallic acid, galloyl-HHDP-glucoside isomers, ellagic acid, epicatechin, scopoletin, isorhamnetin, quercetin-3-*O*-α-rhamnoside, quercetin-3-*O*-β-glucoside, skimmianine, corilagin, cornoside, terflavin B, phyllanthin and hypophyllanthin as major functional phytochemicals in the *C. lancifolius* leaf ethanolic extract [23,29]. Another phytochemical study of *C. lancifolius*’s aerial parts revealed the presence of two ellagic acid derivatives, namely 2,3,8-tri-*O*-methylellagic acid and 3-*O*-methylellagic acid 4-*O*-β-D-glucopyranoside, with prominent antioxidant and anticancer properties [30]. In a prior report, Al-Taweel et al. [27] isolated a new trimethoxy ellagic acid derivative from the fruits of this plant. This compound showed strong PPAR activity. Taken together, very limited work has been carried out on the pharmacological potential of different extracts and secondary metabolites (SMs) of *C. lancifolius* by targeting multiple animal models with different paradigms. 

Based on previous research, a range of medicinal plants with noteworthy antioxidative capabilities have been evaluated for their role in the medication therapy of several CVDs such as angina pectoris, cerebral insufficiency and MI [2,12,13] and NS-induced anxiety, depression and cognitive dysfunction [3,14,15,16,17,18,19,20]. However, despite the prodigious biological profile of *C. lancifolius*, no scientific research has been conducted to determine its preventive effects on isoproterenol (ISO)-induced chronic MI and NS-induced anxiety, depression-like behavior and memory impairment. Therefore, in this frame, we highlighted the in vitro antioxidant potential of *C. lancifolius* aqueous leaf extract (CLAE) followed by its cardioprotective, anxiolytic, antidepressant and memory-enhancing role in different animal models to further authenticate its pharmacological capacity. Afterward, the liquid chromatography–electrospray ionization–tandem mass spectrometry (LC-ESI-MS/MS) screening was performed to explore various phytoconstituents present in CLAE. Furthermore, a computational approach involving absorption, distribution, metabolism, excretion and toxicity (ADMET) parameters, molecular docking and network analysis was incorporated to find out its pharmacokinetic profile from a drug-likeness perspective and to hypothesize the multi-target underlying mechanisms of observed biological activities. 

## 2. Materials and Methods

### 2.1. Plant Material and Sample Preparation 

The *C. lancifolius* leaves were collected from the surroundings of district Okara (30°48′ N, 73°27′ E), Pakistan, in August 2020 and identified by Dr. Muhammad Zafarullah, Institute of Pure and Applied Biology, Bahauddin Zakariya University (B.Z.U), Multan, Pakistan. Voucher no. “Kaw-2734524” was assigned to the specimen and preserved in the university herbarium. Leaves were instantly cleaned with tap water and shade-dried for 20–25 days at 37 °C. These dried leaves were crushed into a coarse powder using a mechanical herb grinder. The aqueous extraction (50–60 °C; 1:5, *w*/*v*) of ground leaves (400 g) was performed using a soxhlet apparatus for 72 h and then filtered with the help of Whatman filter paper (no. 1). The extract was made concentrated by using a rotary evaporator (Buchi R-200 system) under reduced pressure at 40–45 °C. The concentrated extract was stored at 4 °C for further evaluation. The powdered leaf extraction yielded 48.3 g of CLAE, approximately 12.07% of the total dry weight. 

### 2.2. Chemicals and Reagents

All the analytical-grade chemicals, reagents and standards in this study were obtained from Sigma-Aldrich (Burlington, MA, USA) through Merck Life Science Ltd., Shanghai, China. 

### 2.3. Estimation of Total Phenolic Content (TPC)

A slightly modified Folin–Ciocalteu method was used for the determination of TPC [31,32]. Firstly, 20 μL of the sample solution was mixed with the Folin–Ciocalteu reagent (90 μL, 10% *w*/*v*) in a 96-well plate. After incubation for 5 min, 90 μL of sodium carbonate (6% *w*/*v*) was added to the reaction mixture. Gallic acid and pure DMSO solutions were used as positive and negative control, respectively. At the end, the absorbance of the final reaction mixture in the 96-well plate was measured at 630 nm by placing it in the microplate reader (Tecan Infinite F50, Mannedorf, Switzerland). The methanolic solutions of standard gallic acid (5, 10, 15, 20 and 25 µg/mL) were used to acquire the calibration curve for the calculation of TPC and expressed as microgram gallic acid equivalents per milligram (μg GAE/mg) of the plant extract. 

### 2.4. Estimation of Total Flavonoid Content (TFC)

The colorimetric method of Chang et al. [33] was used for the estimation of TFC with some alterations. Briefly, 20 µL of the extract solution, 10 µL of aluminum chloride (10% *w*/*v*), 10 µL of potassium acetate (1M) and 160 µL of distilled water were introduced into the wells of a 96-well plate. The resulting mixture was mixed carefully and incubated at 37 °C for 30 min. Quercetin was used as a positive and pure DMSO as a negative control. Finally, absorbance was measured at 405 nm by means of a microplate reader. The same operating conditions were adopted to prepare the calibration curve of quercetin with the final concentrations of 5, 10, 15, 20 and 25 µg/mL. TFC values were stated in terms of microgram quercetin equivalents per milligram (μg QE/mg) of the crude plant extract. 

### 2.5. Antioxidant Assays

#### 2.5.1. DPPH Radical Scavenging Assay

The antiradical capacity of CLAE to scavenge the 2,2-Diphenyl-1-picrylhydrazyl (DPPH) radical was investigated according to the procedure of Phull et al. [34] with little modifications. In this method, 20 µL of CLAE from several dilutions (31.25, 62.5, 125 and 250 µg/mL) was added with 3.2 mg/100 mL of the DPPH methanolic solution (180 µL) into the 96-well plates. After shaking gently, the reaction mixture was incubated for 60 min at room temperature, and the absorbance of this mixture was recorded at 517 nm by using a microplate reader. Ascorbic acid (AA) was used as a reference antioxidant. Lastly, the screening results were expressed in terms of percentage scavenging (% scavenging), which was calculated from the given equation.

PS = 1 − (OD of tested sample/OD of control) × 100

where “PS” represents % scavenging and “OD” shows the optical density.

#### 2.5.2. Total Antioxidant Capacity (TAC)

The phosphomolybdenum assay was used to examine the TAC of CLAE with few adjustments in the method of Phull et al. [34]. In the 96-well plate, 100 µL of the sample solution was thoroughly mixed with 90 µL of the reagent (28 mM sodium phosphate, 4 mM ammonium molybdate and 0.6 M sulphuric acid) and incubated for 90 min at 95 °C. After incubation, the reaction mixture was cooled, and its absorbance was checked at 695 nm on the microplate reader. AA was served as a positive control, and the TAC was stated as microgram ascorbic acid equivalents per milligram (μg AAE/mg) of the dry extract. 

#### 2.5.3. Total Reducing Power Assay

The potassium ferricyanide colorimetric assay was used to estimate the total reducing power (TRP) of the tested extract in accordance with the previously described method [35,36]. In this procedure, the preliminary reaction mixture was prepared by the addition of 100 µL of CLAE solution with trichloroacetic acid (250 µL, 10% *w*/*v*) and phosphate buffer (250 µL, 0.2 M). After this, the centrifugation of the mixture was performed at 3000 rpm for 10 min. After centrifugation, 150 µL of the supernatant of the above reaction mixture was mixed with 50 µL of ferric chloride (0.1% *w*/*v*) in the 96-well plate. GA was used as a standard drug. Finally, the microplate reader was used to observe the absorbance of the resulting mixture at 630 nm, and the TRP capacity of CLAE was determined in terms of microgram gallic acid equivalents (μg GAE/mg of dry weight extract). 

### 2.6. Acetylcholinesterase (AChE) Inhibitory Activity

The Ellman’s method-based 96-well microplate procedure of Mathew and Subramanian [37] was used with minor modifications to study the in vitro AChE inhibitory potential of CLAE. In this protocol, 50 µL of buffer (Tris-HCl 50 mM, pH 8.0), 100 µL of 5,5-dithiobis-2-nitrobenzoic acid (DTNB, 3 mM), 25 µL of extract solution with different concentrations (12.5, 25, 50 and 100 µg/mL) prepared in a buffer containing not more than 10% methanol and 25 µL of 0.26 U/mL of AChE (dissolved in sodium phosphate buffer, pH 8.0) were dispensed in the 96-well plate. This assay mixture was mixed rigorously and incubated at 30 °C for 15 min. After this, absorbance was recorded in a microplate reader at 412 nm and considered a basal reading. Then, 20 µL of acetylthiocholine iodide (ATCI, 15 mM) was added as a substrate into the above mixture, and the AChE hydrolysis was monitored by measuring the absorbance changes for 20 min with a 5 min interval. Galantamine was used as a positive control. The outcomes of percentage enzymatic inhibition (% inhibition) were interpreted from the following equation.

% Inhibition = (E S)/E × 100

where “E” is the enzymatic activity without the sample solution and “S” is the activity of the enzyme with a test compound. 

### 2.7. Animals and Their Housing Conditions

In the present study, 60 healthy male Sprague Dawley (SD) rats and 25 BALB/c mice were provided by the animal house source of the Faculty of Pharmacy, B.Z.U Multan, Pakistan. The animals were housed separately in polycarbonate cages with free access to standard mouse chow cubes and ad libitum drinking water. The standard hygienic conditions such as temperature (25 ± 2 °C) and relative humidity (50 ± 3%) were maintained in the locality of animals followed by a light–dark cycle of 12 h (lights on between 7:00 a.m. to 7:00 p.m.). The animal study protocols were developed in accordance with the recommendations of the Institute of Laboratory Animal Resources (ILAR), National Research Council [38], and endorsed by the ethical committee of the Faculty of Pharmacy, B.Z.U Multan, Pakistan (ref. no. EC/PhD/4117999028/2017). 

### 2.8. Acute Toxicity Study

Ten randomly selected male BALB/c mice (weighing 25–35 g) were used to investigate the acute toxicity of CLAE. Prior to the dose administration, all the animals were subjected to overnight food deprivation but maintained their free access to water. The normal control group (*n* = 5) received distilled water (10 mL/kg), while the tested group (*n* = 5) of experimental mice was administered a single oral dose of CLAE at a concentration of 2000 mg/kg body weight (b.w.) through oral gavage. After the treatment, all mice were kept under careful observation for the first 30 min, episodically for the first 6 and 24 h and every day for the period of 14 days to record mortality, autonomic and central nervous system (CNS) changes and other signs of acute toxicity. On the last day, all experimental animals were killed with cervical dislocation, their vital organ (heart, liver and kidney) weight was measured, and histopathological examination was performed. Prior to sacrifice, their body weight was also monitored [39,40]. 

### 2.9. Subacute Toxicity Study

The repeated-dose toxicity of fifteen experimental mice (weighing 25–35 g) was demonstrated through their daily oral exposure to the distilled water (vehicle, 10 mL/kg b.w.) or CLAE (400 and 800 mg/kg b.w.) for a time-frame of 28 days (*n* = 5 per group). All the animals were examined for several behavioral and physical variations daily, whereas their body weights were recorded at the end of the study. On the 29th day, the surviving mice were weighed and killed with cervical dislocation after a short fasting period (12 h). For biochemical parameters, the blood samples were collected in different tubes without ethylenediaminetetraacetic acid anticoagulant (EDTA). Blood serum was extracted by centrifugation at 3000 rmp for 10 min at 5 °C and then stored at −8 °C for biochemical analysis employing an automated URIT chemistry analyzer (URIT-8021A, Guangxi, China). The blood samples for hematological tests were put in various tubes with EDTA. An auto hematology analyzer (Rayto RT-7200, Shenzhen, China) was used to perform the hematological analysis of blood samples. After the collection of blood, various vital organs such as the heart, liver and kidneys were removed and preserved in formalin (10%) for macroscopic analysis. The body weight of tested animals and their separating organ weight were measured using the electronic balance and compared with the control group [41,42].

### 2.10. Cardioprotective Studies 

#### 2.10.1. Isoproterenol-Induced Chronic Myocardial Infarction (MI)

The study for ISO-induced chronic MI was designed for 21 days by following the modified method of Eladwy et al. [43]. A total of thirty male SD rats (weighing 200–250 g) were randomly allotted to five groups (*n* = 6 in each group) and retained in separate cages. ISO was dissolved in 0.9% normal saline. The control group (Group I) orally received distilled water (10 mL/kg) once daily for 21 days. The intoxicated control group (Group II) received 5 mg/kg/day ISO (subcutaneously) for 10 days (initiated from the 12th experimental day until the 21st day). The reference-drug-administered group (Group III) received verapamil orally at 5 mg/kg/day for 21 days. The extract-treated groups received CLAE orally at a concentration of 75 (Group IV) and 150 (Group V) mg/kg/day for 21 days. For 10 consecutive days (beginning from the 12th experimental day until the 21st day), the subcutaneous injection of ISO (5 mg/kg) was given to the animals of groups III, IV and V after one-hour pretreatment with verapamil and CLAE (75 and 150 mg/kg). 

#### 2.10.2. Blood Collection and Histopathological Examinations

After one day of the last ISO dose administration, the animals were exposed to ketamine (intraperitoneally, 40 mg/kg) and induced anesthesia, and blood samples were collected from the retro-orbital sinus for the determination of biochemical markers in the coagulant and anticoagulant (EDTA) tubes. For the separation of serum and plasma from blood, the centrifugation was performed at 4000 rmp for 10 min at 5 °C and then stored at −8 °C for further analysis. The rats were killed by using the process of cervical dislocation for the removal of heart tissues. For histopathology investigations, the cardiac tissues were fixed in formalin (10%). The fixed heart tissues were dehydrated with 70–100% graded alcohol and embedded into the paraffin blocks at 56 °C. The specimen sections (about 5 µm thick) were cut, placed on the microscopic glass slides, and finally achieved the hematoxylin and eosin (H&E) staining of these tissues. The photomicrographs were taken with the help of a Nikon alphaphot-2 microscope (Nikon, Minato, Japan) attached to the Omax 5 MP digital camera (OMAX, Gyeonggi-do, South Korea), using the ToupView software version 3.7.7817 (ToupTek Photonics, Hangzhou, China). The final body weight of all the animals was measured prior to anesthesia [44,45].

#### 2.10.3. Measurement of Cardiac Biometric Indices 

The cardiac hypertrophy was evaluated with the measurement of various biometrical indices such as heart weight, heart weight index (heart weight/body weight ratio), tibia length index (heart weight/tibia length ratio), tail length index (heart weight/tail length ratio) and heart surface area by using a micrometer caliper [45,46]. 

#### 2.10.4. Estimation of Heart Biochemical Markers

The cardiac biomarkers and lipid profile of blood serum were used to quickly find out the chronic MI in animals. The cardiovascular and inflammatory blood serum biomarkers including alanine transaminase (ALT), aspartate transaminase (AST), creatine phosphokinase (CPK), creatine kinase-MB (CK-MB), lactate dehydrogenase (LDH), troponin I (cTnI), nitrate/nitrite (NO) levels and interleukin-6 (IL-6) and lipid-profile-measuring markers such as high-density lipoprotein (HDL), low-density lipoprotein (LDL), triglycerides (TG) and total cholesterol (TC) levels were estimated by using commercially available enzymatic kits (Creative Diagnostics, Shirley, NY, USA) and quantified at 340 nm by using spectrophotometry as per the instructions of the manufacturer [45,47]. 

### 2.11. Behavior Studies

#### 2.11.1. Experimental Design

The acute NS-induced model of Samad et al. [20] was used with slight adjustments to assess the protective outcomes of CLAE following the NS-induced behavioral and cognitive deficits in SD male rats (weighing 150–200 g). For this experiment, thirty arbitrarily selected rats were divided into five groups (*n* = 5 in each group). The control group (Group I) was treated with distilled water (10 mL/kg) and not exposed to NS during the experimental duration. Group II also received distilled water (10 mL/kg) and served as an acute stress group. Groups III, IV and V were titled as test groups. All the tested groups were administered orally with CLAE at the concentration of 100 (Group III), 200 (Group IV) and 300 (Group V) mg/kg b.w. once daily for 14 days and continued until the completion of behavioral tests (day 26) by using the oral gavage. All the rats of groups II, III, IV and V were subjected to NS for 4 h/day (10:00 am to 2:00 pm) for 3 days (on the 15th, 16th and 17th day). The commencement of various behavioral activities such as the open field test, elevated plus maze, forced swim test, novel object recognition and Morris water maze test was monitored from day 18 after exposure to NS. These animals were acclimatized to the environment of the experimental room prior to the start of every behavior test. All behavioral studies were conducted between 8:00 am and 6:00 pm. The experiments were performed in a well-mannered atmosphere to minimize the consequences of external biases (direction and time). An appropriate wash-out period was managed between study treatments to depreciate the carryover effects of one behavior test on the next. The scheme of designed experiments and study timeline was discussed in Supplementary Figure S1. 

#### 2.11.2. Noise Stress Induction Procedure 

Generator noise was recorded, and speakers were used for its amplification in a separate room to train the animals to NS. The speakers were adapted to being 32 cm above the apex of the animal cages. The noise level was set to 100 dB (3 KHz). A sound level meter (Smart Tools Co., Saitama, Japan; Range: 80–120 dB) with an accuracy of 30 Hz (±1.3 dB) was used to monitor the sound intensity [20].

### 2.12. Behavior Studies for Anxiety

#### 2.12.1. Open Field Test (OFT)

For the OFT, a square-shaped (80 × 80 cm) acrylic material-made apparatus was used. It had an open arena fenced by 35 cm high walls to prevent the evacuation of animals. The exploratory behavior of the animals was examined individually in the open field for 5 min. The parameters including the number of central zone entries and time spent in this zone were regarded as a sign of lower anxiety. ANY-maze software version 6.1 (Stoelting Co., Wood Dale, IL, USA) was used for the interpretation of results [48]. 

#### 2.12.2. Elevated Plus Maze (EPM) Test

In the EPM test, the apparatus was composed of four arms. Two were closed, and two arms were exposed. These arms formed the plus-shaped maze. The maze had a length and width of 110 cm and 10 cm, respectively. It was positioned 50 cm above the ground. Both the closed and open arms crossed at a central platform (10 × 10 cm center square) that provided access to every arm. Individually, the animals were examined for 5 min in the EPM apparatus by introducing the animal in the middle of the maze while facing an open arm. Time spent on the open arms, as well as the number of entries made into this arm, was measured using the ANY-maze software version 6.1 to explore the anxiolytic potential of CLAE [49]. 

### 2.13. Behavior Studies for Depression 

#### Forced Swimming Test (FST)

FST is most frequently used to evaluate rats’ behavior based on desperation in uncomfortable environments [50]. A transparent plexiglass cylinder 35 cm in height and 23 cm in diameter was filled with water (25 ± 2 °C) to the point where each animal could float without hitting the bottom of the cylinder. Each animal was put through a 5 min test after being released into the testing environment. While an animal moved with at least two limbs, when swimming, it was believed to be in motion. However, when the animal was upright and making just the slightest movements to maintain its head above water, it was considered to be immobile. Reduced mobility in animals is a symptom of depressive-like behavior and the antidepressant effect of CLAE was predicted with the help of ANY-maze software version 6.1 [6]. 

### 2.14. Behavior Studies for Memory and Learning

A one-day (day 21) washout period was given to the animals after the forced swimming test to reduce any potential confounding effects of water (aversive stimuli) on the consequences of the upcoming behavior test. 

#### 2.14.1. Novel Object Recognition (NOR) Test

As healthy rodents exhibit a strong tendency to interact with unique surroundings, the NOR test is frequently used to measure the rodent’s cognition in various models of CNS diseases [51]. The NOR task of experimental animals was investigated by exploring them to both known and new objects in an acrylic open field apparatus (80 × 80 cm). The rats were initially familiarized with the testing apparatus for one hour on the experimental day. The test was conducted in two sessions. The animals were allowed to inspect two identical objects (glass jars filled with white cement) for 10 min during the first session. The interest of each animal in the recognition of new and old objects was observed for 5 min during a second session in which one object was swapped out with a new object. These parameters were observed by means of ANY-maze software version 6.1 for the calculation of the discrimination index (DI), which, as it increases, indicates improved recognition memory [50].

DI = [(Time spent with the novel object)/(Time spent with the novel object + Time spent with the old object)]

#### 2.14.2. Morris Water Maze (MWM) Test

The modified hidden platform-based version of the MWM test was utilized to assess the effects on the spatial memory of rats [52]. The maze comprised a white-opaque-water (23 ± 2 °C)-filled greyish circular fiber-glass tank with a diameter and height of 150 and 50 cm, respectively. On the basis of four poles (N, S, E and W), the water maze was distributed into four quadrants (NW, SW, SE and NE). A wooden escape platform (10 × 10 cm) was submerged 2 cm below the water’s surface and positioned in the center of the SW quadrant. The geometrical indicators (▲, □, ○ and ▬) were displayed on the tank’s interior surface as proximal cues, while the same distal cues were shown on stands positioned around the tank to aid the animals in navigation. The MWM test comprised two phases. The first one consisted of a 3-day spatial acquisition phase with four training trials each day separated by a 30 min interval. As previously stated by Liu et al. [53], an acclimatization session was held on the first day. The trial ended when the animal reached the platform or 120 sec had passed, and if the rat could not detect the platform, the experimenter directed the rat onto the platform. Each animal was permitted to rest on the platform for 20 sec after reaching it. On the very next day after the completion of the training phase, the probe trial (second phase) started with the removal of the platform. Each rat was given 120 sec to swim in the maze water. The behavioral data of both acquisition and probe tests were recorded for the determination of spatial memory. The latency to reach the platform, number of entries in the targeted quadrant and time spent in this quadrant were monitored with the help of a Logitech HD camera, and the outcomes were measured by using the ANY-maze software version 6.1 [50,54].

### 2.15. LC-ESI-MS/MS Analysis

The bioactive constituents in CLAE were identified through LC-ESI-MS/MS analysis. For this purpose, an ion trap linear mass spectrometer equipped with an electrospray ionization (ESI) source was used (LTQ XL model, Thermo Scientific, Waltham, MA, USA). The sample solution was prepared from the ultra-sonication of plant extracts (20 mg/mL) in methanol (LC-MS grade) at 25 °C for 5 min followed by centrifugation at 12,000 rpm for 10 min. Finally, the supernatant was collected and filtered using a nylon syringe filter (0.45 μm). About 50 μL of each sample stock solution was diluted with methanol, and the final volume was adjusted up to 1.0 mL. This solution was directly inserted into the ESI interface using a syringe pump at a 10 μL/min flow rate. The typical operational conditions optimized for negative and positive ionization spectra were established as follows: the capillary voltage, 4000 V; the capillary temperature, 280 °C; the auxiliary gas (N_2_) flow rate at 6.0 arbitrary units (a.u); the sheath gas (N_2_) flow rate at 17 a.u; and the scanning mass range set at 50 to 2000 *m*/*z*. Depending on the nature of parent molecular ions, the ESI-MS fragmentation was obtained using the collision-induced dissociation energy of 2–15%. The ESI-MS/MS data acquisition was performed utilizing Xcalibur control version 2.0 software (Thermo Scientific, Waltham, MA, USA). The MS spectral data, MS/MS fragmentation pattern and molecular masses of various query mass spectra were compared with reference mass spectra in the published literature, virtual compound libraries and scientific databases including the Human Metabolome Database (HMDB), MassBank and PubChem for the annotation of major phytoconstituents analyzed from CLAE [55,56]. 

### 2.16. Drug-Likeness and ADMET Predictions

Except amino acids, the SMILES (Simplified Molecular Input Line Entry System) of other 48 phytochemicals identified through LC-ESI-MS/MS were subjected to online available web servers including pkCSM (http://biosig.unimelb.edu.au/pkcsm/prediction; accessed on 11 August 2022) and SWISS ADME (http://www.swissadme.ch; accessed on 11 August 2022) for the evaluation of the drug-like nature and absorption, distribution, metabolism and excretion and toxicity (ADMET) parameters of these targeted compounds by keeping in view the already established principles of Lipinski’s rule of five [57,58,59]. 

### 2.17. Molecular Docking Approach

The detailed methodology of Sirous et al. [60] was executed for the docking analysis of selected 44 compounds using Maestro interface version 12.9 of Schrodinger suite 2021 (Schrödinger, Inc., New York, NY, USA).

#### 2.17.1. Ligand Preparation

For ligand preparation, the two-dimensional (2D) conformations of selected compounds were occupied from PubChem and processed in Maestro’s LigPrep module for ligands ionization, minimization and optimization. This module’s Epik tool was used for the generation of ligand ionization forms at the cellular pH of 7.0 ± 2.0. To design the lowest energy conformer of ligands, the OPLS (optimized potentials for liquid simulations) 4 force field was applied through the module for ligands minimization and optimization. 

#### 2.17.2. Protein Preparation

By considering the abnormal expression importance of 3-hydroxy-3-methyl glutaryl Coenzyme A (HMG-CoA) reductase and nitric oxide synthase (NOS) in cardiovascular ailments including MI [61] and AChE in brain disorders [62], these enzymes were selected as major target proteins for docking analysis. The X-ray crystal structures of hepato-selective HMG-CoA reductase (PBD ID 2R4F), human endothelial NOS (PBD ID 1M9R) and human AChE (*h*AChE, PBD ID 4EY6) were extracted from the Protein Data Bank (PBD). Antecedent to docking simulations, each target protein was put through Maestro’s protein preparation wizard in such a manner that it incorporated the random hydrogen atoms and removed all water molecules (solvents) from the X-ray structure. This module was also processed by assigning the bond orders, creating the disulfide bonds, filling the missed side chains and loops and generating the protonation state with the help of the Epik tool of protein structures for ligands at a pH of 7.0 ± 2.0. By the following process, PROPKA was used to optimize protein structures at pH 7.0. For the purpose of energy minimization and protein structural shape optimization, restricted minimization was carried out using the OPLS4 force field. 

#### 2.17.3. Receptor Grid Generation and Molecular Docking

The Maestro receptor grid-generating module defined the active regions of protein structures for molecular docking. With the use of a literature review or a selection of ligands that have already bound to certain proteins, a cubical-shaped grid box for every protein was established. The grid box’s length was adjusted up to the length of 16 Å. On the basis of the van der Waals radius of nonpolar protein atoms with partial atomic charge cutoffs of 0.25 Å, the potential of the receptor’s nonpolar components was reduced to a scaling factor of 1.0 Å. Finally, the optimized ligand and protein molecules were exposed to the extra precision (XP) mode of the Glide module in the Maestro interface of the Schrodinger suite for molecular docking by utilizing a pre-generated grid file for the receptor. The van der Waals radii of docked ligands with a partial charge cutoff of 0.15 Å were adjusted with a 0.80 Å scaling factor. In order to produce conformers, the sampling of ligands was modified for flexible docking with the incorporation of sample nitrogen inversions, sample ring conformations and bias sampling of torsions for all predefined functional groups. The addition of Epik state penalties to the docking score was also turned on. Finally, the glide XP-scoring function was used to sort out the most favorably docked ligands. A visual inspection and glide score values less than −6.5 kcal/mol^−1^ were used to pick top-ranked compounds. For the calculation of binding energies, the docking verdicts were submitted to the Prime MM-GBSA (molecular mechanics with generalized born and surface area solvation) module, which used the VSGB (variable-dielectric generalized born) solvation model with the OPLS4 force field to compute the binding energies of ligand–protein interactions. 

#### 2.17.4. Inhibition Constant (Ki)

The following equation was used to calculate the inhibition constant using the binding free energy of a ligand previously produced from the Prime MM-GBSA.

ΔG = −RT(lnKi) or Ki = e(−ΔG/RT)

where ΔG is the ligand’s binding free energy, R is the gas constant (cal·mol^−1^·K^−1^) and T is 298 Kelvin (room temperature). 

### 2.18. Network Analysis

#### 2.18.1. Screening of Ingredients-Related and Disease-Associated Potential Targets

The protein targets associated with top-docked phytochemicals were predicted through DrugBank and Swiss Target Prediction tools [63]. An online platform, DisGeNET (https://www.disgenet.org; accessed on 23 August 2022), was screened for the identification of the corresponding gene targets implicated in MI, anxiety, depression and memory and learning deficits with keywords “myocardial infarction”, “cardiac hypertrophy” [64], “anxiety disease” [65], “mental depression”, “depressive disorder” [66], “memory impairment” and “memory loss” [67]. In this study, all “Homo sapiens” targets were selected. The targeted results were then placed into the VarElect (https://ve.genecards.org; accessed on 24 August 2022) database for the generation of a specific score that highlights an extensive portrayal between disease phenotype and genetic correlation. A total of 200 targets were reserved to decipher the intersection analysis of biologically active entities and disease targets by using an online toolkit “Venny” (https://bioinfogp.cnb.csic.es/tools/venny/; accessed on 24 August 2022). A Venn diagram was drawn to illustrate the correlative targets of top-ranked compounds and disease target proteins. These correlative targets were processed further for KEGG (Kyoto Encyclopedia of Genes and Genomes) and the Gene Ontology (GO) enrichment analysis [63].

#### 2.18.2. Enrichment Analysis and Network Construction

ShinyGo v0.76 was used to extract the GO and KEGG enrichment analysis with Homo sapiens and a 0.05 false discovery rate (FDR) as the cutoff criteria. The analysis and construction of the protein–protein interaction (PPI) network, compound target disease (CTD) network and compound target pathway (CTP) network were achieved in Cytoscape 3.9.1 (National Institute of General Medical Sciences (NIGMS), Bethesda, MD, USA) [68,69,70]. 

### 2.19. Statistical Analysis

All in vitro bioassays were conducted thrice. The statistical analysis was conducted on in vivo studies using one-way ANOVA, followed by Dunnett’s multiple comparison test. To evaluate the escape latencies observed in MWM, a two-way ANOVA followed by Dunnett’s multiple comparison tests was used. All data were expressed as mean ± standard deviation (SD) with *p* ˂ 0.05 considered statistically significant. The Graph Pad Prism (version 8.1) program was employed for statistical analysis and plotting of all graphs.

## 3. Results

### 3.1. Determination of TPC, TFC and Antioxidant Capacities

Based on linear regression lines of gallic acid (y = 0.0152x + 0.0464) and quercetin (y = 0.0223x + 0.0275), it was found that the aqueous leaf extract of *C. lancifolius* exhibited higher TPC (67.70 ± 0.15 µg GAE/mg) than the TFC (47.54 ± 0.45 µg QE/mg) (Table 1). The antioxidant capability of CLAE revealed the inhibition of DPPH radical formation with an IC_50_ value of 16.66 ± 0.42 µg/mL as compared to the standard ascorbic acid (IC_50_ = 8.67 ± 1.83 µg/mL). In the phosphomolybdenum and potassium ferricyanide colorimetric assay, the TAC and TRP ability of CLAE was evaluated at four tested concentrations (31.25 to 250 µg/mL) by applying the calibration curves of ascorbic acid (y = 0.0014x + 0.1754) and gallic acid (y = 0.0006x + 0.1712), respectively. At the concentration of 250 µg/mL, the observed TAC of CLAE was 77.33 ± 0.41 µg AAE/mg, while at the same concentration, the TRP potential exhibited by CLAE was 79.11 ± 0.67 µg GAE/mg (Table 1, Supplementary Figure S2). 

### 3.2. AChE Inhibitory Activity

We identified that the CLAE demonstrated dose-dependent AChE inhibition with an IC_50_ value of 110.13 ± 1.71 µg/mL, while the examined IC_50_ value for the standard galantamine was 6.48 ± 1.29 µg/mL (Table 1). The percentage enzymatic inhibition of the extract is tabulated in Supplementary Figure S2.

### 3.3. Single-Dose Acute Toxicity

No mortality was observed in experimental mice after a single dose (2000 mg/kg) of CLAE during a 14-day toxicity trial. The extract-treated group did not display other treatment-related toxic signs such as impairment in food and water consumption, body temperature and breathing rate, behavior alterations and skin effects throughout the 6 h and 24 h observation period. Therefore, the LD_50_ of CLAE was determined to be greater than 2000 mg/kg. However, the final body weight and the weight of two separate organs (heart and kidney) in CLAE-administered animals were slightly higher than the control group animals (Table 2). Regarding histopathological analysis, the CLAE-treated animals exhibited normal architecture of heart and kidney cells. In contrast, mild infiltration of inflammatory cells was observed in the liver tissues of these animals when compared with the control group (Supplementary Figure S3).

### 3.4. Repeated-Dose Subacute Toxicity

All the animals treated with 400 and 800 mg/kg of CLAE survived throughout the entire 28-day study period. No behavioral abnormalities were seen at the end of the treatment period in all experimental groups. In comparison with control group mice, the CLAE-treated animals (400 and 800 mg/kg) illustrated a non-significant (*p* > 0.05) reduction in their body weight and the weight of vital body organs including the heart, kidney and liver (Table 2). 

The findings of various biochemical and hematological tests performed on extract-treated animals and the control group are presented in Table 3. About biochemical parameters, the animals of CLAE-tested groups displayed a statistically insignificant (*p* > 0.05) escalation in some heart, kidney and liver markers (albumin, A/G ratio, total proteins, ALP, ALT, AST, total bilirubin, uric acid, creatinine, TC, triglycerides, sodium, potassium and chloride levels) as compared to the control group. Likewise, the animals treated with 400 and 800 mg/kg CLAE registered a non-significant (*p* > 0.05) elevation in some hematological parameters, including red blood cells (RBCs), hemoglobin (Hb), hematocrit, white blood cells (WBCs), neutrophils, lymphocytes and platelets, when compared with the control group. At the 400 mg/kg dose, a non-significant (*p* > 0.05) decrease in eosinophil count was observed in comparison with the control group. The mean corpuscular volume (MCV), mean corpuscular hemoglobin (MCH) and mean corpuscular hemoglobin concentration (MCHC) values in mice at both concentrations of CLAE remained close to the normal group. All elevated biochemical and hematological markers appeared within the reference range in the extract-administered groups.

In the case of histopathological outcomes, the H&E assessment of CLAE-administered animals revealed some treatment-related alterations in the kidney and liver architecture of mice at 400 and 800 mg/kg compared with the control group animals. However, the heart muscles of mice exhibited no histopathological abnormalities in both CLAE-treated categories. The kidney sections of both extract-treated groups exposed the degenerative behavior of epithelial cells around the tubules, the existence of cells in the renal tubular lumen and mild inflammatory cell infiltration. In liver tissues, moderate inflammatory cell infiltration was observed in experimental mice at a 400 mg/kg dose of CLAE, while these inflammatory infiltrates were severe in 800 mg/kg group animals (Supplementary Figure S3).

### 3.5. ISO-Induced Chronic Myocardial Injury

Among the five experimental groups, the control group rats were found to be normal and healthy. No mortality occurred in the group of rats treated with standard drug verapamil. Up to the 15th day, the rats of CLAE-administered groups (75 and 150 mg/kg) appeared healthy and showed spontaneous body movements. However, a few rats developed fatigue and shortness of breath during the last six days. As a consequence, the rats seemed unhealthy and less impulsive than before. Due to this mild sickness, one rat from each group IV (75 mg/kg CLAE + ISO) and V (150 mg/kg CLAE + ISO) expired. Meanwhile, three rats from the ISO-intoxicated group died due to myocardial injury. The physical activity of ISO-intoxicated group rats decreased after the 3rd ISO injection. It entirely diminished after the 10th injection with severe shortening of breath, fatigue and weight loss. Therefore, on average, three rats from each experimental group were sacrificed and put for further analysis on the 22nd experimental day.

**Figure 1:**
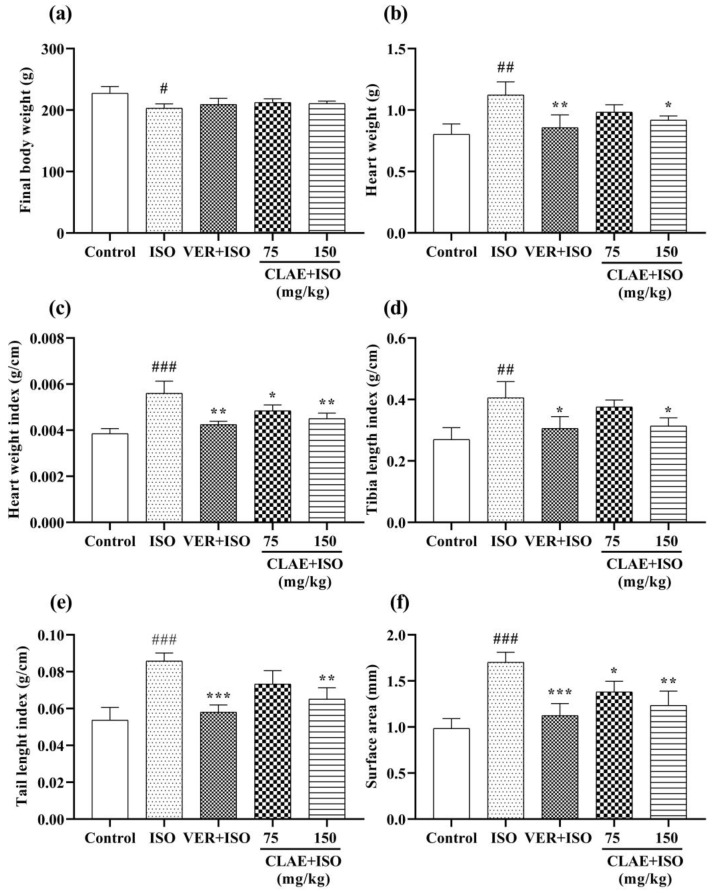
Effect of CLAE (75 and 150 mg/kg) on body weight and heart biometrical indices as compared with intoxicated (ISO-alone treated) group. Final body weight (**a**); Heart weight (g) (**b**); Heart weight index (g/cm) (**c**); Tibia length index (g/cm) (**d**); Tail length index (g/cm) (**e**); and Heart surface area (mm) (**f**). ^#^ *p* < 0.05, ^##^ *p* < 0.01 and ^###^ *p* < 0.001, comparisons between control and ISO-alone treated groups; * *p* < 0.05, ** *p* < 0.01 and *** *p* ˂ 0.001, comparisons between verapamil (standard drug) and ISO-alone treated groups; * *p* < 0.05 and ** *p* < 0.01, comparisons between CLAE-treated (75 and 150 mg/kg) and ISO-alone administered group.

The body weight of all experimental groups was recorded upon the completion of the treatment period. Compared with the control group, there was a significant (*p* ˂ 0.05) decrease in the body weight of the ISO-intoxicated group. In contrast, no significant (*p* > 0.05) difference was seen among the body weights of verapamil and CLAE-treated groups (75 and 150 mg/kg) as compared to the ISO-treated model group. All biometric indices such as heart weight, heart weight index, tibia length index, tail length index and heart surface area were significantly (*p* ˂ 0.01, *p* ˂ 0.001 vs. control group) elevated in the ISO-alone treated group. However, these parameters were reduced significantly (*p* ˂ 0.05, *p* ˂ 0.01, *p* ˂ 0.001 vs. ISO-alone treated group) in verapamil- and CLAE (150 mg/kg)-administered groups. At the dose of 75 mg/kg, CLAE was found with mild significance (*p* ˂ 0.05 vs. ISO-alone treated group) in the myocardial injury protection of experimental rats (Figure 1). 

**Figure 2:**
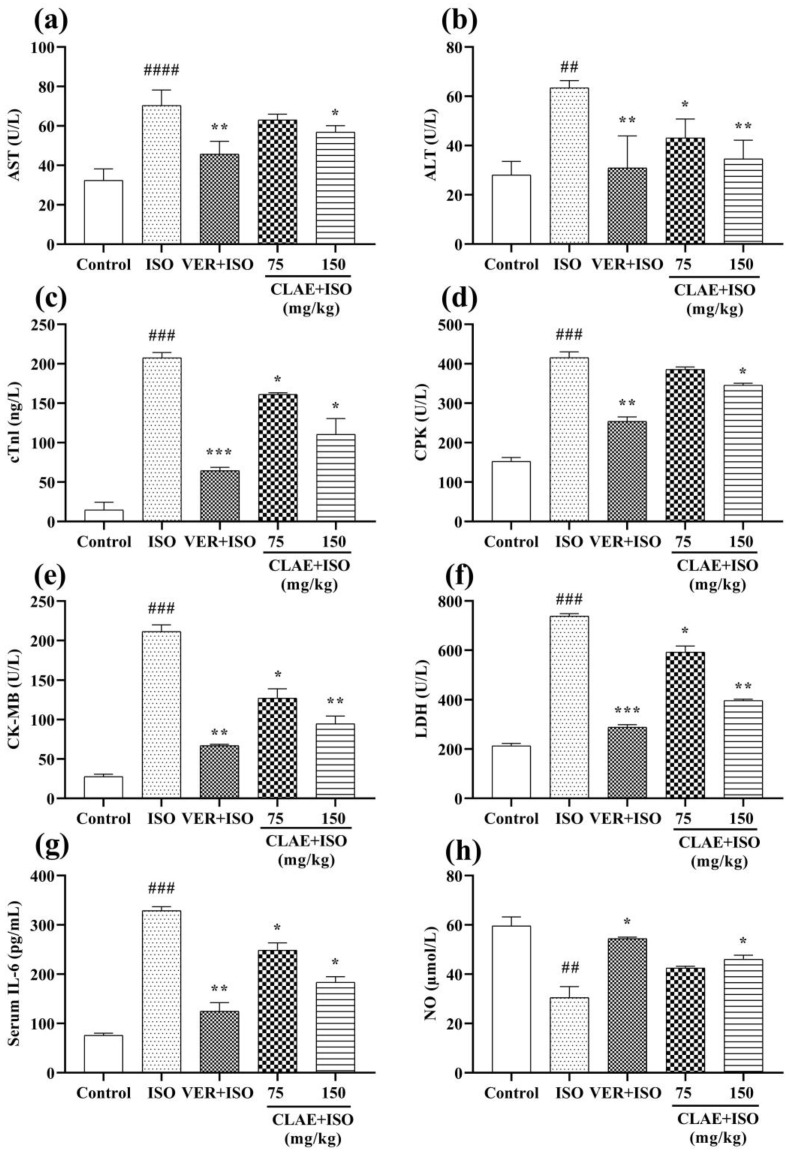
Effect of CLAE (75 and 150 mg/kg) on serum cardiac biomarkers in comparison with intoxicated (ISO-alone treated) group. Aspartate transaminase (AST) (**a**); Alanine transaminase (ALT) (**b**); Troponin I (cTnI) (**c**); Creatine phosphokinase (CPK) (**d**); Creatine kinase-MB (CK-MB) (**e**); Lactate dehydrogenase (LDH) (**f**); Interleukin-6 (IL-6) (**g**); and Nitric oxide (NO) (**h**) levels. ^##^ *p* < 0.01, ^###^ *p* < 0.001 and ^####^ *p* < 0.0001, comparisons between control and ISO-alone treated groups; * *p* < 0.05, ** *p* < 0.01 and *** *p* < 0.001, comparisons between verapamil-treated (standard drug) and ISO-alone treated groups; * *p* < 0.05 and ** *p* < 0.01 comparisons between CLAE-treated (75 and 150 mg/kg) and ISO-alone administered groups.

Systemic elevation in serum cardiac biomarkers and lipid profile was considered the significant diagnostic feature of chronic myocardial infarction. Compared to the control group, the ISO-intoxicated group revealed a significant difference (*p* < 0.01) in the level of serum cardiac markers, including ALT and NO, followed by a significant (*p* < 0.001) rise in CPK, CK-MB, cTnI, LDH and IL-6 levels. A significant (*p* < 0.0001) elevation in the AST level of the ISO-alone treated group was also observed. The ISO-induced alterations in the levels of AST, ALT, cTnI, CPK, CK-MB, LDH, IL-6 and NO were restored significantly (*p* < 0.05, *p* < 0.01, *p* < 0.001) in the serum of verapamil-pretreated rats. In comparison with the ISO-alone treated group, pretreatment of 150 mg/kg CLAE substantially (*p* < 0.05, *p* < 0.01) reduced the level of all cardiac biochemical markers, whereas the restoral of ALT, cTnI, CK-MB, LDH and IL-6 levels was also observed significantly (*p* < 0.05) in the animals pretreated with 75 mg/kg CLAE. Meanwhile, the levels of AST, CPK and NO were decreased non-significantly (*p* > 0.05) in rats at the dose of 75 mg/kg CLAE (Figure 2).

**Figure 3:**
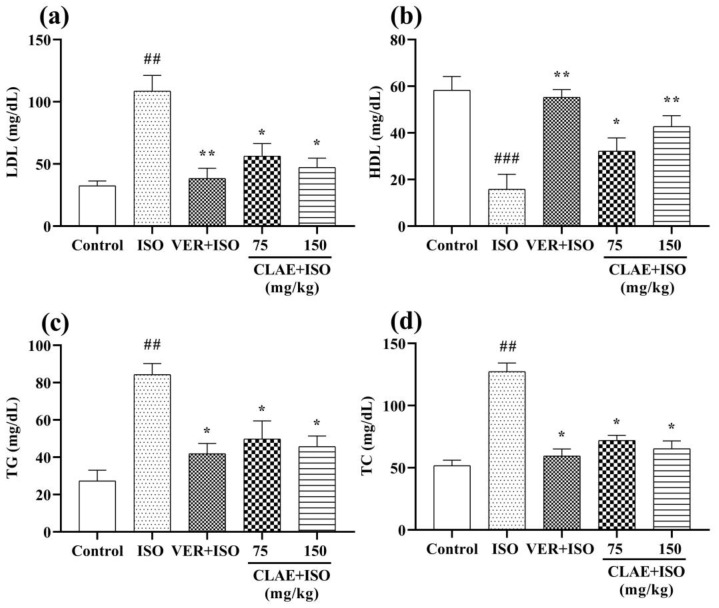
Effect of CLAE (75 and 150 mg/kg) on lipid profile measuring biochemical markers in comparison with intoxicated (ISO-alone treated) group. Low-density lipoprotein (LDL) (**a**); High-density lipoprotein (HDL) (**b**); Triglycerides (TG) (**c**); and Total cholesterol (TC) (**d**) levels. ^##^ *p* < 0.01 and ^###^ *p* < 0.001 comparisons between control and ISO-alone treated groups; * *p* < 0.05 and ** *p* < 0.01 comparisons between verapamil (standard drug), CLAE-treated (75 and 150 mg/kg) and ISO-alone treated groups.

Figure 3 represents the lipid profile (LDL, HDL, TG and TC) of control, ISO-treated and CLAE-administered experimental animals. A noticeable (*p* < 0.01, *p* < 0.001 vs. control group) difference in the blood serum levels of LDL, TG and TC was examined in the ISO-alone treated group. In contrast, LDL, TG and TC levels decreased significantly (*p* < 0.05, *p* < 0.01 vs. ISO-intoxicated group) in verapamil- and CLAE (75 and 150 mg/kg)-treated groups. Moreover, increased (*p* < 0.05, *p* < 0.01) level of HDL was observed in verapamil- and CLAE (75 and 150 mg/kg)-pretreated rats in comparison with the ISO-intoxicated group, while HDL level was significantly (*p* < 0.001) decreased in ISO-alone treatment group rats when compared with the control group rats. 

**Figure 4:**
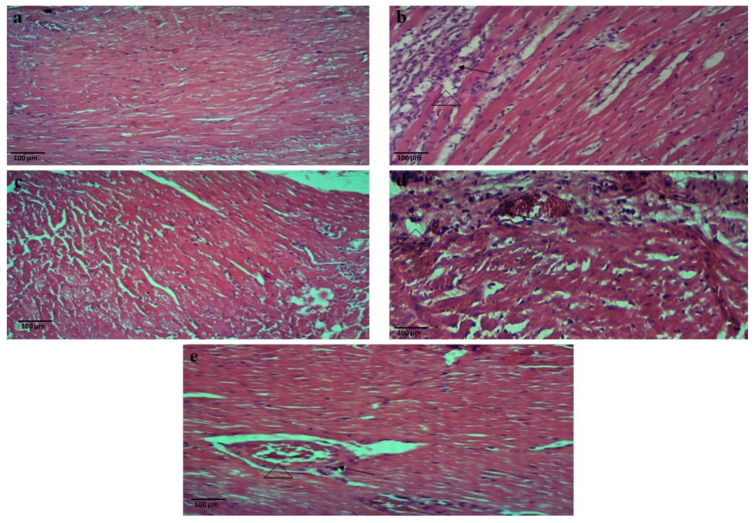
Photomicrographs of myocardium tissues from all experimental treated groups in hematoxylin and eosin staining (10 × magnification). The control group showed normal heart architecture (**a**); The ISO-induced group revealed inflammation accompanied with necrotic myocardial fibers (black arrow) and interstitial edema (triangle) of local tissues (**b**); The verapamil (standard drug)-pretreated group displayed almost normal alignment of cardiac cells similar to control group (**c**); The CLAE low-dose (75 mg/kg)-administered group showed moderate decrease in inflammatory cells infiltration and muscle fiber breakdown associated with necrosis (black arrow) followed by interstitial edema (triangle) (**d**); and the CLAE high-dose (150 mg/kg)-treated group displayed maximal reduction in inflammatory cells infiltration and necrotic myocardial fibers (black arrow) along with interstitial edema (triangle) (**e**) of local tissues, when compared with the ISO-induced group.

In histopathological assessment, H&E staining of the control rat heart displayed normal histoarchitecture of heart muscles with branching fibers and striations. The H&E-stained heart sections of the ISO-alone treated group revealed remarkable alterations in the myocardial tissues, including inflammation (massive inflammatory cell infiltration) accompanied by necrotic myocardial fibers and interstitial edema of local tissues. The H&E sections of all treatment groups, including group III (verapamil + ISO), IV (75 mg/kg CLAE + ISO) and V (150 mg/kg CLAE + ISO), demonstrated less inflammatory cell infiltration and the histology of myocardial fibers was also maintained with reduced signs of muscle fiber breakdown associated with necrosis and intestinal edema as compared to the ISO-intoxicated group, as shown in Figure 4.

### 3.6. Behavior Studies for Anxiety

#### 3.6.1. Open Field Test (OFT)

**Figure 5:**
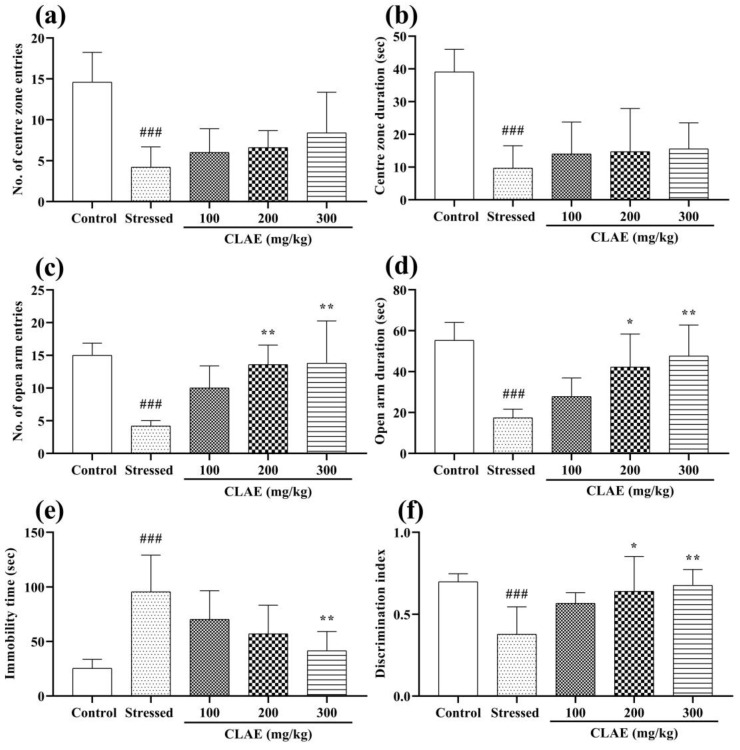
Evaluation of anxiolytic, antidepressant and memory-enhancing activity in rats pretreated with CLAE at the doses of 100, 200 and 300 mg/kg by using the open field, elevated plus maze, forced Swimming and novel object recognition tests. Number of center zone entries (**a**); Time spent in the center zone (**b**); Number of open arm entries (**c**); Time spent in open arms (**d**); Immobility time (**e**); and Discrimination index (**f**). ^###^ *p* < 0.001, comparisons between control and stressed groups; * *p* < 0.05 and ** *p* < 0.01, comparisons between stressed and CLAE-treated groups (200 and 300 mg/kg).

After 60 min of CLAE administration, the animals were subjected to an open field arena to examine the anxiolytic effect of CLAE on the 18th day of study. The OFT results revealed that the number of entries and the time spent in the center zone were reduced dominantly (*p* < 0.001) in stressed group rats compared with the control group. Meanwhile, a non-significant (*p* > 0.05) dose-dependent increased preference was seen in the extract-treated animals (100, 200 and 300 mg/kg) for both center zone parameters as compared to the stressed group showing reduced levels of anxiety (Figure 5a,b). 

#### 3.6.2. Elevated Plus Maze (EPM) Test

The statistical evaluation in the EPM test demonstrated that the stressed group rats revealed a marked decrease in the number of entries (*p* < 0.001) followed by their time spent (*p* < 0.001) in the open arms as compared to the control group. The CLAE-treated groups (200 and 300 mg/kg) significantly (*p* < 0.01) increased their number of entries in the open arms compared to the stressed control group. Moreover, significantly comparable dose-dependent results were also seen in 200 (*p* < 0.05) and 300 mg/kg (*p* < 0.01) extract-treated rats in terms of their time duration in open arms as compared to the stressed group, resulting in excellent anxiolytic activity (Figure 5c,d).

### 3.7. Behavior Studies for Depression

#### Forced Swimming Test (FST)

The experimental rats administered with CLAE were forced to swim for the exploration of the antidepressant potential of the tested extract. Figure 5e shows a significant rise in the immobility time (*p* < 0.001) observed in the stressed group rats compared to the normal rats. However, CLAE-treatment rats showed significant antidepressant activity at 300 mg/kg (*p* < 0.01) but no beneficial effects (*p* > 0.05) at 100 and 200 mg/kg in comparison with the stressed group animals. 

### 3.8. Behavior Tests for Memory and Learning

#### 3.8.1. Novel Object Recognition (NOR) Test

To estimate the memory-improving status of CLAE, the capacity of experimental animals to discriminate a novel object from familiar ones was tested on the 22nd day of the study. The stressed group animals became less capable of distinguishing the novel object from the older ones because of their poor memory, resulting in a decreased discrimination index (*p* < 0.001) compared to the control group animals. Meanwhile, this NS-induced effect was prominently reversed in a dose-dependent manner by CLAE as the animals administered with 200 and 300 mg/kg had enough memory to recognize familiarized objects, and they explored the novel object for a longer period of time (Supplementary Table S1). Thus, the discrimination index was increased significantly in 200 (*p* < 0.05) and 300 (*p* < 0.01) mg/kg treated animals compared to the stressed group animals (Figure 5f). At 100 mg/kg, the rats demonstrated no significant effects. The track plots of random animals to explore the novel object from each group are shown in Supplementary Figure S4. 

#### 3.8.2. Morris Water Maze (MWM) Test

**Figure 6:**
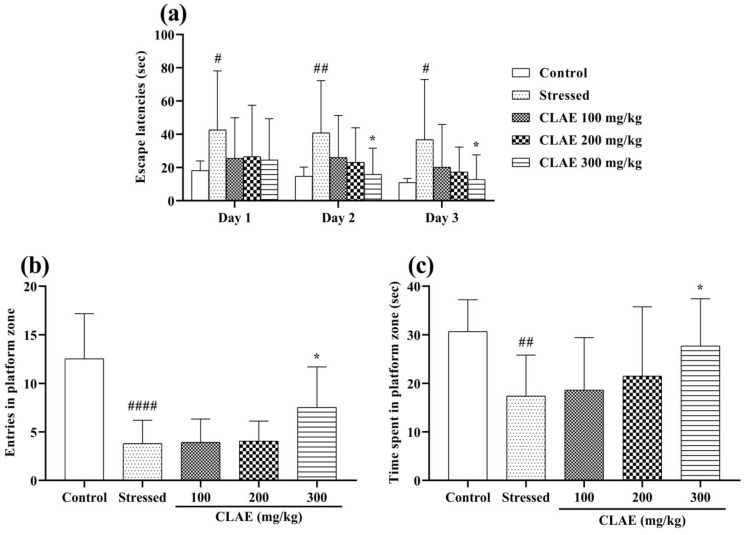
Assessment of learning and memory enhancement effect in CLAE (100, 200 and 300 mg/kg) pretreated rats through Morris water maze test. Escape through latencies (**a**); Number of entries in platform zone (**b**); and Time spent in platform zone (**c**) were observed and compared to the stressed group. ^#^ *p* < 0.05, ^##^ *p <* 0.01 and ^####^ *p* < 0.0001, comparisons between control and stressed groups; * *p* < 0.05, comparisons between stressed and CLAE-treated group (300 mg/kg).

The consequences of the MWM test pronounced the cognition and memory-enhancing activity of CLAE. Two-way ANOVA depicted that the animals of the stressed group had poor platform quadrant memory and thigmotaxic behavior in the water maze, which led to extended escape latencies (*p* < 0.05, *p* < 0.01) on three consecutive training days (days 23–25). The CLAE treatment showed improvement in the remembrance of the platform quadrant as the animals given with 300 mg/kg portrayed shorter escape latencies (*p* < 0.05) in a dose-dependent manner compared to the stressed group. However, escape latencies were statistically non-significant (*p* > 0.05) at low doses of CLAE (100 and 200 mg/kg) (Figure 6a). On probe day (day 26), the rats were finally observed in the water maze without a platform. The rats of the stressed group offered a minimum number of entries (*p* < 0.0001) and stay duration (*p* < 0.01) in the platform-positioned quadrant as they faced reduced remembrance of that platform zone in comparison with the control group rats. These impaired memory signs were markedly reversed in the animals given the plant extract, as the rats treated with 300 mg/kg of CLAE revealed a significant increase in the number of entries *(p* < 0.05) as well as their duration of stay (*p* < 0.05) in the zone where the platform was stationed previously. However, the outcomes of their number of entries and prolonged duration in the platform quadrant appeared to be non-significant (*p* > 0.05) at the dose of 100 and 200 mg/kg CLAE as compared to the stressed group (Figure 6b,c).

### 3.9. Phytocompounds Identification Using LC-ESI-MS/MS Analysis

**Figure 7:**
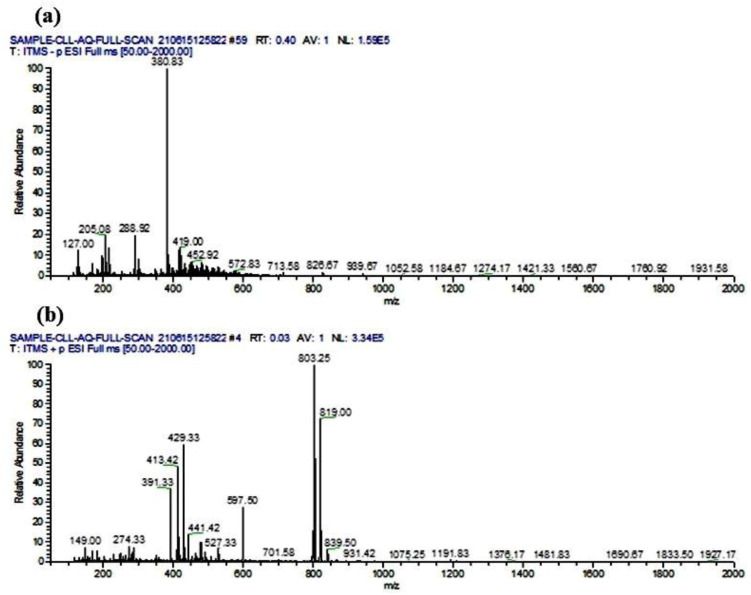
LC-ESI-MS-MS full scan of CLAE in negative ionization mode (50–2000) (**a**) and positive ionization mode (50–2000) (**b**).

The LC-ESI-MS/MS analysis of CLAE predicted a total of 53 phytoconstituents, including 8 alkaloids, 5 amino acids, 3 carboxylic acids, 4 coumarins, 13 flavonoids, 3 lignans, 5 phenolic acids, 3 tannins, 2 terpenoids and 7 other compounds (3-alkylindole, benzoic acid derivative, cinnamaldehyde, glycoside, naphthopyrone, stilbene and triterpenoid saponin). The compounds **1–24** were tentatively identified as deprotonated [M − H]^−^ molecules in the negative ionization mode, whereas the compounds **25–53** appeared as protonated [M + H]^+^ molecules in the positive ion mode of analysis (Table 4). The tentatively identified compounds were characterized based on their retention time, experimental *m*/*z* value, fragmentation profile, calculated mass and chemical class, as corroborated with the previously cited reports and reference libraries [71,72]. The full-scan total ion chromatogram (TIC) of negative and positive ionization modes is given in Figure 7. The chemical structures and mass spectra (MS/MS) of all the identified compounds are shown in Supplementary Figure S5. 

### 3.10. Drug-Likeness and ADMET Studies

Apart from amino acids, the SMILES of the other 48 tentatively identified phytochemicals were submitted to SwissADME and pkCSM programs to investigate their drug-likeness/Ro5 and ADMET properties. The computed drug-likeness outcomes from SwissADME indicated that excluding isoorientin, pachymic acid, flavonol base + 4O, 1MeO, O-Hex-Hex, O-Hex, licoricesaponin G2 and procyanidin C1, all other phytochemicals were in agreement with Ro5 by causing not more than one violation. Except for pachymic acid, the determined HBA and HBD values for other violated compounds were significantly higher than the maximum limit directed by Ro5. In addition, except isoorientin, other compounds violated the Ro5 by surpassing the maximum required molecular weight (MW > 500 g/mol). In the case of lipophilicity, the projected values of log P (XLOGP3) for all identified phytocompounds other than isoangustone A, gancaonin E, maruchantin E and pachymic acid appeared to be less than five, which is according to the directions of Ro5. Most of the analyzed compounds were predicted to have moderate-to-high water solubility and reasonable topological polar surface area (TPSA) values (below the limit of 140 Å2). Besides harringtonine and flavonol base + 4O, 1MeO, O-Hex-Hex and O-Hex, the estimated number of rotatable bonds (RBs) for all the compounds was less than 9, which specified the conformationally stable behavior of the compounds. In line with pan-assay interference compounds (PAINS), some of the phytoconstituents showed 1 alert, which highlighted the specific nature of these compounds (Supplementary Table S2). 

Concerning ADMET parameters, some of the tested phytocompounds were found to have good Caco-2 permeability with log Papp values greater than 0.90 cm/s. A high intestinal absorption (human) percentage was established for most of the identified substances except malic acid, cis-aconitic acid, flavonol base + 4O, 1MeO, O-Hex-Hex, O-Hex and licoricesaponin G2. Because of log BB (logarithmic ratio of brain to plasma concentrations) values more than 0.3, 3,4-dimethoxy-cinnamic acid, 14,15-dehydro-16-epi-vincamine, *p*-coumaraldehyde, 3-indoleacetonitrile, harmaline, 5,7-dimethoxy-4-methylcoumarin and 3,4,5-trihydroxystilbene were determined to have sufficient BBB (blood–brain barrier) permeability. Most of the proposed compounds were categorized as unable to penetrate the central nervous system (CNS), but the majority of them passed the metabolism, excretion and toxicity paradigms. Such limitations can be overcome during the drug development process (Supplementary Table S3). 

Based on the achieved drug-likeness and ADMET prediction analysis, most of the examined compounds have good pharmacokinetic properties except pachymic acid, flavonol base + 4O, 1MeO, O-Hex-Hex, O-Hex, licoricesaponin G2 and procyanidin C1. Therefore, the molecular docking approach was explicitly executed for the remaining 44 naturally occurring compounds of CLAE.

### 3.11. Molecular Docking Approach

**Figure 8:**
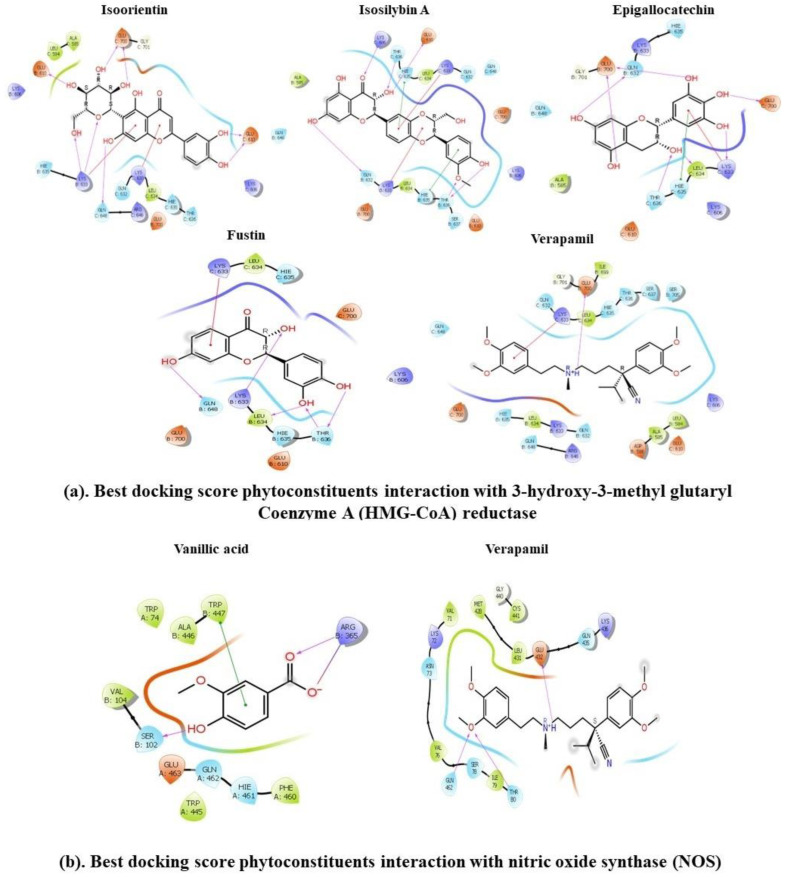

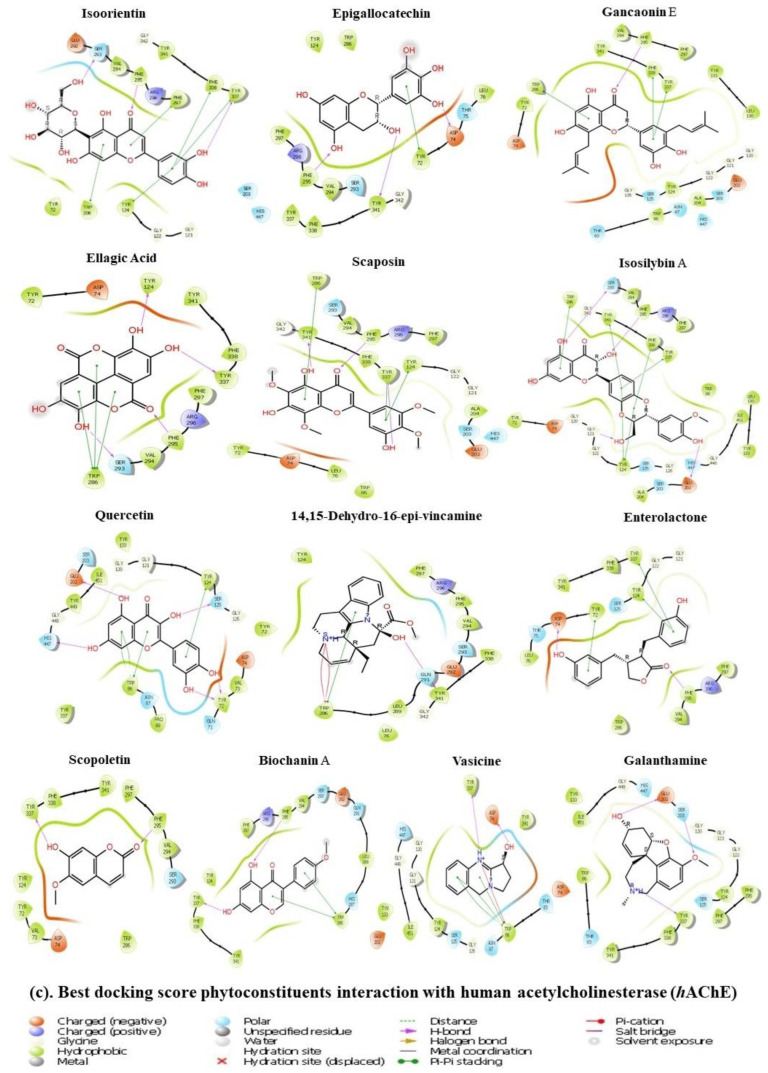
Two-dimensional interaction diagrams between phytocompounds and target proteins. Docking process of isoorientin, isosilybin A, epigallocatechin, fustin and verapamil with HMG-CoA reductase (**a**); Docking alignment of vanillic acid and verapamil with NOS protein (**b**); Molecular docking of isoorientin, epigallocatechin, gancaonin E, ellagic acid, scaposin, isosilybin A, quercetin, 14,15-dehydro-16-epi-vincamine, enterolactone, scopoletin, biochanin A, vasicine and galantamine with *h*AChE (**c**).

Molecular docking simulations were utilized to model the interactions between 44 CLAE constituents and 3 pivotal target proteins: HMG-CoA reductase, NOS and *h*AChE. The lower the binding free energy (ΔG_Binding_), the stronger the ligand–protein interaction. We observed that four ligands among the selected compounds with XP-GlideScore values lower than or equal to −6.5 kcal/mol such as isoorientin (XP-GlideScore: −8.389 kcal/mol, ΔG_bind_: −24.43 kcal/mol), isosilybin A (XP-GlideScore: −7.992 kcal/mol, ΔG_bind_: −29.2 kcal/mol), epigallocatechin (XP-GlideScore: −6.897 kcal/mol, ΔG_bind_: −20.35 kcal/mol) and fustin (XP-GlideScore: −6.849 kcal/mol, ΔG_bind_: −18.5 kcal/mol) showed higher docking scores and lower ΔG_bind_ values towards HMG-CoA reductase than the reference inhibitor verapamil (XP-GlideScore: −3.182 kcal/mol, ΔG_bind_: −14.78 kcal/mol), while one compound, vanillic acid (XP-GlideScore: −6.534 kcal/mol, ΔG_bind_: −13.539 kcal/mol), exposed good interaction towards the NOS receptor when compared with the standard ligand verapamil (XP-GlideScore: −3.539 kcal/mol, ΔG_bind_: −41.97 kcal/mol). On the other hand, 12 top-docked compounds including isoorientin (XP-GlideScore: −15.180 kcal/mol, ΔG_bind_: −67.31 kcal/mol), epigallocatechin (XP-GlideScore: −11.787 kcal/mol, ΔG_bind_: −43.31 kcal/mol), gancaonin E (XP-GlideScore: −11.706 kcal/mol, ΔG_bind_: −31.99 kcal/mol), ellagic acid (XP-GlideScore: −11.403 kcal/mol, ΔG_bind_: −68.58 kcal/mol), scaposin (XP-GlideScore: −11.072 kcal/mol, ΔG_bind_: −63.35 kcal/mol), isosilybin A (XP-GlideScore: −11.052 kcal/mol, ΔG_bind_: −45.74 kcal/mol), quercetin (XP-GlideScore: −10.967 kcal/mol, ΔG_bind_: −23.66 kcal/mol), 14,15-dehydro−16-epi-vincamine (XP-GlideScore: −10.376 kcal/mol, ΔGbind: −43.29 kcal/mol), enterolactone (XP-GlideScore: −9.057 kcal/mol, ΔG_bind_: −49.58), scopoletin (XP-GlideScore: −8.202 kcal/mol, ΔG_bind_: −36.51 kcal/mol), biochanin A (XP-GlideScore: −7.901 kcal/mol, ΔG_bind_: −38.01 kcal/mol) and vasicine (XP-GlideScore: −7.848 kcal/mol, ΔG_bind_: −41.04 kcal/mol) showed very strong docking affinity towards the binding region of *h*AChE in comparison with the standard molecule galantamine (XP-GlideScore: −9.742 kcal/mol, ΔG_bind_: −23.61 kcal/mol). Furthermore, they had well-built interactions with various residues of *h*AChE, including six common amino acid moieties, namely Gly121, Gly122, His447, Tyr124, Ser125 and Ser203. In this study, all of the best-ranked candidates formed strong hydrogen bonds and hydrophobic and electrostatic interactions with the core proteins. Figure 8 and Table 5 depict the details regarding binding energies, polar H-bonding, electrostatic and hydrophobic interactions and ligand–protein interaction diagrams of finally selected hits. Our results indicated the solid binding associations of top-docked ingredients in CLAE with the receptor proteins.

### 3.12. Network Analysis

#### 3.12.1. Screening of Putative Targets

The DrugBank and Swiss Target Prediction databases were reviewed to investigate the probable protein targets for 12 top-docked phytochemicals of CLAE. Due to the unavailability of information in the above databases, epigallocatechin and fustin were excluded from the network analysis. After removing the overlapping targets, a database of 265 candidate target genes was compiled for constituents active against HMG-CoA reductase and NOS proteins. In contrast, a list of 495 target genes was constructed for compounds with *h*AChE inhibitory potential. The chronic-MI-, anxiety-, depression- and impaired-memory-associated gene targets were extracted from the public database DisGeNET. The VarElect database validated gene targets before selecting the top 200 candidate targets for chronic MI, anxiety, depression and memory deficits. To intersect candidate disease targets with phytochemical target genes, a Venn diagram was employed, revealing 19 correlative potential gene targets for chronic MI disease and 30 for anxiety, depression and impaired memory (Supplementary Figure S6). These potentially correlated targets were processed for network construction, GO enrichment and KEGG pathway analysis (Supplementary Tables S4–S7).

#### 3.12.2. GO and KEGG Enrichment Analysis

By utilizing the ShinyGo (v0.76) graphical gene-set enrichment tool, the GO and KEGG pathway analysis of intersected potential genes was performed. The GO biological process revealed a large number of chronic MI target genes for best-docked phytoconstituents with a particular focus on the cellular response to UV-A, response to UV-A, negative regulation of fibrinolysis, regulation of fibrinolysis, embryo implantation, collagen catabolic process, cellular response to UV, extracellular matrix disassembly, collagen metabolic process, response to UV, cellular response to light stimulus, response to light stimulus, cellular response to chemical stress, cellular response to abiotic stimulus, response to oxidative stress, response to radiation, cellular component disassembly, regulation of cell population proliferation and response to oxygen-containing compounds. Bladder cancer, IL (interleukin)-17 signaling pathway, AGE-RAGE signaling pathway in diabetic complications, TNF (tumor necrosis factor) signaling pathway, rheumatoid arthritis, complement and coagulation cascades, proteoglycans in cancer, endocrine resistance, relaxin signaling pathway, Chagas disease, fluid shear stress and atherosclerosis, hypoxia-inducible factor-1 (HIF-1) signaling pathway, serotonergic synapse, microRNAs in cancer, lipid and atherosclerosis, coronavirus disease, Kaposi sarcoma-associated herpesvirus infection, human cytomegalovirus infection and pathways in cancer were the foremost KEGG signaling pathways for chronic MI targets impacted by leading CLAE molecules (Supplementary Figure S7 and Tables S8 and S9).

In terms of the GO biological process, the potential targets of top-ranked CLAE compounds appeared in anxiety, depression and memory impairment, with an emphasis on dopamine uptake involved in synaptic transmission, dopamine uptake, catecholamine uptake, neurotransmitter reuptake, dopamine transport, neurotransmitter uptake, monoamine transport, catecholamine transport, regulation of neurotransmitter levels, cellular calcium ion homeostasis, calcium ion homeostasis, cellular divalent inorganic cation homeostasis, divalent inorganic cation homeostasis, trans-synaptic signaling, synaptic signaling, chemical synaptic transmission, anterograde trans-synaptic signaling and cellular homeostasis. The major KEGG signaling pathways regulated by active CLAE phytochemicals in anxiety, depression and memory impairment were arginine biosynthesis, cocaine addiction, arginine and proline metabolism, amphetamine addiction, dopaminergic synapse, gap junction, serotonergic synapse, calcium signaling pathway, morphine addiction, salivary secretion, relaxin signaling pathway, Chagas disease, amoebiasis, apelin signaling pathway, neuroactive ligand–receptor interaction, Parkinson’s disease, alcoholism, proteoglycans in cancer, Alzheimer’s disease and pathways of neurodegeneration (Supplementary Figure S7 and Tables S10 and S11). 

#### 3.12.3. Network Construction

**Figure 9:**
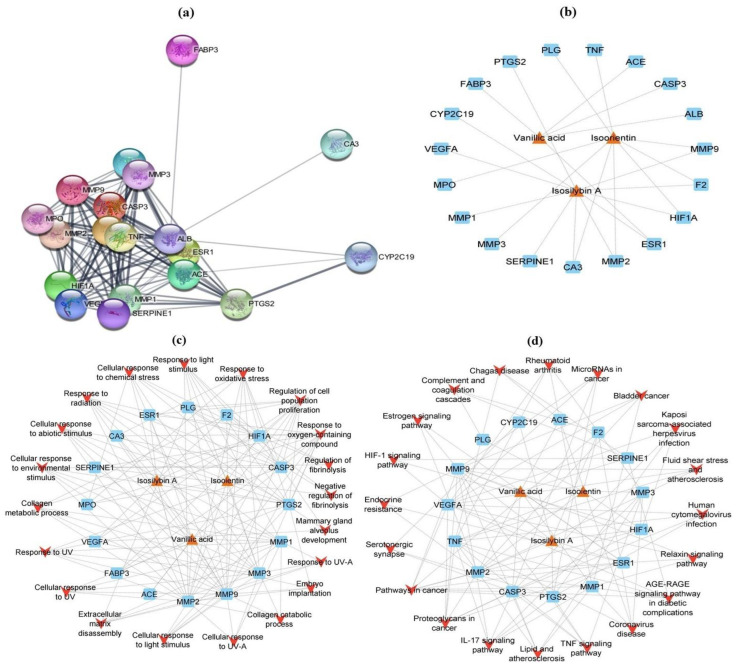
PPI network of myocardial infarction disease (**a**); Phytochemicals—myocardial-infarction-disease-related target gene network (**b**); GO biological processes network of phytochemicals and all target genes related to myocardial infarction (**c**); KEGG pathways network of phytocompounds and myocardial infarction disease target genes (**d**).

In line with retained chronic MI pathogenic targets, Cytoscape version 3.9.1 with stringApp version 1.7.1 built a PPI network, which contained 19 nodes and 116 edges (Figure 9a). Likewise, a CTD network with 22 nodes and 25 edges linking the top-ranked CLAE compounds and chronic MI target genes was also designed in Cytoscape (Figure 9b and Supplementary Table S12). According to a network design study, isosilybin A with a degree of 11 had the highest positive effect on the target genes followed by isoorientin (degree = 9) and vanillic acid (degree = 5). The CTP network design of GO biological process terms for chronic MI target genes involved 39 nodes and 140 edges. According to network construction analysis, vanillic acid exhibited more significant therapeutic effects on target genes with a degree of 9 when compared with isoorientin and isosilybin A (degree = 8). Response to oxygen-containing compounds and regulation of cell population proliferation (degree = 11), response to oxidative stress (degree = 8) and response to radiation, cellular response to chemical stress and response to light stimulus (degree = 7) were the key GO biological process expressions that showed most effective interactions with corresponding targets (Figure 9c and Supplementary Table S13). In agreement with the Cytoscape prediction for KEGG enrichment analysis (Figure 9d and Supplementary Table S14), a CTP network between potential pathways and correlated targets was characterized by 38 nodes and 110 edges. The network study explored that isosilybin A had the strongest positive impact on the disease target genes at a degree of 8 followed by isoorientin and vanillic acid (degree = 7). The major KEGG pathways with the most prominent connections to the target genes and CLAE molecules were pathways in cancer (degree = 9), proteoglycans in cancer (degree = 7), IL-17 signaling pathway (degree = 6) and coronavirus disease, lipid and atherosclerosis, TNF signaling pathway and AGE-RAGE signaling pathway in diabetic complications (degree = 5).

**Figure 10:**
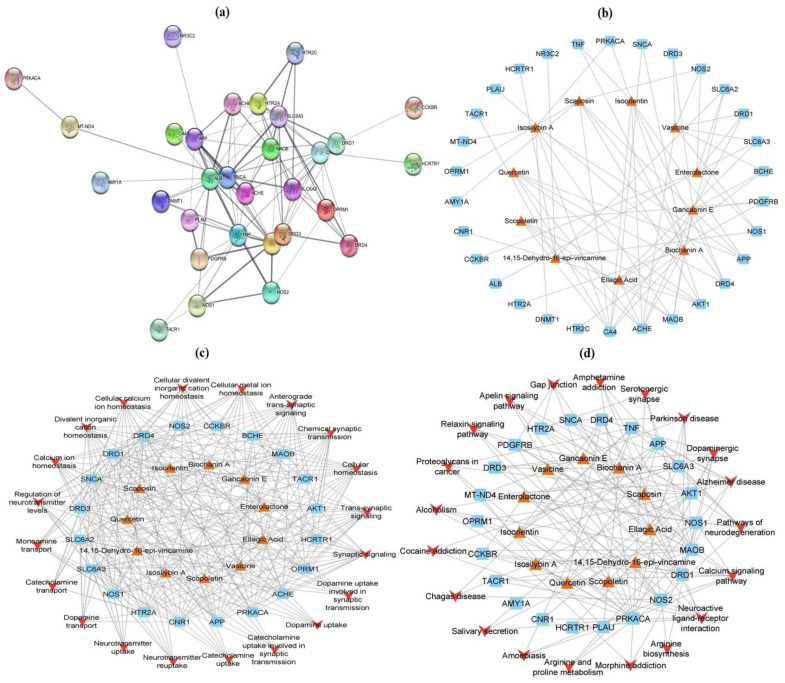
PPI network of anxiety, depression and impaired memory ailments (**a**); Phytochemicals—anxiety-, depression- and impaired-memory-related target gene network (**b**); GO biological processes network of phytochemicals and all target genes related to anxiety, depression and impaired memory (**c**); KEGG channels network of phytocompounds and anxiety, depression and impaired memory problem target genes (**d**).

All the retained pathogenic target genes related to anxiety, depression and memory deficits were tested for protein–protein interactions (PPI) by using Cytoscape (v3.9.1) with stringApp (v1.7.1). These target genes comprised 30 nodes and 94 edges (Figure 10a). In addition, a CTD network between chief CLAE phytochemicals and disease target genes was constructed in Cytoscape with 42 nodes and 76 edges. In accordance with network construction analysis, biochanin A and ellagic acid, with a degree of 9, showed the uppermost positive impact on the target genes, followed by gancaonin E (degree = 8), enterolactone, vasicine, isoorientin and scaposin (degree = 7) (Figure 10b and Supplementary Table S15). After this, a CTP network was also created for GO biological process terms, KEGG signaling pathways, target genes and top-docked CLAE compounds. In the context of GO biological process terms (Figure 10c and Supplementary Table S16), the CTP network included 51 nodes and 272 edges. The network study examined that with degrees of 7, ellagic acid and vasicine affected the disease target genes more favorably as compared to the other phytochemicals. The following terms of the GO biological process displayed a promising positive interaction with target genes and active CLAE constituents: trans-synaptic signaling and synaptic signaling (degree = 16), chemical synaptic transmission, anterograde trans-synaptic signaling and cellular homeostasis (degree = 15). Based on the estimation of KEGG enrichment analysis by Cytoscape (Figure 10d and Supplementary Table S17), the CTP network between corresponding targets and potential pathways was represented by 53 nodes and 143 edges. According to the network analysis, at the degree of 6, ellagic acid and scaposin had the most powerful positive interaction with the disease target genes. The neuroactive ligand–receptor interaction (degree = 9), calcium signaling pathway (degree = 8), Alzheimer’s disease (degree = 7), dopaminergic synapse (degree = 7) and pathways of neurodegeneration (degree = 7) were the major KEGG features with maximum possible connection with the target genes and CLAE chemicals.

## 4. Discussion

Different biological activities of *Conocarpus lancifolius* Engl. provide substantial evidence in favor of its usage as a remedy for various ailments, with noteworthy therapeutic outcomes. Plant species with medicinal properties have been used in various scientific investigations throughout the world. Santos et al. [73] emphasized that over the years, plants have been the leading contributors in the development of several drugs including colchicine, emetine, morphine and vincristine.

### 4.1. TPC, TFC and Antioxidant Capacities

Phenolics is a major class of plant-derived compounds that consume at least one phenol group and have multiple beneficial effects on human health. Phenolic compounds, especially phenols and flavonoids, have very strong antioxidant capacity [50]. In the present work, CLAE exhibited a considerable amount of phenolics and flavonoids. In addition, it also demonstrated prominent radical scavenging ability in DPPH, TAC and TRP assays. Earlier studies have reported a strong correlation between TPC, TFC and the antioxidant potential of plant extracts [11,23]. So, the antioxidant property of CLAE may be attributed to the existence of phenols and flavonoids. Because of their redox potential, the high content of phenolics enhanced the capacity of plants to quench ROS. In the same way, flavonoids have both chelating and scavenging abilities that boost the antiradical potential of plants [50,74,75,76,77]. In contrast, the pharmacological importance of other identified phytoconstituents including alkaloids, tannins and lignans cannot be overlooked because a broad spectrum of antioxidant activities of these bioactive compounds had been already reported in the literature [9]. Therefore, we conclude that further studies should be undertaken to elucidate the particular phytochemicals and their radical scavenging mechanisms. This is because, nowadays, the toxicological effects of synthetic antioxidants have come under criticism. Hence, natural antioxidants, particularly those derived from plants, have gained tremendous importance in modern times [10]. Natural antioxidants are also regarded as “lead” structures because they provide a defense mechanism against the damaging effects of free radicals and serve as radical scavengers, suppressing lipid peroxidation and inhibiting other biochemical processes mediated by free radicals, protecting the human organs against multiple degenerative pathologies attributed to radical reactions such as cancer, Parkinson’s disease, AD, atherosclerosis and MI [12,50]. 

### 4.2. Single-Dose Acute Toxicity

During the evaluation of the safety profile of any medicinal plant, the primary objective is to determine the type and severity of adverse effects, as well as to pinpoint the exposure level at which these effects are manifested. According to the findings of this study on acute toxicity, a single-dose oral administration of CLAE at 2000 mg/kg to mice did not result in any signs of toxicity or death in the tested animals. In addition, there were no significant variations in the final body weight, essential organ weight and histopathological indicators, representing the low-toxic nature of CLAE at a single dose. This was decided by observing the animals episodically for the first 24 h and every day for a period of 14 days. A non-significant rise in the final body weight and in the weights of the heart and kidney followed by an insignificant decrease in the liver weight may have been attributed to normal inter-organ biological variations [78,79], rather than due to the toxic effects of CLAE. Slight variations in the liver cell histology may support the reality that the liver has a key role in the metabolism and excretion of toxic substances, making it more vulnerable to toxicity than other organs [80]. This implies that CLAE has very low toxicity and should be listed in unclassified or category 5 chemicals with an estimated oral LD_50_ of more than 2000 mg/kg, as per the guidelines of the OECD’s Globally Harmonized System (GHS) for the classification of chemical compounds and mixtures [39]. In the acute toxicity protocol of Al-Tameemi et al. [24], the reported LD_50_ value was higher than 5000 mg/kg for both aqueous and methanolic extracts of *C. lancifolius* leaves, with no evidence of mortality, thus corroborating our findings. Typically, acute toxicity outcomes have limited clinical value. Therefore, a subacute toxicity investigation was also performed. 

### 4.3. Repeated-Dose Subacute Toxicity

Repeated-dose toxicity evaluation is useful in examining the effects of plant extracts on target organs and biochemical or hematological parameters, as these effects are typically not detectable during single-dose testing. It is necessary as well for ensuring human safety, particularly in the preparation of pharmaceuticals [81]. In this work, therefore, the subacute toxicity profile of the *C. lancifolius* leaf aqueous extract was observed in mice by measuring body weights and biochemical, hematological and histological parameters. Repeated-dose intake of CLAE produced no mortality and behavioral alterations but caused an insignificant reduction in the body weight and organ weight of extract-treated animals relative to the control group. As a consequence of repeated consumption, changes in body weight and organ weight are early markers of toxicity induced by the administered substance [82]. However, numerous pieces of evidence confirmed that a body weight decrease is accompanied by adaptive physiological responses to the given extract rather than the harmful effects of plant chemicals, which lower the desire for appetite and, as a result, reduce the calorie intake of the animals [83]. Therefore, at both tested doses, the non-significant (*p* > 0.05) variations in the final body weight and in the weights of the heart, kidney and liver may not be indicative of the extract’s deleterious potential against these organs. 

An evaluation of hematological parameters allows the researchers to detect the extent of detrimental effects of foreign chemicals, such as plant components, on blood. Both CLAE-administered groups examined a slightly insignificant (*p* > 0.05) increase in the WBCs, lymphocytes and neutrophils after the subacute administration of the extract. These outcomes indicate that CLAE may contain phytochemicals that augment the immune response by boosting the number of WBCs [84]. RBC, Hb, hematocrit, MCV, MCH and MCHC levels did not alter significantly, suggesting that the extract does not contain toxic chemicals that can cause anemia or other problems. A normal elevation in the platelet count is clear evidence that CLAE might prevent thrombocytopenia while supporting the body’s defense mechanisms [83].

The estimation of biochemical parameters is very critical when examining the toxic effects of plant materials because these characteristics may provide substantial information on certain organs—mainly the heart, kidney and liver—which are essential for an organism’s existence [85]. Three liver enzymes (ALP, ALT and AST), bilirubin, albumin and total proteins are the main clinical biochemistry indicators linked with liver malfunction. Enzymes are responsible for describing the cellular integrity of the liver, while bilirubin, albumin and total proteins specify their functionality [81]. The biochemical analysis disclosed that the serum level of these parameters was slightly higher in mice treated with 400 and 800 mg/kg of CLAE in comparison with control group animals, but this was not significant. Abnormal elevations of ALP, ALT, AST, bilirubin, albumin and total proteins are related to liver injury or other diseases [84]. Another fact of this study is the non-significant rise in the levels of total cholesterol and triglycerides, which suggests that CLAE may not have adverse effects on the cholesterol metabolism of mice. Serum electrolytes (sodium, potassium and chloride), creatinine and uric acid levels can be measured to check renal function, and a significant enhancement in any of these markers leads to disease and dysfunction of the kidneys’ filtration system [86]. In this work, the average level of these parameters was found to be slightly higher in mice at both concentrations of CLAE, but it remained within the normal ranges. Based on these consequences, it appears that CLAE has no negative effects on the kidney function of mice. 

According to the hematological and biochemical findings of our research, it was rational to postulate that repeated-dose consumption of CLAE may possess toxic effects on vital organs, and this evidence was further supported by histopathological studies. The photomicrographs of kidney and liver sections of mice administered with 400 and 800 mg/kg of CLAE showed some histological variations such as degenerated epithelial cells around the tubules, existence of cells in the renal tubular lumen and infiltration of inflammatory cells. This CLAE toxicity could have developed from the adverse effects of a variety of active phytochemical constituents present in CLAE as seen from the LC-ESI-MS/MS results. The nonappearance of histological changes in the architecture of the heart depicts that CLAE may not cause cardiotoxicity. Therefore, the histopathological outcomes are consistent with the biochemical findings; thus, a long-term chronic study (90 days) is proposed for the comprehensive understanding of the hepatotoxic and nephrotoxic mechanism of this plant. 

### 4.4. ISO-Induced Chronic Myocardial Injury

Myocardial infarction (MI) is one of the common causes of abnormal heart structure and function [87]. Isoprenaline (ISO), a β-adrenergic agonist, is used as an inducer in the development of different animal models for the appraisal of cardioprotective properties of various agents, since, at high concentrations, ISO causes hypoxia, necrosis and other changes at a pathological level during MI [88]. The main purpose of this study was to determine whether CLAE has the ability to have a therapeutic effect on cardiac remodeling in experimental rats following ISO-induced chronic MI. The CLAE has the tendency to restore ISO abnormalities and showed protection against myocardium tissue damage. From a histopathological perspective, in both treatment categories, CLAE-treated rats displayed less inflammatory cell infiltration, minimum evidence of necrotic myocardial fibers and interstitial edema as compared to ISO-alone treated rats. In light of these findings, it appears that CLAE may effectively lessen ISO-induced myocardial injury. 

Conferring the heart weight index, it was significantly raised in the ISO-alone treated group in comparison with the control group and is discussed in terms of cardiac hypertrophy (CH) [89]. The ISO-induced hypertrophic response is related to this rise in the heart weight index [90]. Since substantial necrosis of heart muscles occurs after exposure to ISO, the observed rise in heart weight may be due to an increase in the permeability of the plasma membrane, water content, infiltration of inflammatory cells, protein synthesis and intramuscular edema [91]. Excessive OS as well as higher glucose uptake in the myocardium may also account for the increased heart weight as seen in ISO-treated rats [92]. Our study exhibited that CLAE attenuates ISO-induced CH with a significant decrease in the heart weight index at both treatment doses relative to the ISO-administered rats, which is in agreement with previous investigations [89,93]. 

Measuring serum levels of certain diagnostic markers including AST, ALT, CPK, CK-MB, cTnI and LDH is very useful to authenticate the degree of myocardial injury protection [94]. The consequences of our study explored that pretreatment of CLAE reduced the pathogenic elevations of these cardiotoxicity indicators in experimental rats in comparison with ISO-alone treated rats. The beneficial effects of CLAE were probably due to the enhanced integrity of the cardiac membrane, thereby diminishing the amount of these enzymes released into the bloodstream [93]. Recently, mounting evidence confirms that inflammation is a key process engaged in cardiac tissue damage following ISO administration. In the infracted region, various inflammatory cells infiltrate with the countless release of inflammatory chemokines and cytokines followed by excessive OS [95,96]. The up-regulated inflammatory cytokinin IL-6 in the ISO-alone treated rats was markedly reversed by the CLAE in our study. Previously, Chen et al. [97] examined the cardioprotective effect of pinoresinol diglucoside (PDG) in male SD rats via the downregulation of inflammatory cytokines, i.e., IL-6, IL-1β and TNF-α. NO is a principal contributor to the normal functioning of the vascular endothelium. The process of vasomotion as well as blood pressure is regulated by the endogenous generation of NO [98]. Under physiological stress conditions, a reversible post-translational modification occurs in the NO molecule via its covalent attachment with thiol protein to form S-nitrosothiol, thereby hindering their further oxidative adaptations by ROS and thus averting cardioprotection [99]. Here, we studied a prominent increase in the plasma NO levels in CLAE + ISO groups when compared with the ISO-alone control group, thus supporting the earlier findings [41,91,100]. According to Eyup et al. [101], a foremost risk factor for MI is lipids buildup in the blood. 

Experimental evidence has shown that both LDL and HDL share opposing associations with MI risks [102]. The increased serum levels of LDL, TC and TG followed by low HDL levels in ISO-induced rats were restored significantly in CLAE-treated groups, indicating the putative antilipidemic activity of CLAE through the maintenance of the lipid parameters and lipoproteins in myocardial injury rats. This function of CLAE to lower cholesterol levels may have resulted from its HMG-CoA reductase inhibitory potential, a rate-controlling enzyme in the metabolic pathway of cholesterol biosynthesis [103]. In this way, CLAE may facilitate cholesterol transportation to the liver for its breakdown and excretion. Our study outcomes are in agreement with the verdicts of Shaito et al. [104] and Hu et al. [105], who listed the role of biologically active herbal constituents in the prevention of dyslipidemia and MI. 

Earlier studies implied that the pathogenesis of ISO-induced myocardial injury could be significantly influenced by the maladaptation in oxidative stress mediated by ROS (reactive oxygen species) [106]. The mitochondria are the major site of synthesis for myocardial ATP (adenosine triphosphate) and these ROS. Oxidative-stress-induced mitochondrial damage provides the basis for the loss in integrity and function of mitochondrial membranes, resulting in lipid, protein and DNA damage and thus leading to the activation of cardiomyocytes apoptosis and dysfunction, which, in turn, promote the release and activation of the cytochrome C from mitochondria. In this way, apoptosis lowers the contraction of heart muscles, which ultimately leads to MI [107]. In addition, CH was found to be associated with the activation of different extracellular stimuli by ROS. These stimuli, which include angiotensin-II, TNF-α and endothelin-1 (ET-1), could mediate CH by interrupting multiple downstream signaling processes (mitogen-activated protein kinases (MAPKs), calcineurin, NF-κB, protein kinase C (PKC) and tyrosine kinases) [106]. Moreover, ROS generation in myocardial tissues after MI has several other mechanisms. Initially, xanthine oxidoreductase (XOR) has been indicated as a major source of ROS production on reperfusion injury sites [108]. Different experiments also proposed that nicotinamide adenine dinucleotide phosphate oxidase 2 (Nox2) expression in cardiomyocytes is increased after MI [109]. There are some other mechanistic approaches that trigger ROS production after MI, which warrants further action. 

Several research groups have attempted to determine the possible noticeable role of antioxidants in different cardiac disease states by suppressing ROS generation. Different hypothetical underlying mechanisms of antioxidants were examined in CVDs. Polyphenolic compounds, especially flavonoids, have very powerful antioxidant and anti-inflammatory activities [76]. For example, isoorientin, a flavone C-glycoside, regulated the ROS level and reduced doxorubicin-induced myocardial ischemic injury through the modulation of MAPK, Akt (protein kinase B) and caspase-dependent signaling pathways [110]. Silymarin and its constituents (silibinin A and B and isosilybin A and B) showed a variety of in vitro and in vivo pharmacological actions including antioxidant, anti-inflammatory, cardioprotective and hepatoprotective activity [111]. Taleb et al. [112] reported the antioxidant potential of silymarin in OS-induced cardiovascular ailments. A recent study examined that isosilybin activated fatty acid oxidation, thereby inhibiting lipid synthesis via the AMPK (adenosine monophosphate-activated protein kinase) signaling pathway [113]. Together with flavonoids, the cardioprotective potential of a phenolic acid, vanillic acid, in acute cardiac hypoxia/reoxygenation injury was associated with the suppression of OS through AMPK*α*2 protein downregulation [114]. Stanely Mainzen Prince et al. [115] linked the protective effects of vanillic acid in ISO-induced cardiotoxic rats with its remarkable antiradical and anti-inflammatory properties. It also revealed anti-hypertensive activity in monocrotaline-induced pulmonary arterial hypertension by stimulating NO signaling pathways [116]. Therefore, by considering the cardioprotective potential of CLAE, it is concluded that the protective effect of CLAE in ISO-induced myocardial infracted rats is due to its OS-suppressing ability, which is directly or indirectly related to the antioxidant properties of its phytoconstituents. The co-expression of drug-likeness, ADMET, molecular docking and network analysis also confirmed that among the CLAE phytoconstituents detected through LC-ESI-MS/MS, isoorientin, isosilybin A and vanillic acid act efficiently against the key proteins and target multiple signaling pathways to offer protection against chronic MI. An acceptable range of drug-likeness and ADMET parameters was unveiled by most of the CLAE phytochemicals, which reflect their capacity for the development of promising drug candidates. Isoorientin followed by isosilybin A, epigallocatechin and fustin had strong interactions with HMG-CoA reductase with docking scores ranging from −6.849 to −8.389 kcal/mol, as compared to an FDA-approved drug, verapamil (docking score: −3.182 kcal/mol), whereas vanillic acid (docking score: −6.534 kcal/mol) exposed better docking association towards the NOS protein than the reference inhibitor verapamil (docking score: −3.539 kcal/mol). Based on network pharmacology analysis, the IL-17 signaling pathway, lipid and atherosclerosis, TNF signaling pathways, complement and coagulation cascades and fluid shear stress and atherosclerosis might be the major KEGG pathways with the most prominent connections to the cardiac target genes and CLAE molecules for treating chronic MI after filtering the few channels that are not directly connected to chronic MI. 

Hence, in the current study, the in vitro, in vivo and computational outcomes prescribed the antioxidative and cardioprotective role of CLAE by targeting multiple signaling processes. Our work lays an experimental and theoretical foundation for the discovery and development of new cardioprotective agents in the future through further research.

### 4.5. Behavioral Studies

Noise-induced stress is a leading contributor to neurological disorders. It is strongly linked with neurobehavioral discrepancies and plays a key role in the genesis of anxiety, depression and cognitive impairment [20]. Previous studies revealed that children who are subjected to noise repeatedly are more likely to develop behavioral problems, such as an increased risk of acquiring social anxiety and low confidence [117]. Acute and chronic NS causes cellular damage and progressive degeneration in hippocampus functioning due to the excessive glucocorticoids release from the hyperactivation of the hypothalamic–pituitary–adrenal axis (HPA), which plays a crucial role in fear and hopelessness situations [118]. In addition, exposure to acute NS elevates the AChE activity in a compensatory way in the hippocampus, which is attributed to anxiety, depression-like behaviors and memory deficits in stressed animals [4,119,120]. It was stated previously that ethanolic and water extracts of *C. lancifolius* leaves displayed substantial AChE inhibitory potential [29]. In the present study, we verified for the very first time that CLAE has moderate AChE inhibition. 

Common symptoms of anxiety include less exploration and lack of social contact [121], which are in line with the outcomes of our study. We observed the anxiolytic effect of CLAE via OFT and EPM tests and found that animals induced with NS spent less time in the central zone and open arms accompanied by a decreased number of entries in particular zones, i.e., center area of the open field arena and elevated open arms in OFT and EPM test, respectively, as compared to the animals in all extract-treated categories. Similarly, enhanced periods of immobility in noise-stressed animals respective to unstressed and CLAE-treated animals during FST specify their depressive behavior. Cognitive deterioration is a common consequence of anxious and depression-like behaviors [122]. According to recent studies, acute NS has a deleterious impact on memory [15] and is justified by our work as well because the NS-induced rats had poor memory of familiar objects and the platform zone in NOR and MWM tests, respectively, when compared with the control group and CLAE-treated rats. Our research is the first preliminary report on the inhibitory action of CLAE on NS-induced anxiety, depression and memory impairment in rats. 

The particular underlying mechanisms behind NS-induced anxiety, depression and cognitive disturbance need more attention; however, numerous overwhelming pieces of evidence suggested that OS is one of the common etiological factors in the pathogenesis of these brain ailments [4,123]. The overburden of ROS in the neuronal microenvironment can accelerate the mutability of lipid membranes, proteins and nucleic acids, thereby resulting in the disruption of normal cellular homeostasis [124,125]. Moreover, this oxidant and antioxidant imbalance also infects normal AChE activity in the cholinergic system [126,127]. Hence, therapeutic approaches strengthening the antioxidant enzyme potential, including catalase (CAT), glutathione peroxidase (GPx) and superoxide dismutase (SOD), reducing the malondialdehyde (MDA) level (main end-product of lipid peroxidation), scavenging the other free oxygen species or regularizing the AChE function in different brain areas may have a precautionary effect against NS-induced anxiety, depression and cognitive failure [8,127]. Antioxidants are substances that can scavenge excessively produced ROS and therefore mitigate the OS burden [29]. Recent experimental approaches have shown that phenolics, particularly flavonoids, alkaloids, tannins, coumarins and lignans, are among the most effective antiradicals. Their ROS scavenging ability depends upon the distribution of hydroxyl groups as well as the phenol concentration [128]. 

It has been demonstrated that these polyphenolic chemicals prevent neurons from a wide range of neurodegenerative diseases, including anxiety, depression and memory deficits [129]. Likewise, by virtue of its antioxidant capability, an alkaloid principle vincamine from the lesser periwinkle plant, is also known for its neuroprotective attributes [130,131]. Therefore, the improvement in the anxious, depressive behavior and cognitive disabilities of NS-induced rats in the current study could possibly be ascribed to the phytochemicals of CLAE identified in the LC-ESI-MS/MS investigation, which is further corroborated by ADMET tests, molecular docking and network analysis. For instance, isoorientin restores normal cognitive performance in experimental animals by lowering numerous unique pathological hallmarks of AD such as amyloid β protein (Aβ) deposition, tau hyperphosphorylation and neuroinflammatory responses [132]. Another study highlighted its amelioration role in scopolamine-induced impaired memory by restoring the cholinergic system, antioxidant enzyme balance and phosphorylated CREB/BDNF (brain-derived neurotrophic factor) pathway, thereby displaying memory-enhancing properties [133]. 

Several in vivo studies appraised the anxiolytic, antidepressant and memory-improving attribute of ellagic acid due to its antiradical and iron chelation action, activation of multiple cell-signaling pathways (normalization of lipidemic profile, regulation of proinflammatory markers such as IL-6, IL-1β and TNF-α and inhibitory effect on nuclear factor-κB (NF-κB) action) and lowering of mitochondrial dysfunction [134]. Different findings suggest neuropharmacological protective effects of quercetin in anxiety, Alzheimer’s disease, depression, amyloid β peptide, amyotrophic lateral sclerosis, Huntington’s disease and Parkinson’s disease. The Food and Drug Administration (FDA) of the United States approved the daily intake dose of quercetin up to 1.5 g/day [135]. Similarly, scopoletin alleviates anxious behaviors in Freund’s adjuvant-induced mouse model by inhibiting NF-κB and MAPK (mitogen-activated protein kinase) signaling pathways [136]. Its effectiveness in intracerebral hemorrhage-induced brain injury is also promising [137]. On the basis of its direct nAChR (nicotinic acetylcholine receptor) agonistic property, scopoletin has memory-enhancement effects [138]. 

Vasicine, a quinazoline alkaloid from *Adhatoda vasica*, showed potent inhibition of the AChE enzyme [139,140]. On the other hand, the drug-likeness and ADMET profiles of these phytoconstituents were also very reasonable with few shortcomings that can be addressed in the drug development process [57]. The post-docking analysis also confirmed that 12 top-docked CLAE chemicals, such as isoorientin, epigallocatechin, gancaonin E, ellagic acid, scaposin, isosilybin A, quercetin, 14,15-dehydro-16-epi-vincamine, enterolactone, scopoletin, biochanin A and vasicine, were found to have strong binding affinities towards target protein *h*AChE with docking score varying from −7.848 to −15.180 kcal/mol, which was extremely comparable with the FDA-approved drug galantamine (docking score: −9.742 kcal/mol). Network pharmacology investigations explored that they would have an influence on neurological disorders by interacting with KEGG pathways including neuroactive ligand–receptor interaction, calcium signaling pathway, Alzheimer’s disease, dopaminergic synapse and pathways of neurodegeneration. 

Neurotransmitter receptors are chief components of the neuroactive ligand–receptor interaction pathway, which plays a pivotal role in the pathophysiology of several brain disorders. The role of the calcium signaling pathway has also been implicated in different mental conditions, and the disruption of the Ca^2+^-CaM-NO/sGC (calcium-calmodulin-nitric oxide-activated guanylyl cyclase pathway) signaling cascade could result in the neuroprotective effects [141]. In this way, CLAE may exert its anxiolytic, antidepressant and memory-enhancing effect by targeting multiple underlying mechanisms. Thus, it is projected that phytoconstituents present in CLAE have the strength to diminish the anxiety and depression-like states and memory deficits in noise-stressed rats, but detailed studies will be recommended in this regard.

Overall, major groups of phytochemicals including alkaloids, coumarins, flavonoids, phenolic acids, tannins, terpenoids, lignans and carboxylic acids were predicted through the LC-ESI-MS/MS analysis of CLAE in negative and positive modes of analysis, whereas previous phytochemical reports on *C. lancifolius* established comparable outcomes [11,21,22,23,29,142]. Due to these significant biologically active components, the *C. lancifolius* leaves may have antioxidant, cardioprotective, anxiolytic, antidepressant and memory-enhancing potential.

## 5. Conclusions

In conclusion, the findings of the current study established that the phytochemical profiling of *C. lancifolius* leaf aqueous extract through LC-ESI-MS/MS analysis displayed the presence of major groups of phytochemicals that could be responsible for the prominent antioxidant, cardioprotective, anxiolytic, antidepressant and memory-enhancing potential of CLAE as inferred from the different in vitro and in vivo experiments. Furthermore, in spite of a few histopathological alterations in the normal architecture of the kidney and liver in the subacute toxicology investigation, no mortality or any signs of clinical toxicity were reported in both single-dose and repeated-dose toxicity examinations. In addition, the drug-likeness and ADMET prediction studies confirmed that the majority of the examined compounds have acceptable properties to pass Lipinski’s criteria and other physicochemical and pharmacokinetic filters. Likewise, the molecular docking experimentation demonstrated that phytochemicals of CLAE had higher binding affinities with the disease target proteins. The network pharmacology analysis exhibited that the various bioactive compounds of CLAE reflected complex relationships with potential target genes and KEGG signaling channels associated with chronic MI, anxiety, depression and impaired memory. Finally, this precious piece of information provides a solid mechanistic foundation for further investigations as well as for the clinical use of CLAE in chronic MI, anxiety, depression and memory and learning deficits. 

## Figures and Tables

**Table 1:** Total phenolic content (TPC), total flavonoid content (TFC), antioxidant (DPPH, TAC and TRP) and AChE inhibitory capacity of CLAE.

Sample	TPC	TFC	DPPH Assay	TAC	TRP	AChE Assay
	(µg GAE/mg)	(µg QE/mg)	IC_50_ (µg/mL)	(µg AAE/mg)	(µg GAE/mg)	IC_50_ (µg/mL)
				250 µg/mL	250 µg/mL	
CLAE	67.70 ± 0.15	47.54 ± 0.45	16.66 ± 0.42	77.33 ± 0.41	79.11 ± 0.67	110.13 ± 1.71
	-	-	8.67 ± 1.83 ^a^	-	-	6.48 ± 1.29 ^b^

^a^ Ascorbic acid was used as standard in DPPH assay; ^b^ Galantamine was used as positive control in AChE inhibitory assay; TPC: Total phenolic content; TFC: Total flavonoid content; DPPH: 2,2-Diphenyl-1-picrylhydrazyl; TAC: Total antioxidant capacity; TRP: Total reducing power; AChE: Acetylcholinesterase; GAE: Gallic acid equivalent; QE: Quercetin equivalent; AAE: Ascorbic acid equivalent. Results were stated as mean ± SD (*n* = 3).

**Table 2:** Effect of single-dose and repeated-dose administration of CLAE on treated mice body and organ weight.

Parameters	Control	Single-Dose Acute	Repeated-Dose Subacute	
Observed		Toxicity	Toxicity	
		2000 mg/kg	400 mg/kg	800 mg/kg
Final body weight (g)	33.84 ± 2.68	34.76 ± 3.43	32.42 ± 1.73	30.41 ± 3.20
Organ weight				
Heart (g)	0.269 ± 0.021	0.272 ± 0.015	0.261 ± 0.007	0.265 ± 0.030
Kidney (g)	0.342 ± 0.039	0.354 ± 0.023	0.341 ± 0.037	0.339 ± 0.015
Liver (g)	2.844 ± 0.224	2.821 ± 0.269	2.711 ± 0.192	2.674 ± 0.167

Values are expressed as mean ± SD. Data was considered significant when *p* < 0.05 (One-way ANOVA followed by Dunnett’s multiple comparison test).

**Table 3:** Serum biochemistry and hematological parameters of mice treated with CLAE for 28 days.

**Parameters**	**Control**	**Repeated-Dose Subacute Toxicity** **CLAE**	
		**400 mg/kg**	**800 mg/kg**
**Biochemicals**			
Albumin (g/dL)	1.732 ± 0.959	2.128 ± 1.326	2.792 ± 0.755
Albumin/Globulin ratio	2.112 ± 0.568	2.226 ± 0.347	2.718 ± 0.283
Total Protein (g/dL)	3.852 ± 1.927	4.412 ± 1.103	5.458 ± 0.362
Alkaline Phosphatase (U/L)	287.5 ± 18.97	303.6 ± 28.75	334.4 ± 41.91
Alanine Transaminase (U/L)	30.67 ± 8.491	39.36 ± 7.826	44.05 ± 9.680
Aspartate Transaminase (U/L)	98.76 ± 28.39	123.3 ± 19.19	141.2 ± 24.03
Uric Acid (mg/dL)	0.800 ± 0.100	1.028 ± 0.279	1.054 ± 0.365
Creatinine (mg/dL)	0.976 ± 0.392	1.288 ± 0.352	1.724 ± 0.630
Total bilirubin (mg/dL)	0.216 ± 0.055	0.232 ± 0.049	0.284 ± 0.102
Total Cholesterol (mg/dL)	79.80 ± 15.40	80.26 ± 17.91	83.41 ± 11.82
Triglycerides (mg/dL)	122.3 ± 27.46	132.4 ± 11.85	162.2 ± 39.85
Sodium (mmol/L)	110.7 ± 33.13	104.5 ± 23.16	151.2 ± 25.31
Potassium (mmol/L)	6.380 ± 1.108	7.176 ± 0.367	9.082 ± 0.128
Chloride (mmol/L)	96.00 ± 11.77	113.3 ± 20.01	127 ± 26.93
**Hematological**			
RBCs (10^6^/µL)	8.660 ± 2.195	9.640 ± 0.555	11.32 ± 0.978
Hemoglobin (g/dL)	13.24 ± 1.414	15.40 ± 1.517	20.38 ± 5.364
Hematocrit (%)	45.69 ± 2.355	48.26 ± 6.017	57.00 ± 10
Mean corpuscular volume (f/L)	54.20 ± 10.50	59.83 ± 12.79	71.19 ± 18.36
Mean corpuscular hemoglobin (pg)	17.80 ± 1.834	18.58 ± 2.924	22.61 ± 4.479
MCHC (%)	31.17 ± 6.058	32 ± 3.245	35.82 ± 2.305
WBCs (10^5^/µL)	4.643 ± 0.750	5.622 ± 0.809	5.671 ± 0.398
Neutrophils (%)	38.58 ± 3.846	42.80 ± 8.956	47.22 ± 5.516
Lymphocytes (%)	49.62 ± 6.243	52.34 ± 3.553	60.95 ± 10.71
Eosinophils (%)	1.060 ± 0.357	0.998 ± 0.416	1.080 ± 0.266
Platelets (10^5^/µL)	5.560 ± 0.931	6.977 ± 0.584	7.368 ± 1.227

Results are expressed as mean ± SD. Data were analyzed by using one-way ANOVA followed by Dunnett’s multiple comparison test when compared to the control and *p* < 0.05 was considered significant.

**Table 4:** Identification of chemical compounds from *C. lancifolius* leaf aqueous extract by using LC-ESI-MS/MS analysis.

Comp. No.	R.T. (min)	Analysis Mode	*m*/*z*	Product Ions (*m*/*z*)	Accurate Mass	Chemical Class	Proposed Compounds	References
1	0.45	[M − H]^−^	130.67	129, 113.92, 101.08, 99, 85,	131.09463	Amino acid	L-Leucine	MassBank
				83.08, 69				KO001262
2	0.59	[M − H]^−^	133	115, 105, 97, 89, 71, 59.08	134.02152	Carboxylic	Malic acid	MassBank
						acid		KO001305
3	0.74	[M − H]^−^	166.92	152, 139.08, 131, 123, 108,	168.04230	Phenolic	Vanillic acid	MassBank
				92.92, 73.08, 59.17		acid		ML005851
4	0.81	[M − H]^−^	169	155.67, 141.08, 131.08, 125,	170.02152	Phenolic	Gallic acid	MassBank
				113, 92.83, 81.08, 68.75		acid		KO000889
5	1.73	[M − H]^−^	196.75	182.08, 169.08, 161, 152.92,	198.05282	Phenolic	Syringic acid	MassBank
				124.92, 109, 96.92		acid		KO001814
6	2.45	[M − H]^−^	207.08	192, 171, 169, 163, 149.08,	208.213	Phenolic	3,4-Dimethoxy-	MassBank
				132.92, 121, 89.08, 71.25		acid	cinnamic acid	PR306017
7	3.19	[M − H]^−^	275	260.08, 257, 239.08, 229.08,	276.09977	Coumarin	Capensine	MassBank
				201, 165.08, 147.17, 113				BML00229
8	3.25	[M − H]^−^	287.08	268.92, 253.83, 250.92, 249,	288.06339	Flavonoid	Fustin	MassBank
				198.92, 154.92, 135, 126.83				BS003856
9	3.74	[M − H]^−^	297.17	281.08, 279.17, 253.25,	298.33890	Lignan	Enterolactone	MassBank
				209.25, 197.08, 183, 153.25				PR308012
10	3.83	[M − H]^−^	301.03	284, 257.08, 229, 201, 125,	302.04265	Flavonoid	Quercetin	MassBank
				117				PB002411
11	3.89	[M − H]^−^	305	273.17, 244.83, 230.50,	306.07395	Catechin	Epigallocatechin	MassBank
				225.08, 207.17, 181.08, 103				BS003952
12	4.19	[M − H]^−^	349.08	331, 313, 269.33, 241,	350.20932	Diterpene	Andrographolide	MassBank
				150.92, 138.92, 124.75				BML80746
13	4.24	[M − H]^−^	351	333, 314.92, 306.92, 276.83,	352.434	Alkaloid	14,15-Dehydro-16-	MassBank
				224.92, 222.83, 169, 152.67			epi-vincamine	PR304643
14	4.55	[M − H]^−^	380.75	362, 299.92, 255.83, 129.58,	381.17875	Alkaloid	Otosenine	MassBank
				126.92				BML81861
15	4.97	[M − H]^−^	383.17	365, 347, 339.25, 306.92,	384.476	Alkaloid	Voacristine	MassBank
				248.92, 221.92, 176.75				PR304465
16	5.24	[M − H]^−^	385	367, 348.92, 327.25, 287.17,	386.353	Glycoside	1-*O*-	MassBank
				237.08, 190.92, 154.83			Sinapoylglucose	PR309003
17	5.35	[M − H]^−^	389.25	370.83, 352.92, 350.92,	390.0951	Flavonoid	Scaposin	MassBank
				345.17, 315, 271, 241, 153				BS003673
18	5.79	[M − H]^−^	417.33	398.92, 380.92, 373.08,	418.354	Flavonoid	Kaempferol-3-*O*-	MassBank
				318.83, 267.08, 241, 160.75		glycoside	arabinoside	PR306890
19	5.94	[M − H]^−^	421.25	403.08, 384.92, 382.92,	422.17293	Flavonoid	Isoangustone A	HMDB
				346.92, 327.17, 293.08				HMDB0038905
20	6.08	[M − H]^−^	423.08	405, 386.92, 349.08, 343.08,	424.18858	Flavonoid	Gancaonin E	HMDB
				328.17, 302.83, 279, 222.67				HMDB0038868
21	6.44	[M − H]^−^	433.42	396.92, 365.08, 355.92, 317,	434.397	Flavonoid	Naringenin-7-*O*-	MassBank
				269.42, 214.08, 182.08			glucoside	PR309316
22	6.53	[M − H]^−^	447.17	428.92, 411, 372.83, 349,	448.10056	Flavonoid	Isoorientin	MassBank
				301, 296.92, 201.17, 182				FIO00716
23	6.94	[M − H]^−^	481	463, 444.83, 442.83, 423,	482.12137	Flavonoid	Isosilybin A	MassBank
				392.92, 351.25, 331, 301				FIO01031
24	7.66	[M − H]^−^	529.92	511, 493.83, 469.25, 453.33,	531.24683	Alkaloid	Harringtonine	MassBank
				449.08, 431.33, 403.25,				BML82381
				325.08, 299.92, 231.92, 182				
25	0.40	[M + H]^+^	116.08	107, 98, 84, 74.08, 70, 58.08	115.06333	Amino acid	L-Proline	MassBank
								KO003672
26	0.66	[M + H]^+^	123	106.33, 105.08, 94.83, 81.83,	122.03677	Carboxylic	Benzoic acid	HMDB
				67.67, 52.08		acid		HMDB0001870
27	0.81	[M + H]^+^	130.25	114.17, 112.08, 98.08, 88.08,	129.07897	Amino acid	Cycloleucine	HMDB
				74.08, 70.08, 57				HMDB0062225
28	1.00	[M + H]^+^	138	133.50, 121, 118.08, 96.83,	137.04768	Benzoic acid	4-Aminobenzoic	MassBank
				94, 78.92, 70, 57		derivative	acid	KO002120
29	1.31	[M + H]^+^	148	139.08, 130.08, 116, 112.08,	147.05316	Amino acid	Glutamic acid	MassBank
				102.08, 100, 88.08, 79, 61				PB000463
30	1.63	[M + H]^+^	149	140, 131.08, 121, 111, 93, 79,	148.161	Cinnamaldehyde	*p*-Coumaraldehyde	MassBank
				58.83				PR304040
31	1.80	[M + H]^+^	156.83	142, 140.17, 134.92, 129.08,	156.06874	3-Alkylindoles	3-Indoleacetonitrile	HMDB
				111.08, 83.92, 79, 58.67				HMDB0006524
32	1.89	[M + H]^+^	163	145.08, 135, 133, 121.08,	162.0317	Coumarin	4-	MassBank
				107, 95.08, 79, 70, 61		derivative	Hydroxycoumarin	NA000184
33	2.12	[M + H]^+^	175.08	157.08, 153.08, 130.08, 116,	174.01643	Carboxylic	*cis*-Aconitic acid	HMDB
				112.08, 81.08, 72.08, 60.08		acid		HMDB0000072
34	2.51	[M + H]^+^	181.08	166.92, 163, 153.17, 149,	180.0423	Phenolic	Caffeic acid	MassBank
				135.08, 121, 112, 95, 69.08		acid		RP016701
35	2.65	[M + H]^+^	189.08	174.92, 171.08, 144.08,	188.23	Alkaloid	Vasicine	MassBank
				130.08, 114.92, 109, 95.08				PR301208
36	2.94	[M + H]^+^	193.08	184.08, 175.08, 168.92, 155,	192.04226	Coumarin	Scopoletin	MassBank
				148.92, 133, 120.08, 95.08				PB002203
37	3.17	[M + H]^+^	203.08	186.17, 185.08, 173.17, 161,	202.213	Alkaloid	Vasicinone	MassBank
				147, 143, 133.08, 103.08, 81				PR301376
38	3.40	[M + H]^+^	205.08	188.08, 177, 141	204.08988	Amino acid	Tryptophan	MassBank
								FIO00629
39	3.48	[M + H]^+^	215	200.08, 186.83, 174, 154.92,	214.11061	Alkaloid	Harmaline	MassBank
				131.08, 114, 110.08, 70.08				KO003099
40	3.91	[M + H]^+^	221.08	212.92, 203.08, 179, 177.08,	220.07355	Coumarin	5,7-Dimethoxy-4-	MassBank
				161.08, 157, 121.17, 68.83			methylcoumarin	BML01472
41	4.14	[M + H]^+^	229.25	226.75, 211.17, 199.08, 193,	228.247	Stilbene	3,4,5-	MassBank
				190.92, 169, 142.08, 86.17			Trihydroxystilbene	PR302602
42	4.87	[M + H]^+^	255.08	246.67, 237.08, 235, 227.17,	254.0579	Flavonoid	Chrysin	MassBank
				190.58, 183.08, 171, 101, 79				LU080404
43	5.39	[M + H]^+^	275.08	257.25, 247.08, 231.17, 197,	274.0841	Naphthopyra-	*(R)*-Semivioxanthin	MassBank
				170, 159.08, 102.08, 88.08		none		HB003729
44	5.58	[M + H]^+^	285	267.08, 249.17, 229.08,	284.0685	Flavonoid	Biochanin A	MassBank
				225.08, 206.92, 188.58, 173,				LU096003
45	5.74	[M + H]^+^	303.08	285.17, 275.08, 247, 229.08,	302.1991	Tannin	Ellagic acid	MassBank
				199, 174.92, 137.17, 102.17				CB000408
46	5.91	[M + H]^+^	305.17	296.25, 268.75, 248.58, 245,	304.2587	Flavonoid	Taxifolin	MassBank
				192.50, 166.92, 135.33, 96				CB000303
47	6.91	[M + H]^+^	365.33	333.25, 308.83, 275.08,	364.09468	Lignan	Justicidin B	MassBank
				261.25, 184.42, 151.25				BML01604
48	8.71	[M + H]^+^	441.33	423.25, 418.08, 410.75, 399,	440.162	Lignan	Maruchantin E	MassBank
				381.08, 366.58, 339.25,				PB011901
				313.17, 310.50, 250.75,				
49	9.38	[M + H]^+^	481.42	472.42, 449.25, 424.83, 393,	480.29881	Alkaloid	Emetine	MassBank
				408.83, 383.25, 367.08, 261				CE000076
50	10.04	[M + H]^+^	529.50	492.17, 483.08, 473.42,	528.38148	Triterpenoid	Pachymic acid	MassBank
				468.92, 464.92, 424.25, 417,				MSJ00299
51	12.30	[M + H]^+^	819.75	801.58, 783.75, 771.08,	818.687	Flavonoid	Flavonol base +	MassBank
				752.58, 744.08, 735.75,		glycoside	4O, 1MeO, O-Hex-	PR310938
				683, 668.33, 653.25, 632.58,			Hex, O-Hex	
52	13.31	[M + H]^+^	839.75	790.17, 691.42, 665.33,	838.941	Triterpenoid	Licoricesaponin G2	MassBank
				579.33, 526.42, 490.25,		saponin		PR310662
				466.92, 448.92, 378.17				
53	13.75	[M + H]^+^	867.21	849, 816, 701.42, 692.42,	866.20581	Tannin	Procyanidin C1	HMDB
				611.50, 517, 509, 494,				HMDB0038370
				477.25, 395.67, 284.08				

Comp. no.: Compound no.; R.T.: Retention time; HMDB: Human metabolome database; *m*/*z*: Mass to charge ratio.

**Table 5:** Binding energies (kcal/mol) of various phytochemicals with acetylcholinesterase, HMG-CoA reductase and nitric oxide synthase measured by Prime MM-GBSA.

Name	Docking Score (XP- Glide)	Glide Energy	ΔG Binding	Log K_i_ (µMolar)	ΔG Coulomb	ΔG Covalent	ΔG Hbond	ΔG Lipo	ΔG Solv GB	ΔG vdW	Interactions of Residues and Their Ligands with Distance (Å)	
											Hydrogen Bonds	Electrostatic/ Hydrophobic Bonds
***HMG-CoA reductase (PBD ID: 2R4F)***												
Isoorientin	−8.389	−63.875	−24.43	−7.38	−35.84	1.67	−6.02	−4.16	72.05	−49.95	**Conventional Hydrogen**	**Electrostatic π-Cation;**
											**Bond:** Lys633 (2.58),	**π-Donor Hydrogen**
											Lys633 (2.42), Glu700	**Bond:** Lys633 (3.01)
											(1.89), Glu700 (2.17),	**π-Anion:** Glu700 (4.49),
											Glu610 (2.10), Gln648	Glu700 (3.75), Glu700
											(1.95), Glu610 (2.04),	(3.47),
											Glu610 (2.00),	**π-Sigma:** His635 (2.90),
											**Carbon Hydrogen**	**π-Alkyl:** Lys633 (5.46),
											**Bond:** His635 (2.64)	
Isosilybin A	−7.992	−64.444	−29.2	−9.45	−41.77	12.23	−5.46	−6.67	61.31	−46.18	**Conventional Hydrogen**	**Electrostatic π-Cation:**
											**Bond:** Lys633 (2.72),	Lys633 (4.86),
											Lys633 (2.43), Thr636	**π-Anion:** Glu610 (4.75),
											(1.88), Lys606 (2.60),	**π-π Stacked:** His635
											Thr636 (2.35), Glu610	(4.40),
											(2.21), Gln632 (1.63),	**π-π T-shaped:** His635
											Thr636 (1.74),	(4.88),
											**Carbon Hydrogen**	**π-Alkyl:** Lys633 (4.98)
											**Bond:** His635 (2.64),	
											Lys633 (2.51), Glu700	
											(2.49), Leu634 (2.45),	
											Leu634 (2.43), Glu610	
											(2.54)	
Epigallocatechin	−6.897	−50.109	−20.35	−5.61	−42.43	9.39	−4.38	−2.78	50.9	−30.01	**Conventional Hydrogen**	**Electrostatic π-Cation:**
											**Bond:** Thr636 (2.08),	Lys633 (4.74),
											Thr636 (2.41), Leu634	**π-Anion:** Glu610 (4.97),
											(2.19), Glu700 (1.80),	**π-π T-shaped:** His635
											Gln632 (1.76), Lys633	(4.89),
											(2.09), Glu700 (2.04),	**π-Alkyl:** Ala585 (4.44),
											Glu700 (2.44),	Lys633 (4.84)
											**Carbon Hydrogen**	
											**Bond:** Glu610 (2.47)	
Fustin	−6.849	−42.993	−18.5	−4.81	−19.44	2.55	−3.67	−3.49	39.42	−32	**Conventional Hydrogen**	**Electrostatic π-Cation:**
											**Bond:** Lys633 (2.27),	Lys633 (4.79),
											Thr636 (1.93), Thr636	**π-Anion:** Glu700 (4.20)
											(2.31), Leu634 (1.77),	**π-π Stacked:** His635
											Gln648 (2.41), Thr636	(4.42)
											(1.98),	**π-π T-shaped:** His635
											**Carbon Hydrogen**	(5.64)
											**Bond:** His635 (2.47),	
											Glu700 (2.51)	
Verapamil	−3.182	−61.455	−14.78	−3.19	−2.4	−1.22	−3.16	−4.43	43.98	−46.89	**Conventional Hydrogen**	**Electrostatic Salt**
											**Bond:** Lys633 (2.20)	**Bridge; Attractive**
											**Carbon Hydrogen**	**Charge:** Glu700 (2.35),
											**Bond:** Lys633 (3.07),	**π-Cation** **;π-Donor**
											Gln648 (2.74), Glu700	**Hydrogen Bond:** Lys633
											(2.82), Gln648 (2.72),	(3.19),
											Gln648 (2.45), Leu634	**π-Cation:** Lys 606
											(2.58), Leu634 (2.95),	(4.09),
											Glu610 (2.66), Ile699	**π-Anion:** Glu610 (3.96),
											(2.60), Thr636 (2.66),	Glu700 (4.01),
											Ile699 (2.75), Glu700	**Alkyl:** Ala585 (4.35),
											(2.80), Gln648 (2.75),	**π-Alkyl:** His635 (4.66),
											Lys633 (2.82)	His635 (4.07), Ala585
												(4.58), Lys633 (4.49)
***Nitric oxide synthase (PBD ID: 1M9R)***												
Vanillic acid	−6.534	−25.711	−13.92	−2.82	−44.7	1.9	−2.83	−16.06	81	−27.64	**Conventional Hydrogen**	**π-π Stacked:** Trp445
											**Bond:** Ser102 (2.72),	(5.16), Trp447 (4.60),
											**Carbon Hydrogen**	Trp447 (3.81),
											**Bond:** Phe460 (2.68)	**Alkyl:** Val104 (5.13),
												**π-Alkyl:** Val104 (5.37),
												Ala446 (5.17)
Verapamil	−3.539	−41.256	−41.97	−15.00	17.01	0.52	−2.11	−12.09	−9.41	−34.32	**Conventional Hydrogen**	**Electrostatic Salt**
											**Bond:** Thr80 (2.31),	**Bridge;Attractive**
											Lys436 (2.40), Gln462	**Charge:** Glu432 (2.29),
											(2.15),	**Alkyl:** Leu431 (4.66),
											**Carbon Hydrogen**	**π-Alkyl:** Leu431 (5.13)
											**Bond:** Ser78 (2.72),	
											Ser78 (2.42), Ile79 (2.46),	
											Val71 (2.48), Ser78	
***Acetylcholinesterase (PBD ID: 4EY6)***												
Isoorientin	−15.180	−51.937	−67.31	−26.55	−50.13	6.57	−2.45	−17.67	43.31	−38.03	**Conventional Hydrogen**	**π-π Stacked:** Trp286
											**Bond:** Phe295 (1.87),	(5.42), Trp286 (5.17),
											Ser293 (1.74), Tyr337	Trp286 (4.02),
											(1.79),	**π-π T-shaped:** Tyr124
											**Carbon Hydrogen**	(5.49)
											**Bond:** Gly121 (2.50),	
											Val294 (2.85),	
											**π-Donor Hydrogen**	
											**Bond:** Lys633 (3.01),	
											Tyr124 (2.99), Trp286	
											(2.10)	
Epigallocatechin	−11.787	−42.294	−43.31	−15.58	−39	5.49	−3.61	−18.41	40.5	−23.62	**Conventional Hydrogen**	**π-π Stacked:** Trp286
											**Bond:** Phe295 (2.20),	(4.76),
											Tyr341 (1.61), Asp74	**π-π T-shaped:** Tyr72
											(1.86)	(5.01)
												**π-Alkyl:** Trp286 (5.17),
												Trp286 (3.82), Tyr341
												(5.24), Leu76 (5.44)
Gancaonin E	−11.706	−55.142	−31.99	−10.66	−16.94	7.62	−1.54	−28.71	64.06	−50.53	**Conventional Hydrogen**	**π-π Stacked:** Trp286
											**Bond:** Gly121 (2.95),	(5.55), Trp286 (4.29),
											Phe295 (2.45), Tyr341	**π-π T-shaped:** Tyr124
											(3.03)	(5.73), Phe338 (5.38),
												**π-Alkyl:** Trp86 (5.09),
												Trp86 (4.59), Trp86
												(4.99), Trp86 (3.83),
												Tyr124 (4.70)
Ellagic Acid	−11.403	−38.936	−68.58	−26.00	−32.35	4.93	−2.45	−20.64	24.98	−30.32	**Conventional Hydrogen**	**π-π Stacked:** Trp286
											**Bond:** Tyr72 (2.71),	(5.75), Trp286 (4.59),
											Phe295 (1.89), Ser293	Trp286 (4.61), Trp286
											(1.79), Tyr124 (1.92),	(4.09), Trp286 (3.74),
											Tyr337 (2.56),	Trp286 (3.76), Trp286
											**Carbon Hydrogen**	(4.64),
											**Bond:** Val294 (2.53)	**π-π T-shaped:** Tyr124
												(5.71)
Scaposin	−11.072	−47.758	−63.35	−24.28	−24.55	6.63	−1.75	−22.1	30.64	−43.95	**Conventional Hydrogen**	**π-π Stacked:** Trp286
											**Bond:** Phe295 (1.92),	(5.40), Trp286 (5.13),
											Tyr341 (2.40), Tyr337	Trp286 (4.01),
											(1.82),	**π-π T-shaped:** Tyr124
											**Carbon Hydrogen**	(5.43), Tyr337 (5.26),
											**Bond:** Gly121 (2.44),	**π-Alkyl:** Tyr72 (5.41),
											Val294 (2.87), Asp74	Trp286 (4.65), Trp286
											(2.68),	(3.96), Phe295 (5.19),
											**π-Donor Hydrogen**	Phe297 (4.21), Phe338
											**Bond:** Tyr124 (2.88)	(5.19), Tyr341 (4.65),
												His447 (5.24), His447
												(4.78)
Isosilybin A	−11.052	−55.882	−45.74	−16.64	−22.95	7.75	−3.01	−37.52	63.22	−44.57	**Conventional Hydrogen**	**π-π Stacked:** Trp286
											**Bond:** Phe295 (2.29),	(4.55), Trp286 (3.77),
											Ser293 (2.63), Glu202	**π-π T-shaped:** Tyr124
											(2.29),	(5.29), Tyr337 (5.34),
											**Carbon Hydrogen**	Tyr341 (4.72),
											**Bond:** Val294 (2.33),	**Amide-π Stacked:**
											Trp86 (2.79), Gly120	Gly120 (4.12), Gly121
											(2.93),	(4.12),
											**π-Donor Hydrogen**	**π-Alkyl:** Trp86 (5.25),
											**Bond:** Tyr124 (2.08)	Trp86 (5.37), Tyr124
												(5.48), Tyr337 (5.26),
												Phe338 (5.34),
Quercetin	−10.967	−49.91	−23.66	−6.61	−40.59	3.59	−3.17	−10.54	67.32	−29.99	**Conventional Hydrogen**	**Electrostatic π-Anion:**
											**Bond:** Tyr133 (2.38),	Asp74 (4.92),
											Ser125 (1.80), Glu202	**π-π Stacked:** Trp86
											(1.88), His447 (1.74),	(4.06), Trp86 (3.78),
											Tyr72 (1.81), Tyr72	Trp86 (4.48), Trp86
											(1.62),	(3.65),
											**Carbon Hydrogen**	**π-π T-shaped:** Tyr124
											**Bond:** Gly126 (2.77)	(5.23)
14,15-Dehydro-	−10.376	−34.243	−43.29	−15.57	−28.97	2.46	−0.78	−18.44	43.75	−35.9	**Conventional Hydrogen**	**Electrostatic π-Cation:**
16-epi-											**Bond:** Tyr341 (1.81),	Trp286 (4.69),
vincamine											**Carbon Hydrogen**	**π-π Stacked:** Trp286
											**Bond:** Tyr72 (3.10),	(5.03), Trp286 (4.68),
											Gln291 (2.74)	Trp286 (3.88),
												**Alkyl:** Leu289 (4.58)
Enterolactone	−9.057	−34.759	−49.58	−18.30	−27.93	7.71	−2.24	−30.84	34.56	−26.12	**Conventional Hydrogen**	**π-π Stacked:** Trp286
											**Bond:** Phe295 (1.78),	(4.80), Trp286 (4.77),
											Asp74 (1.92),	**π-π T-shaped:** Tyr72
											**Carbon Hydrogen**	(4.85), Tyr124 (5.09),
											**Bond:** Phe295 (2.64),	Tyr337 (5.29), Tyr341
											**π-Donor Hydrogen**	(5.88)
											**Bond:** Tyr124 (2.19)	
Scopoletin	−8.202	−26.348	−36.51	−12.63	−19.46	2.29	−1.13	−18.24	24.79	−20.74	**Conventional Hydrogen**	**π-π Stacked:** Trp286
											**Bond:** Phe295 (1.75),	(4.92), Tyr341 (4.82),
											Tyr337 (1.91),	Tyr341 (3.96),
											**Carbon Hydrogen**	**π-π T-shaped:** Tyr124
											**Bond:** Val294 (2.54),	(5.61),
											Tyr124 (2.46), Tyr124	**π-Alkyl:** Tyr72 (4.61),
											(2.65)	Tyr124 (4.98)
Biochanin A	−7.901	−34.324	−38.01	−13.28	−17.33	4.71	−1.28	−9.95	26.69	−31.73	**Conventional Hydrogen**	**π-π Stacked:** Trp286
											**Bond:** Phe295 (1.97),	(5.11), Trp286 (4.75),
											Tyr337 (2.79),	Trp286 (3.74), Trp286
											**Carbon Hydrogen**	(4.92), Trp286 (4.33),
											**Bond:** Trp286 (2.65)	**Alkyl:** Leu289 (5.05)
Vasicine	−7.848	−30.824	−41.04	−14.59	−21.56	0.44	−0.59	−8.98	20.65	−26	**Conventional Hydrogen**	**π-π Stacked:** Trp286
											**Bond:** Phe295 (1.80),	(4.39), Trp286 (5.65),
											**Carbon Hydrogen**	**π-π T-shaped:** Tyr124
											**Bond:** Val294 (2.61),	(5.64),
											Tyr341 (2.78), Ser293	**π-Alkyl:** Trp286 (4.19)
											(2.52), Ser293 (2.66),	
											Tyr341 (2.90)	
Galanthamine	−9.742	−43.067	−23.61	−7.02	−62.45	3.84	−1.62	−30.51	88.88	-20.93	**Conventional Hydrogen**	**Electrostatic Attractive**
											**Bond:** Ser203 (2.00),	**Charge:** Asp74 (4.50),
											Glu202 (1.74), Tyr337	**π-π T-shaped:** Tyr337
											(2.16),	(5.83),
											**Carbon Hydrogen**	**Amide-π Stacked:**
											**Bond:** His447 (2.41),	Gly121 (4.04), Gly122
											Tyr133 (2.86), Ser125	(4.04),
											(2.91), Asp74 (2.59)	**π-Alkyl:** Trp86 (4.19),
												Trp86 (3.91), Phe295
												(4.18), Phe297 (4.50),
												Phe338 (4.66), His447
												(4.64)

ΔGBinding: Binding free energy; Log Ki: Logarithmic Inhibition Constant (Ki); ΔGCoulomb: Coulomb binding energy; ΔGCovalent: Covalent binding energy; ΔGHbond Hydrogen bonding energy; ΔGLipo: Lipophilic binding energy; ΔGSolv GB: Generalized born electrostatic solvation energy; ΔGvdW: Energy of van der Waals forces; All of these energies contribute to ΔGBinding (Binding free energy).

## Data Availability

The raw data of the current study are available from the corresponding author upon request due to the privacy.

## Supplementary Materials

The following supporting information can be downloaded at: https://www.mdpi.com/article/10.3390/metabo13070794/s1, Supplementary Figure S1. Experimental timeline for behavioral studies including animal grouping and group-wise treatments; Supplementary Figure S2. Percentage (%) inhibition of 2,2-Diphenyl-1-picrylhydrazyl (DPPH), total antioxidant capacity (TAC), total reducing power (TRP) ability and acetylcholinesterase (AChE) inhibitory assays; Supplementary Figure S3. Photomicrographs of CLAE effect on the histology of vital organs after 14 and 28 days of toxicity study; Supplementary Figure S4. The track plots of random animals for the exploration of novel object from each group in NOR test; Supplementary Figure S5. The chemical structures and ESI-MS/MS spectra of 53 tentatively identified phytochemicals in negative and positive modes of analysis; Supplementary Figure S6. Venn diagram between CLAE phytochemicals and disease-target genes related to myocardial infarction, anxiety, depression and memory deficits; Supplementary Figure S7. GO biological processes and KEGG analysis of disease-target genes associated with myocardial infarction, anxiety, depression and memory deficits; Table S1. Effects of *Conocarpus lancifolius* aqueous extract (CLAE) in behavioral novel object recognition (NOR) test on the recognition memory of experimental rats; Table S2. Prediction of drug-likeness parameters of selected CLAE phytoconstituents by using SwissADME; Table S3. Prediction of absorption, distribution, metabolism, excretion and toxicity (ADMET) properties of selected CLAE compounds by using pkCSM program; Table S4. Myocardial infarction related pathogenic genes for CLAE; Table S5. Anxiety, depression and impaired memory related pathogenic genes for CLAE; Table S6. Myocardial infarction related pathogenic genes retrieved for the CLAE compounds; Table S7. Anxiety, depression and impaired memory related pathogenic genes retrieved for CLAE compounds; Table S8. Top 20 GO biological process of CLAE phytocompounds for myocardial infarction related target genes; Table S9. Top 20 KEGG pathway of CLAE phytocompounds for myocardial infarction related target genes; Table S10. Top 20 GO biological process of CLAE phytocompounds for anxiety, depression and impaired memory related target genes; Table S11. Top 20 KEGG pathway of CLAE phytocompounds for anxiety, depression and impaired memory related target genes; Table S12. Network analysis of myocardial infarction related target genes inter-action with CLAE compounds; Table S13. Network analysis of myocardial infarction related target genes interaction with CLAE compounds and GO biological process (BP); Table S14. Network analysis of myocardial infarction related target genes interaction with CLAE compounds and KEGG pathways; Table S15. Network analysis of anxiety, depression and impaired memory related target genes interaction with CLAE constituents; Table S16. Network analysis of anxiety, depression and impaired memory related target genes interaction with CLAE compounds and GO biological process (BP); Table S17. Network analysis of anxiety, depression and impaired memory related target genes interaction with CLAE constituents and KEGG signaling pathways.
