# Supplementary material for: Conocarpus lancifolius (Combretaceae): Pharmacological Effects, LC-ESI-MS/MS Profiling and In Silico Attributes
Source: Metabolites. 2023 Jun 27;13(7):794. doi: 10.3390/metabo13070794 (PMC10385132; doi:10.3390/metabo13070794)
Supplement: Supplementary file 1 [file metabolites-13-00794-s001.zip › metabolites-2427132-supplementary.pdf]

Supplementary Materials

Supplementary Figure S1. Experimental timeline for behavioral studies including animal grouping and group-wise treatments; Supplementary Figure S2. Percentage (%) inhibition of 2,2-Diphenyl-1-picrylhydrazyl (DPPH), total antioxidant capacity (TAC), total reducing power (TRP) ability and acetylcholinesterase (AChE) inhibitory assays; Supplementary Figure S3. Photomicrographs of CLAE effect on the histology of vital organs after 14 and 28 days of toxicity study; Supplementary Figure S4. The track plots of random animals for the exploration of novel object from each group in NOR test; Supplementary Figure S5. The chemical structures and ESI-MS/MS spectra of 53 tentatively identified phytochemicals in negative and positive modes of analysis; Supplementary Figure S6. Venn diagram between CLAE phytochemicals and disease-target genes related to myocardial infarction, anxiety, depression and memory deficits; Supplementary Figure S7. GO biological pro-cesses and KEGG analysis of disease-target genes associated with myocardial infarction, anxiety, depression and memory deficits; Table S1. Effects of *Conocarpus lancifolius* aqueous extract (CLAE) in behavioral novel object recognition (NOR) test on the recognition memory of experimental rats; Table S2. Prediction of drug-likeness parameters of selected CLAE phytoconstituents by using SwissADME; Table S3. Prediction of absorption, distribution, metabolism, excretion and toxicity (ADMET) properties of selected CLAE compounds by using pkCSM program; Table S4. Myocardial infarction related pathogenic genes for CLAE; Table S5. Anxiety, depression and impaired memory related pathogenic genes for CLAE; Table S6. Myocardial infarction related pathogenic genes retrieved for the CLAE compounds; Table S7. Anxiety, depression and impaired memory related pathogenic genes retrieved for CLAE compounds; Table S8. Top 20 GO biological process of CLAE phytocompounds for myocardial infarction related target genes; Table S9. Top 20 KEGG pathway of CLAE phytocompounds for myocardial infarction related target genes; Table S10. Top 20 GO biological process of CLAE phytocompounds for anxiety, depression and impaired memory related target genes; Table S11. Top 20 KEGG pathway of CLAE phytocompounds for anxiety, depression and impaired memory related target genes; Table S12. Network analysis of myocardial infarction related target genes inter-action with CLAE compounds; Table S13. Network analysis of myocardial infarction related target genes interaction with CLAE compounds and GO biological process (BP); Table S14. Network analysis of myocardial infarction related target genes interaction with CLAE compounds and KEGG pathways; Table S15. Network analysis of anxiety, depression and impaired memory related target genes interaction with CLAE constituents; Table S16. Network analysis of anxiety, depression and impaired memory related target genes interaction with CLAE compounds and GO biological process (BP); Table S17. Network analysis of anxiety, depression and impaired memory related target genes interaction with CLAE constituents and KEGG signaling pathways.

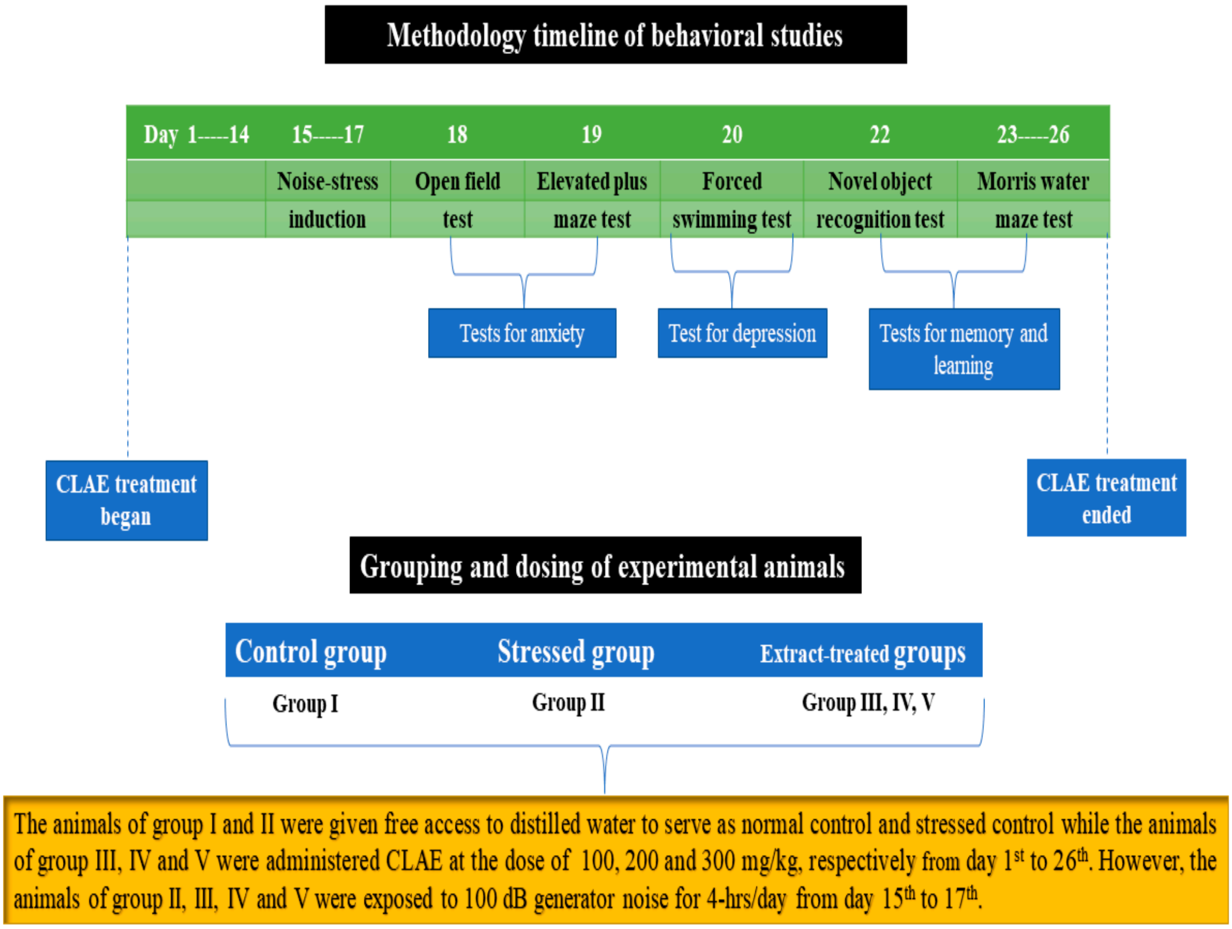

Supplementary Figure S1. Experimental timeline for behavioral studies including animal grouping and group-wise treatments.

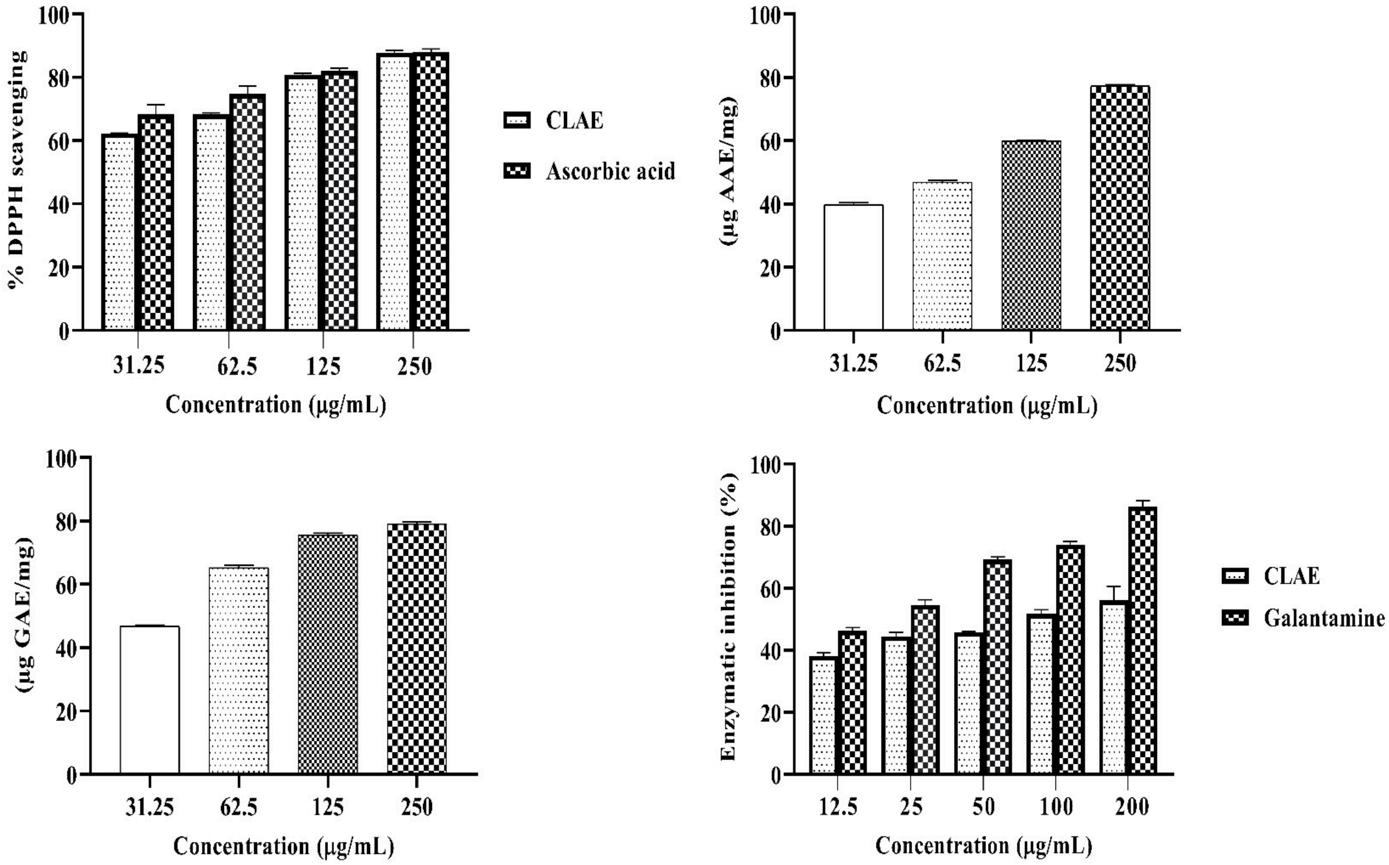

Supplementary Figure S2. Percentage (%) inhibition of DPPH radial scavenging assay **(A)**; Total antioxidant capacity (TAC) of CLAE in terms of microgram ascorbic acid equivalents per milligram (µg AAE/mg) of the dry extract **(B)**; Total reducing power (TRP) ability of CLAE in terms of microgram gallic acid equivalents (µg GAE/mg of dry weight extract) **(C)**; % inhibition of acetylcholinesterase (AChE) inhibitory assay **(D)**. Data is expressed as mean ± standard deviation (SD) of three replicates.

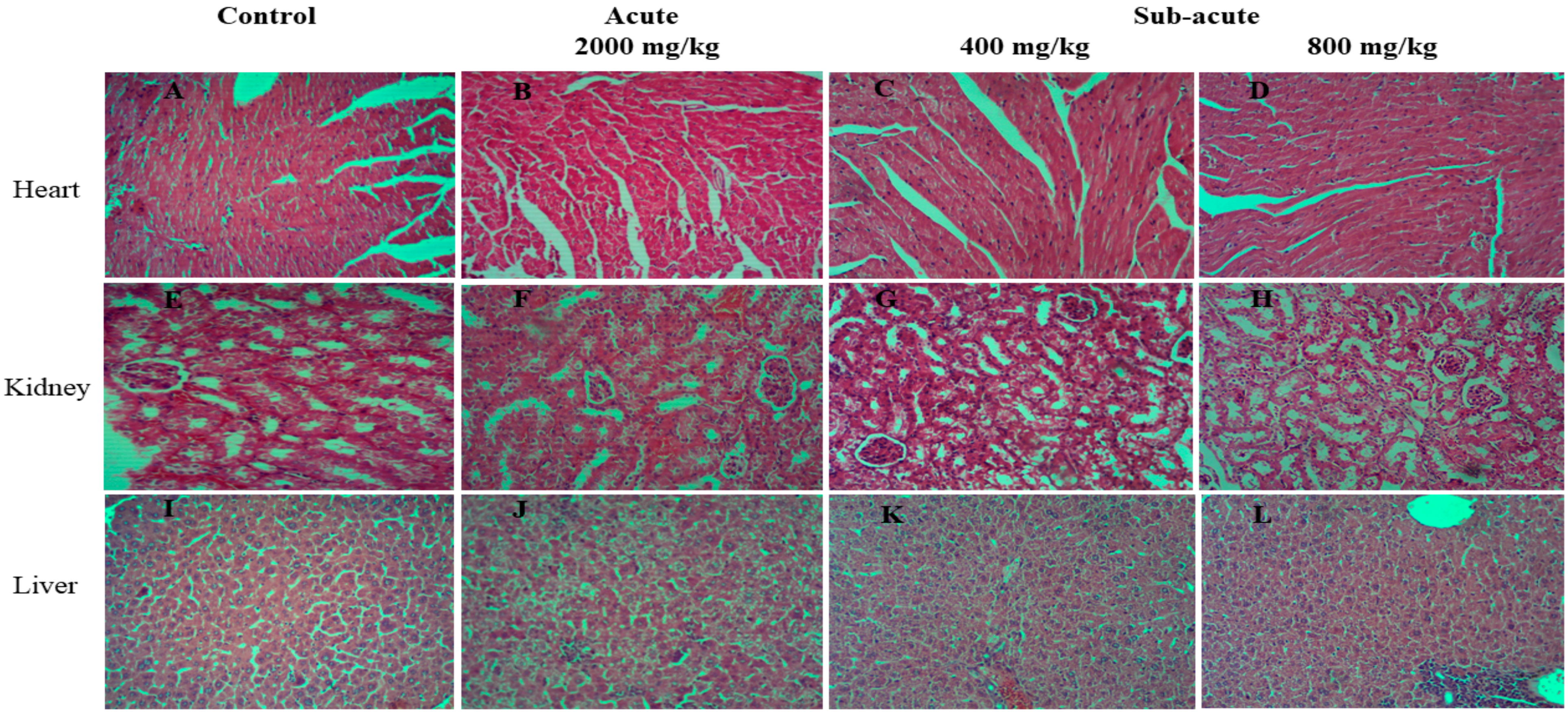

Supplementary Figure S3. Photomicrographs (H&E staining, 10 x magnification) of CLAE effect on the histology of vital organs after 14 and 28 days of toxicity study. (**A**) control group; (**B**) 2000 mg/kg CLAE; (**C**) 400 mg/kg CLAE; and (**D**) 800 mg/kg CLAE group showed normal heart architecture. In kidney (**E**) control group and (**F**) 2000 mg/kg CLAE displayed normal histology whereas existence of cells in the renal tubular lumen, degenerated epithelial cells and mild inflammatory cells infiltration were observed in (**G**) 400 mg/kg CLAE and (**H**) 800 mg/kg CLAE treated groups. In liver tissues (**I**) control group displayed normal structure; (**J**) 2000 mg/kg CLAE presented mild infiltration of inflammatory cells; (**K**) 400 mg/kg CLAE showed moderate infiltration of inflammatory cells and (**L**) 800 mg/kg CLAE treated group displayed severe inflammatory infiltrates.

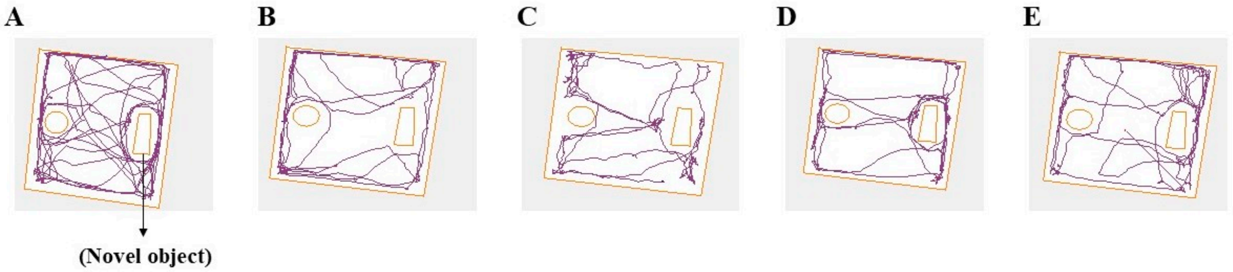

Supplementary Figure S4. The track plots of random animals for the exploration of novel object from each group in NOR test. (**A**) Control group; (**B**) Stressed group; (**C**) 100 mg/kg CLAE; (**D**) 200 mg/kg CLAE; and (**E**) 300 mg/kg CLAE. NOR test: Novel object recognition test.

L-Leucine

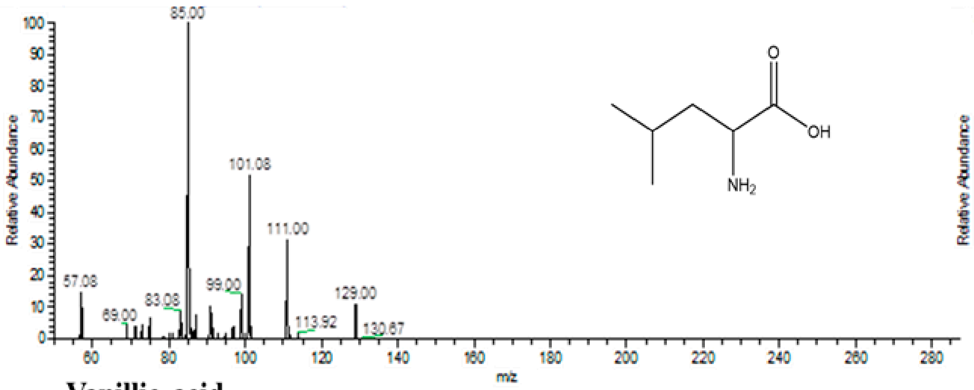

Malic acid

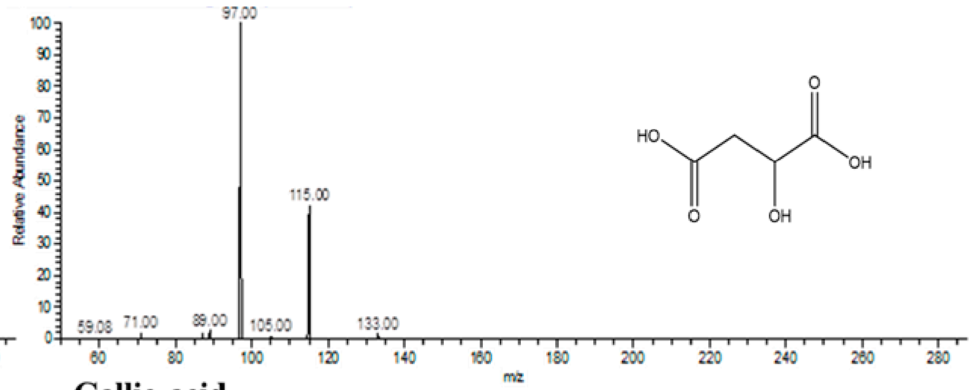

Vanillic acid

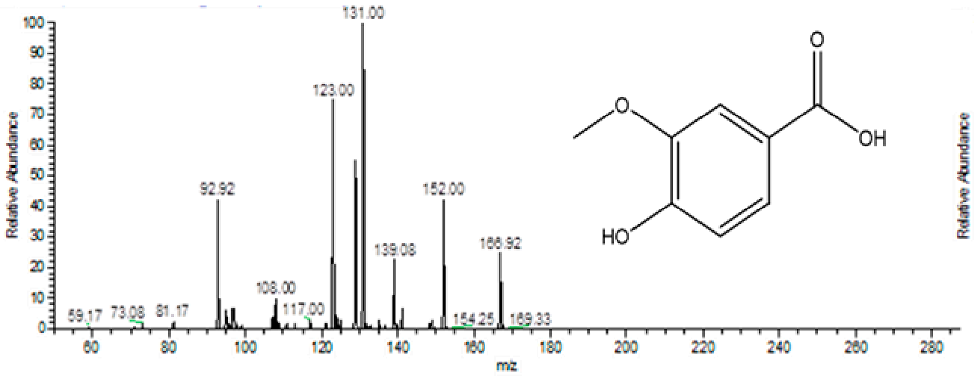

Gallic acid

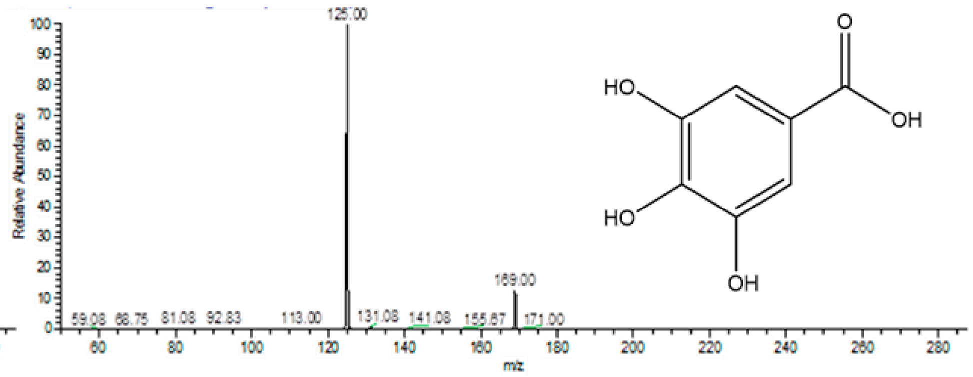

Syringic acid

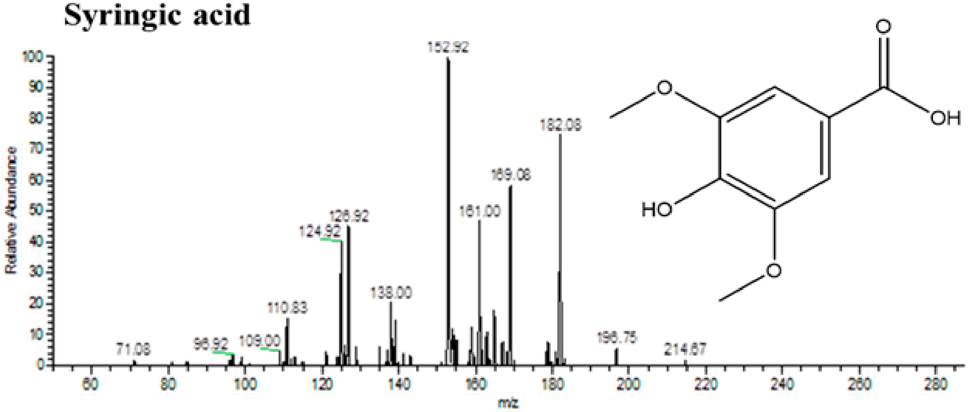

3,4-Dimethoxy-cinnamic acid

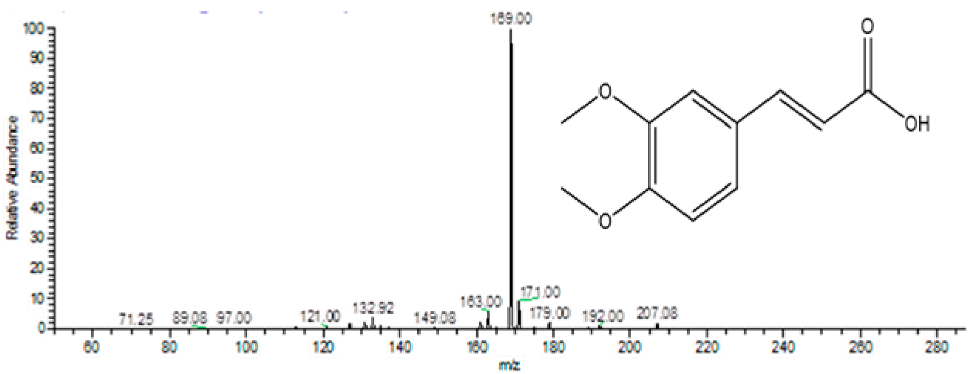

Capensine

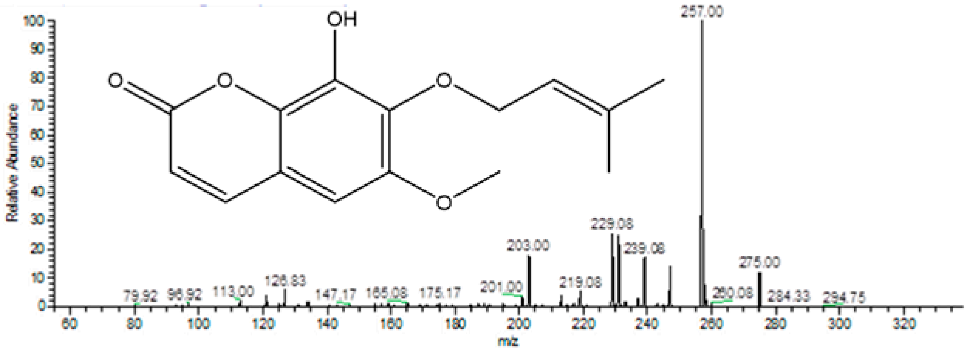

Fustin

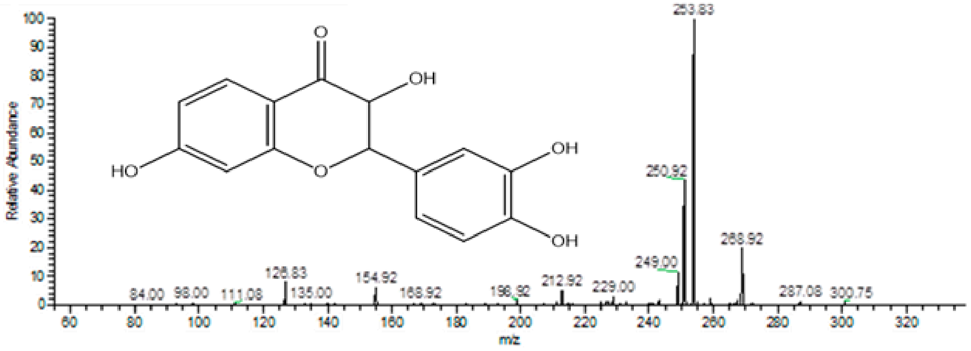

Enterolactone

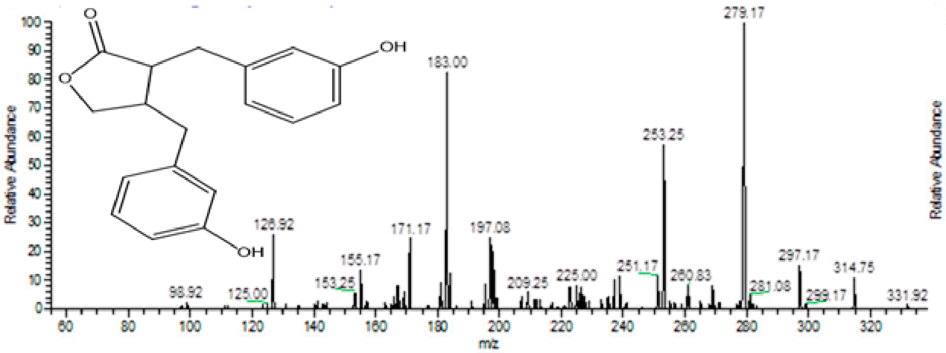

Quercetin

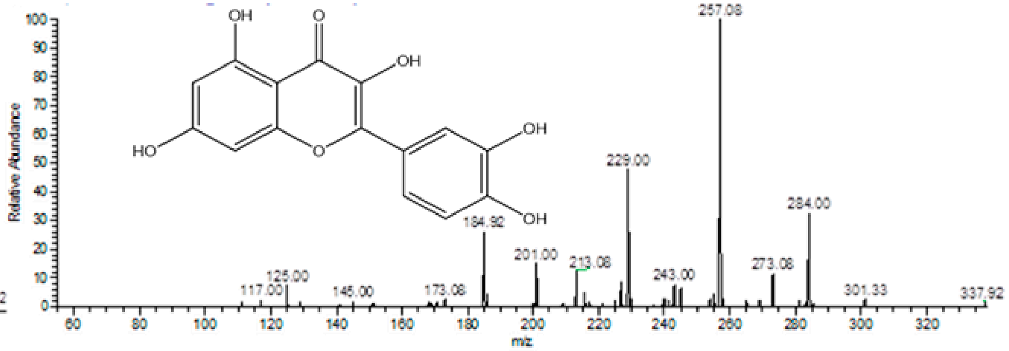

Epigallocatechin

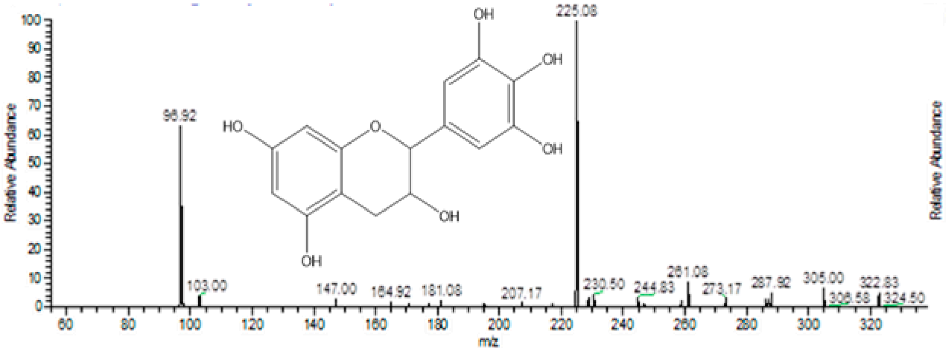

Andrographolide

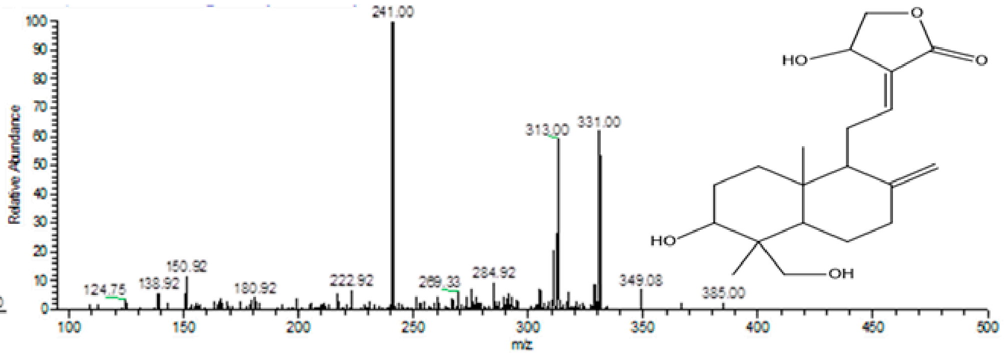

14,15-Dehydro-16-epi-vincamine

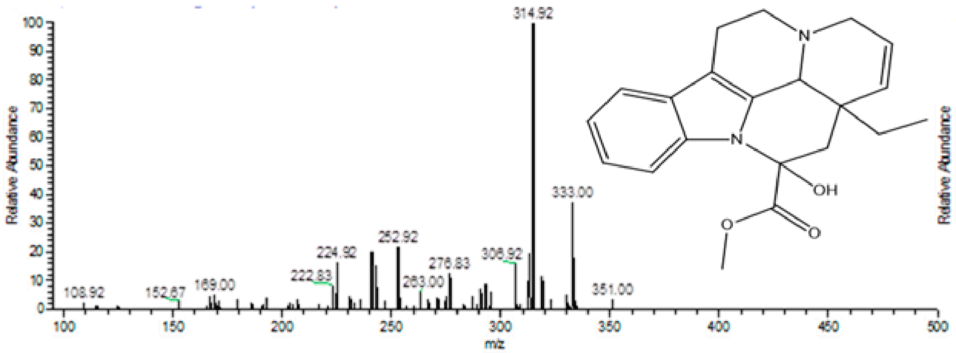

Otosenine

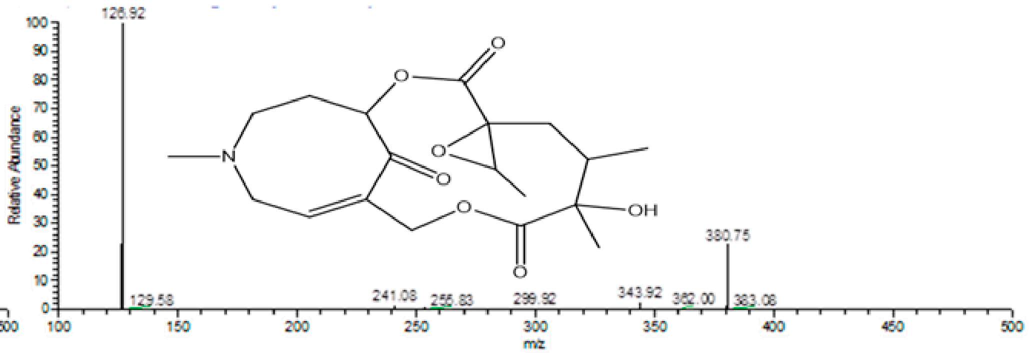

Voacristine

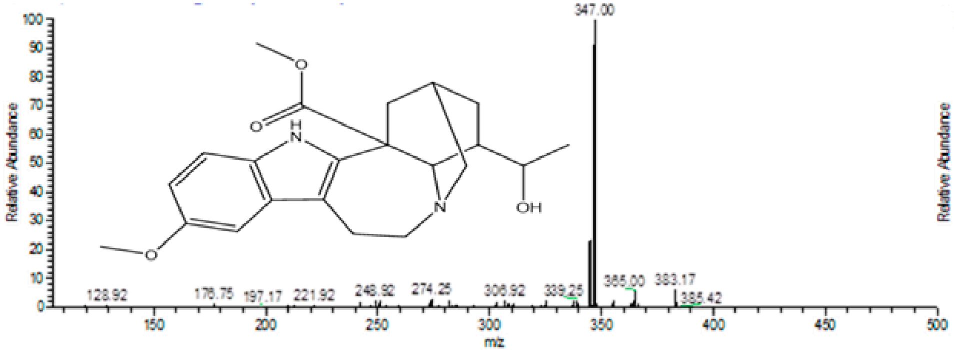

1-*O*-Sinapoylglucose

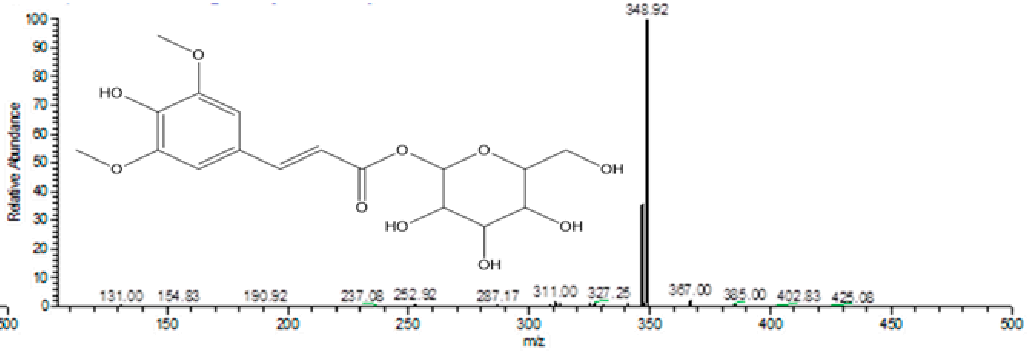

Scaposin

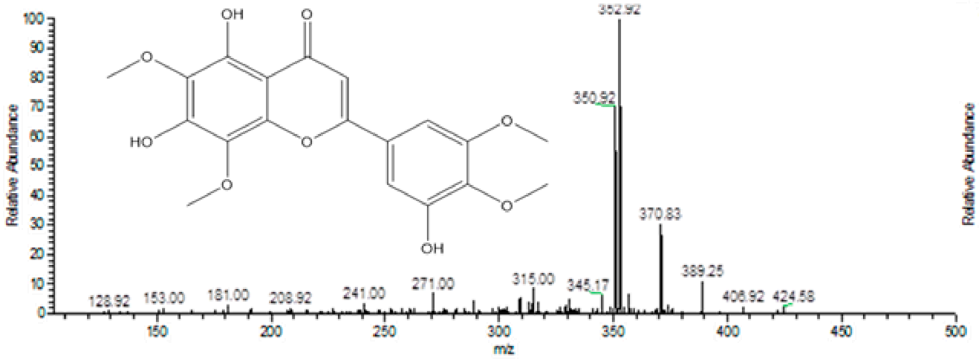

Kaempferol-3-*O*-arabinoside

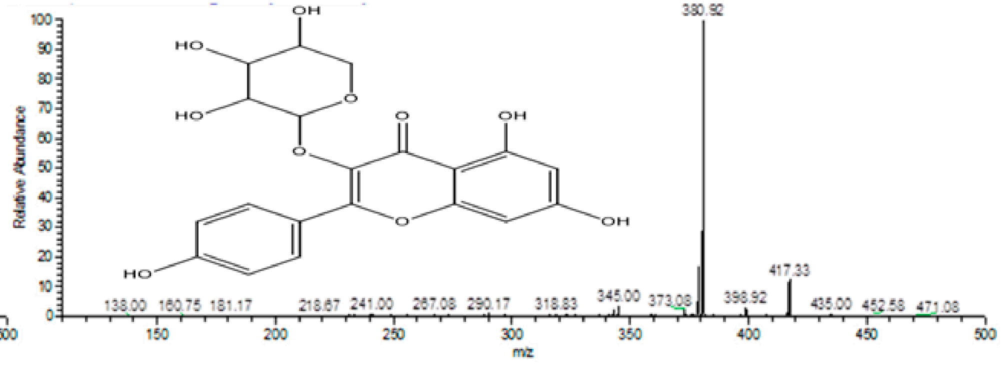

Isoangustone A

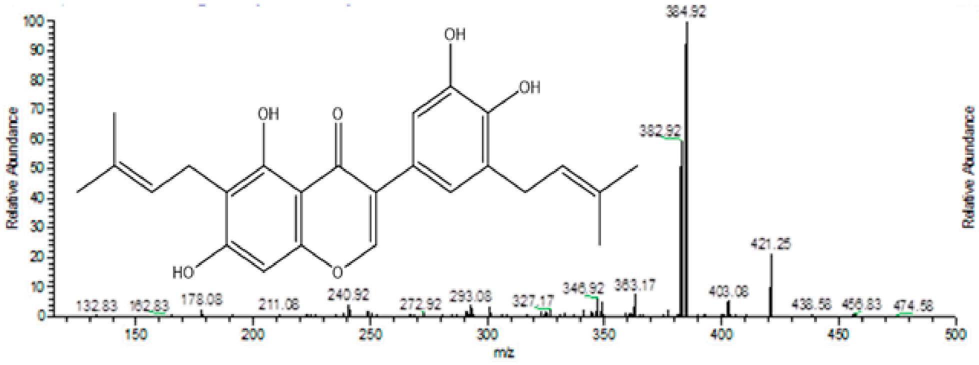

Gancaonin E

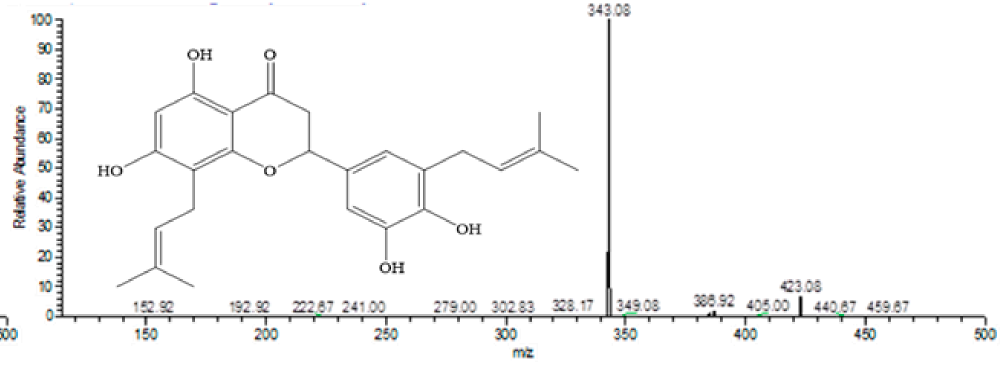

Naringenin-7-*O*-glucoside

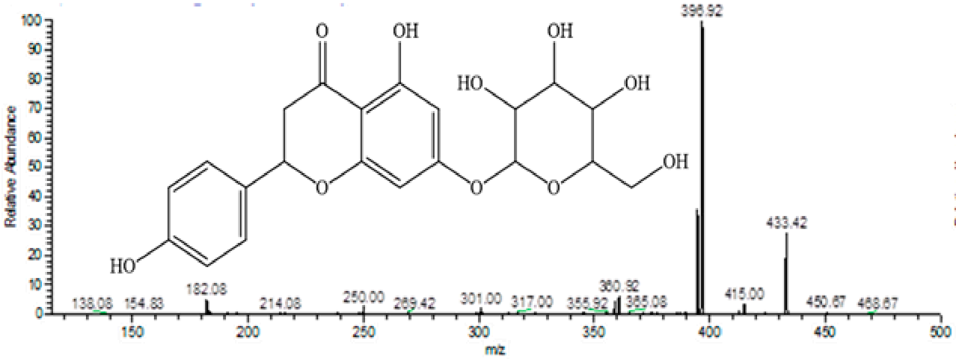

Isoorientin

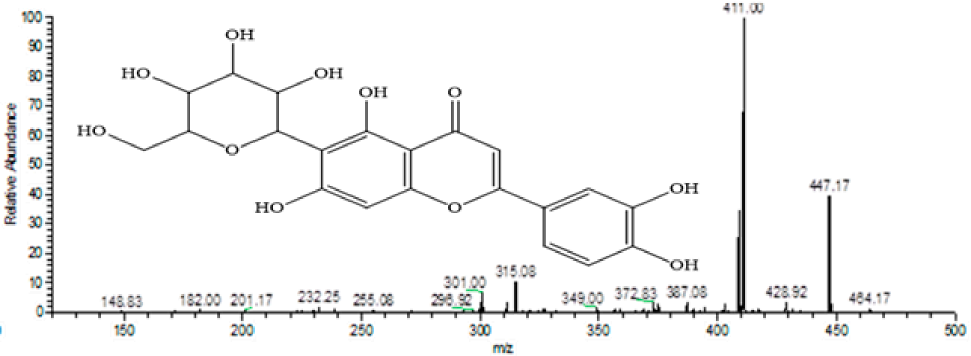

Isosilybin A

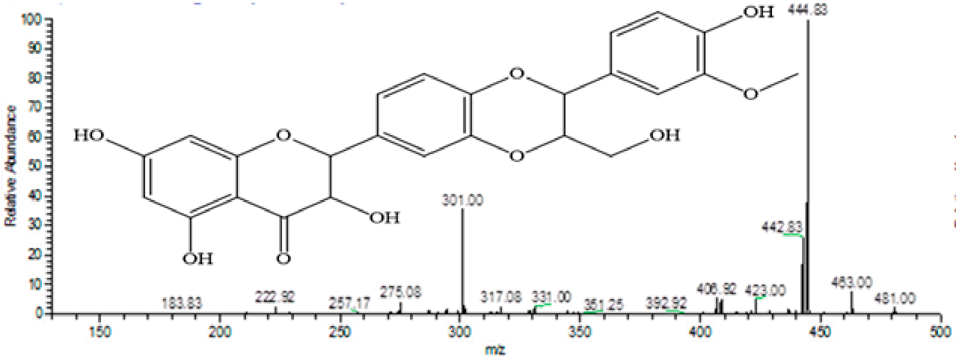

Harringtonine

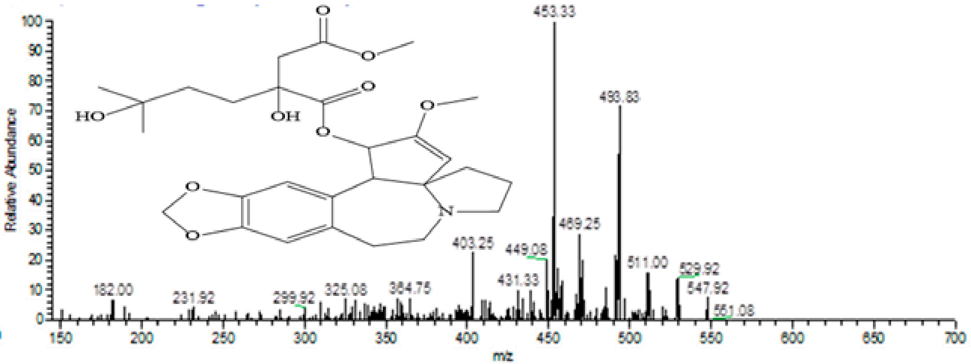

L-Proline

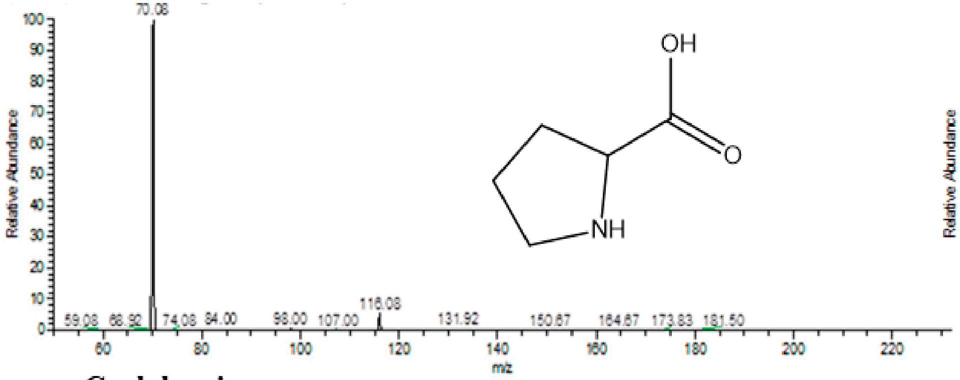

Benzoic acid

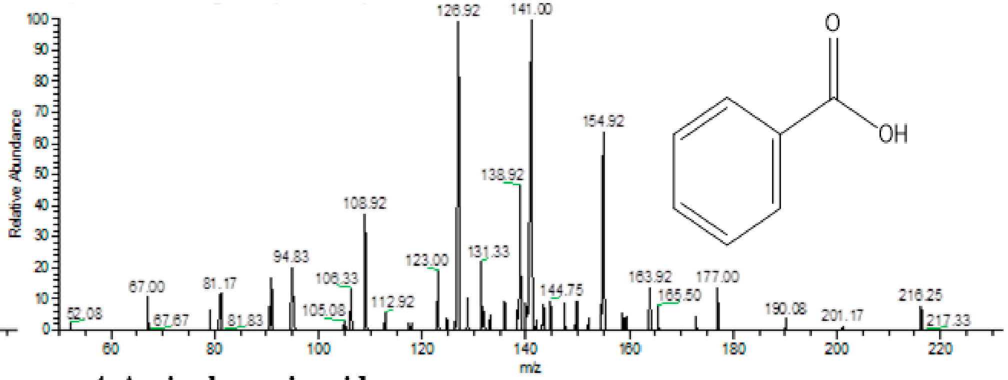

Cycloleucine

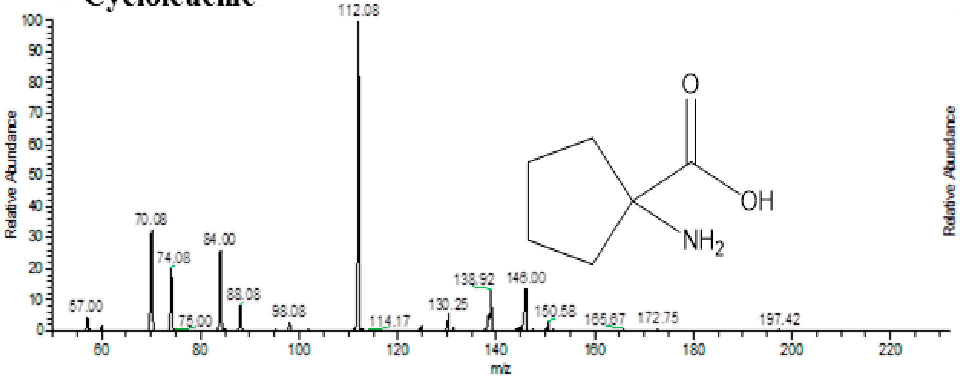

4-Aminobenzoicacid

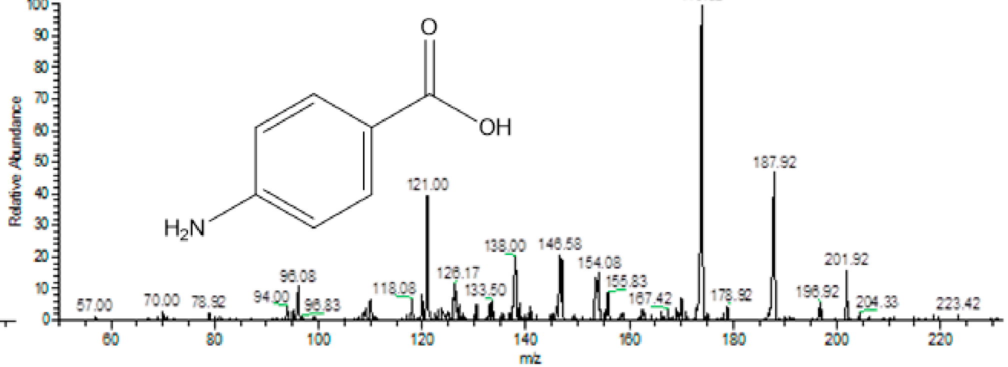

Glutamic acid

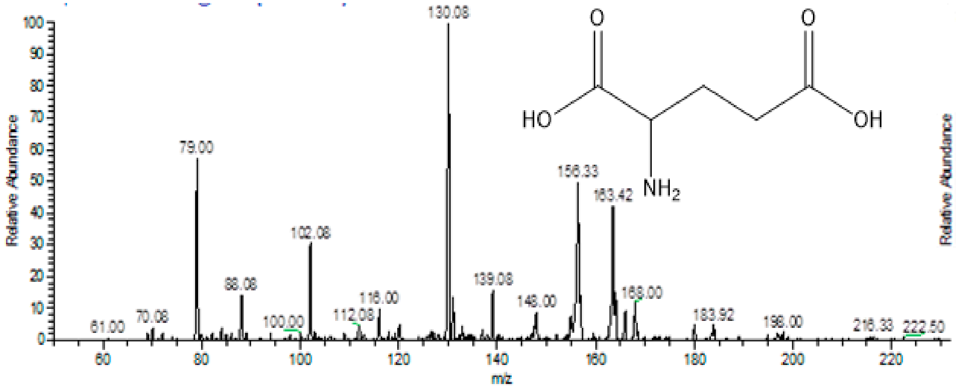

***p*-Coumaraldehyde**

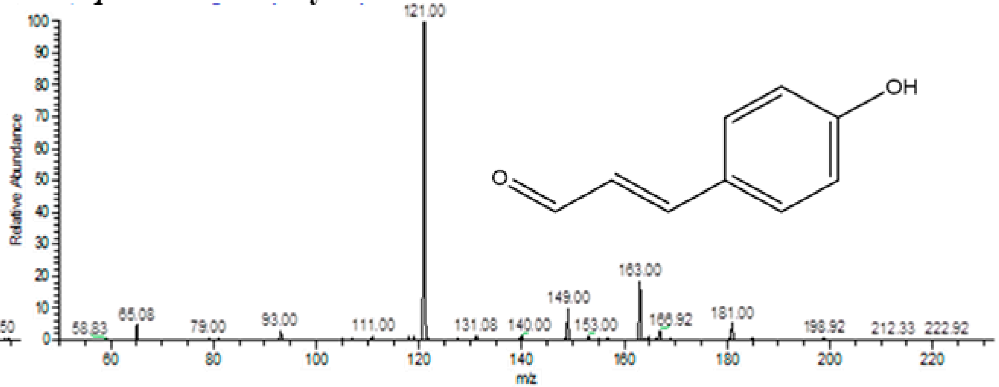

3-Indoleacetonitrile

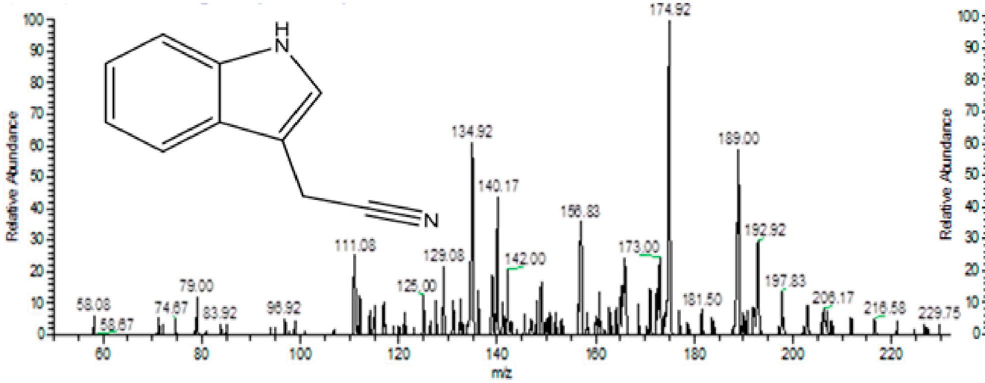

4-Hydroxycoumarin

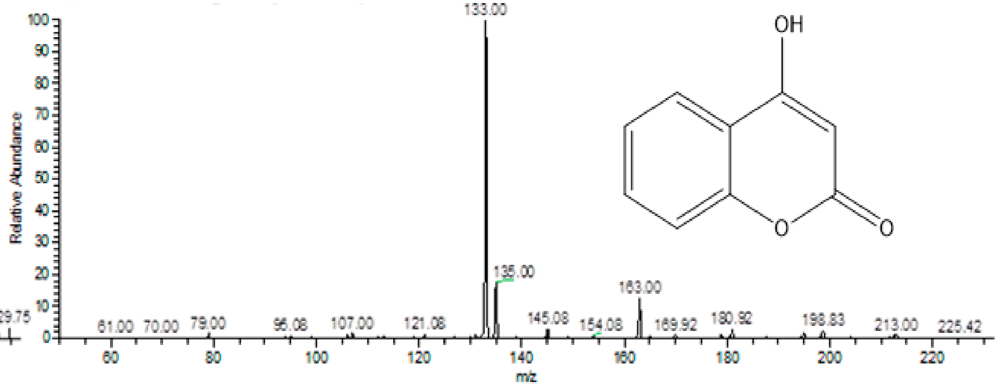

***cis*-Aconitic acid**

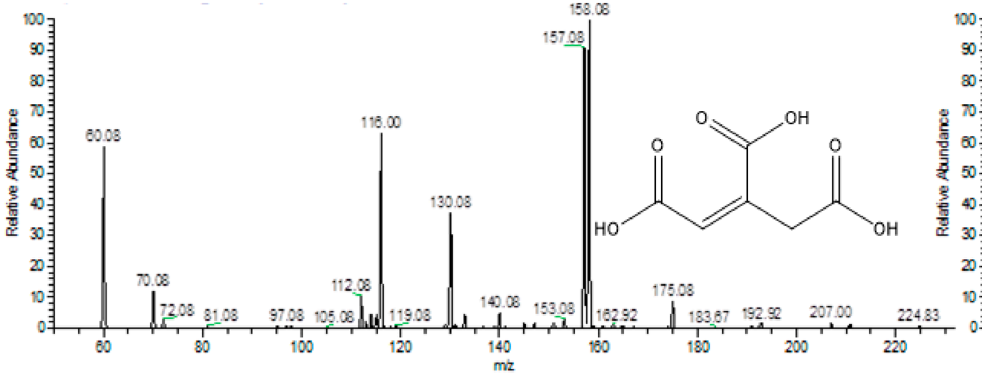

Caffeic acid

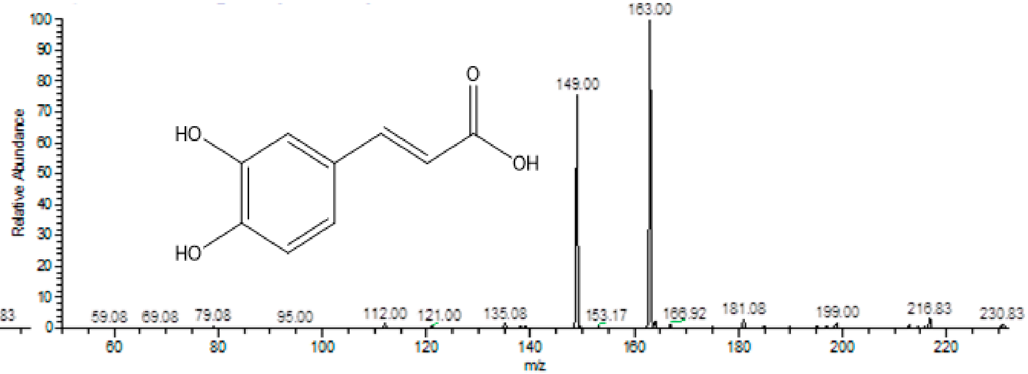

Vasicine

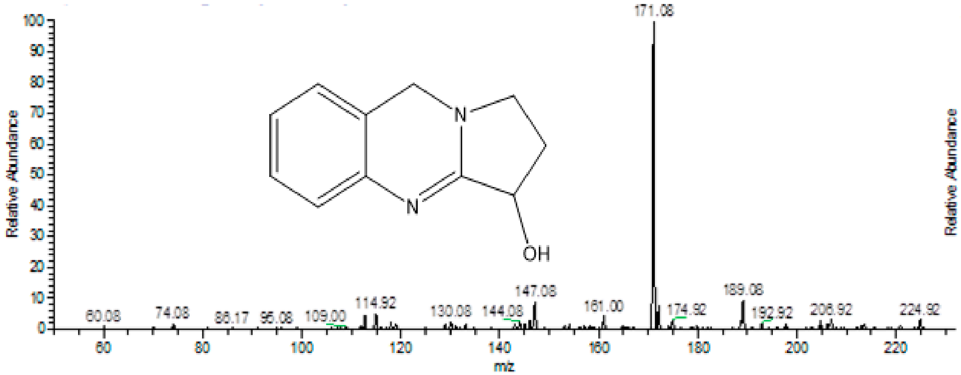

Scopoletin

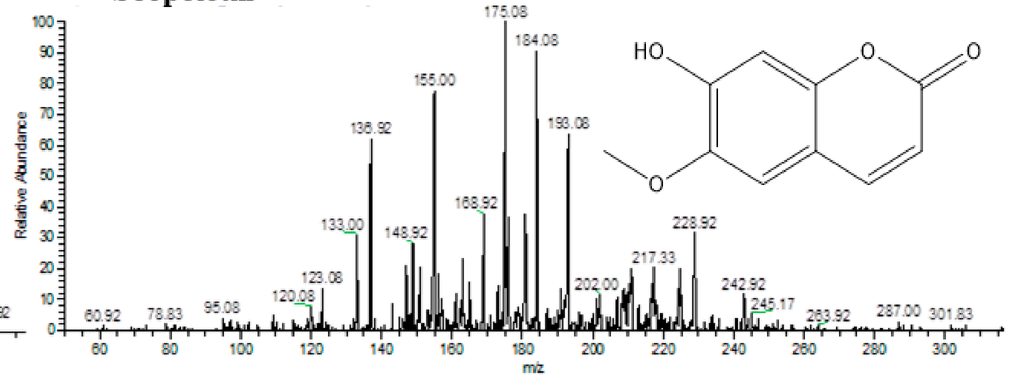

Vasicinone

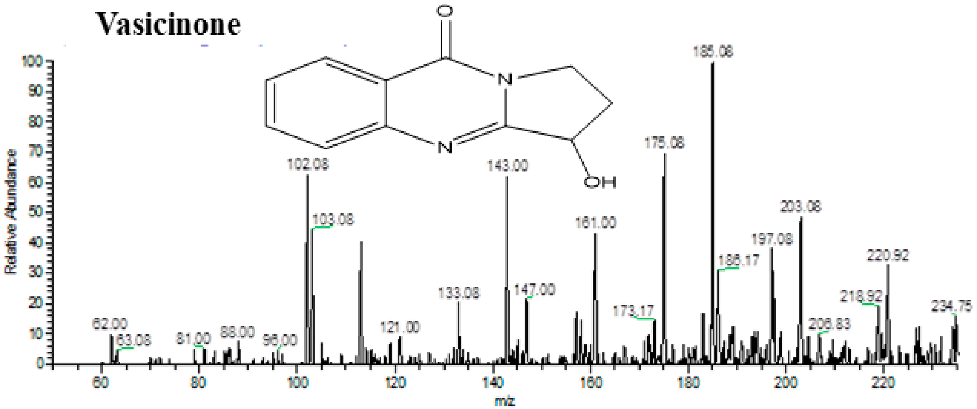

Tryptophan

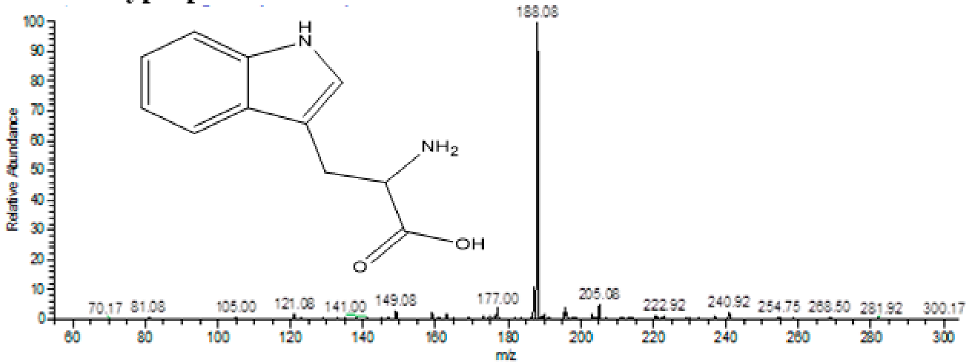

Harmaline

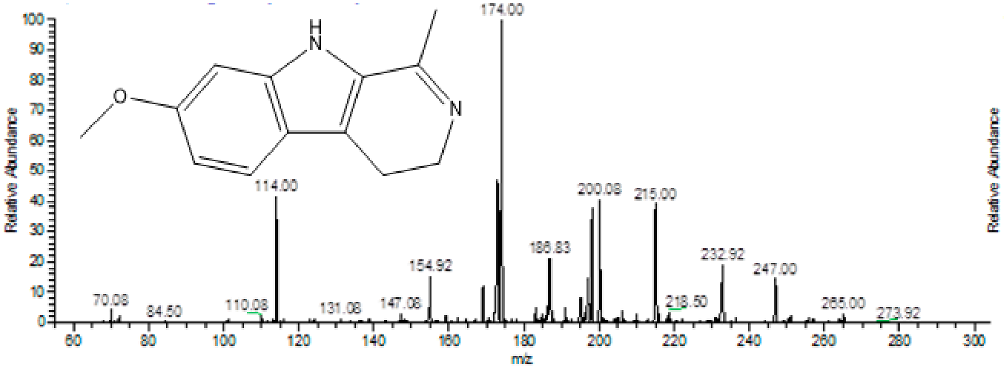

5,7-Dimethoxy-4-methylcoumarin

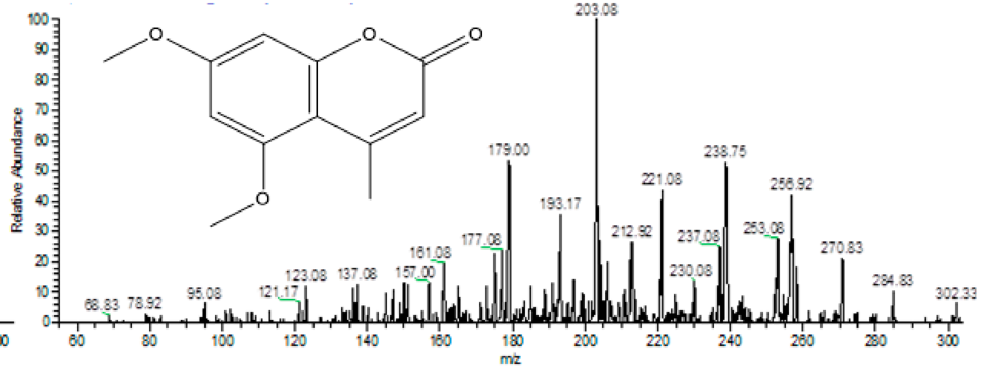

3,4,5-Trihydroxystilbene

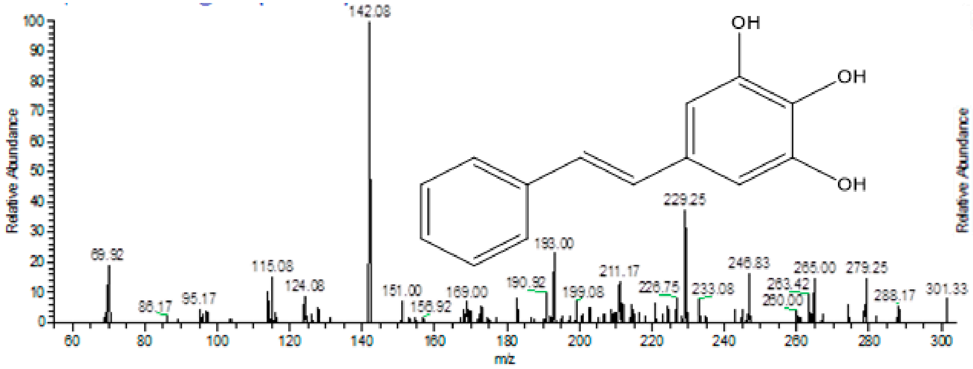

Chrysin

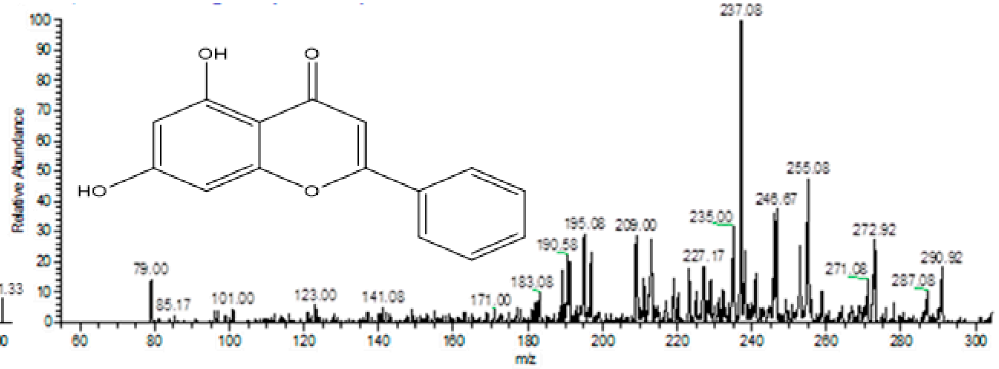

***(R)*-Semivioxanthin**

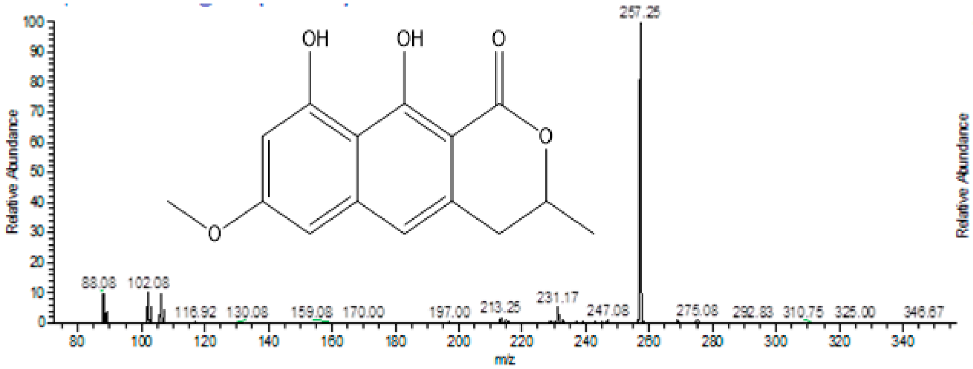

Biochanin A

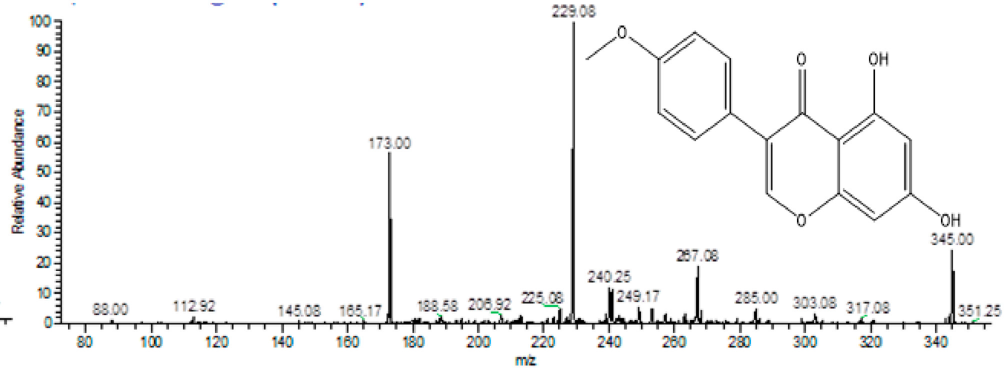

Ellagic acid

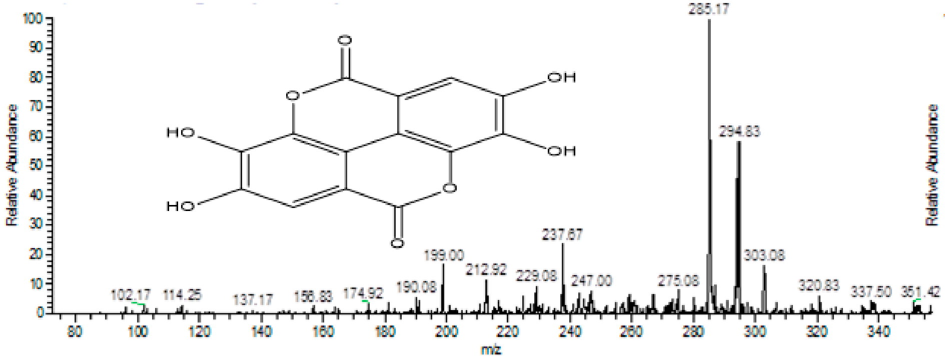

Taxifolin

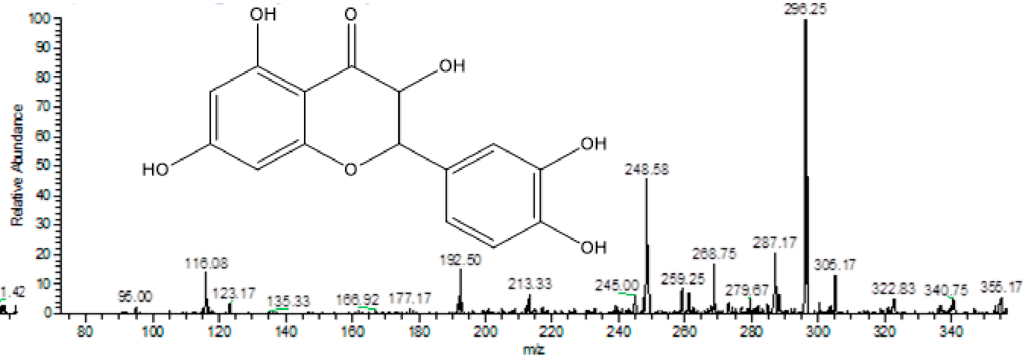

Justicidin B

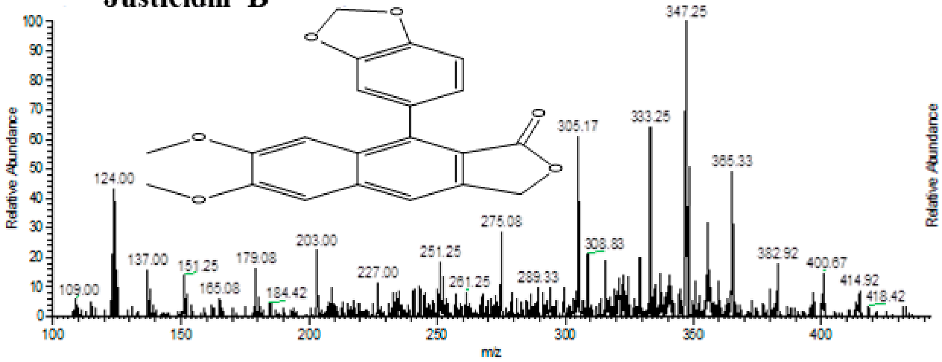

Maruchantin E

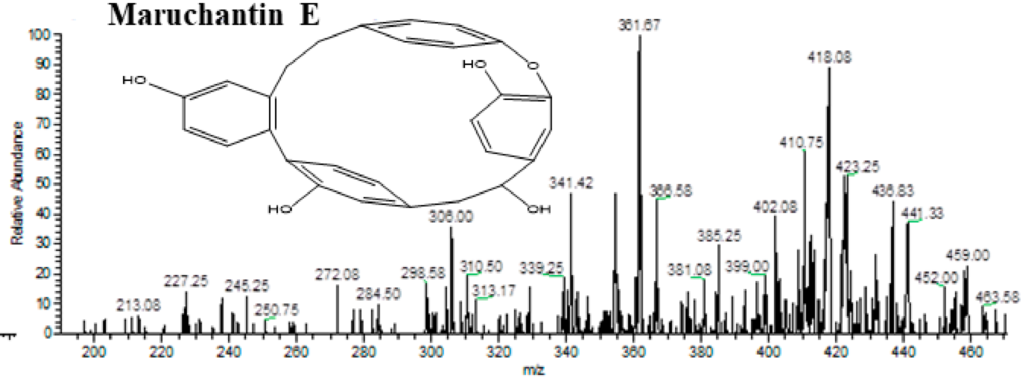

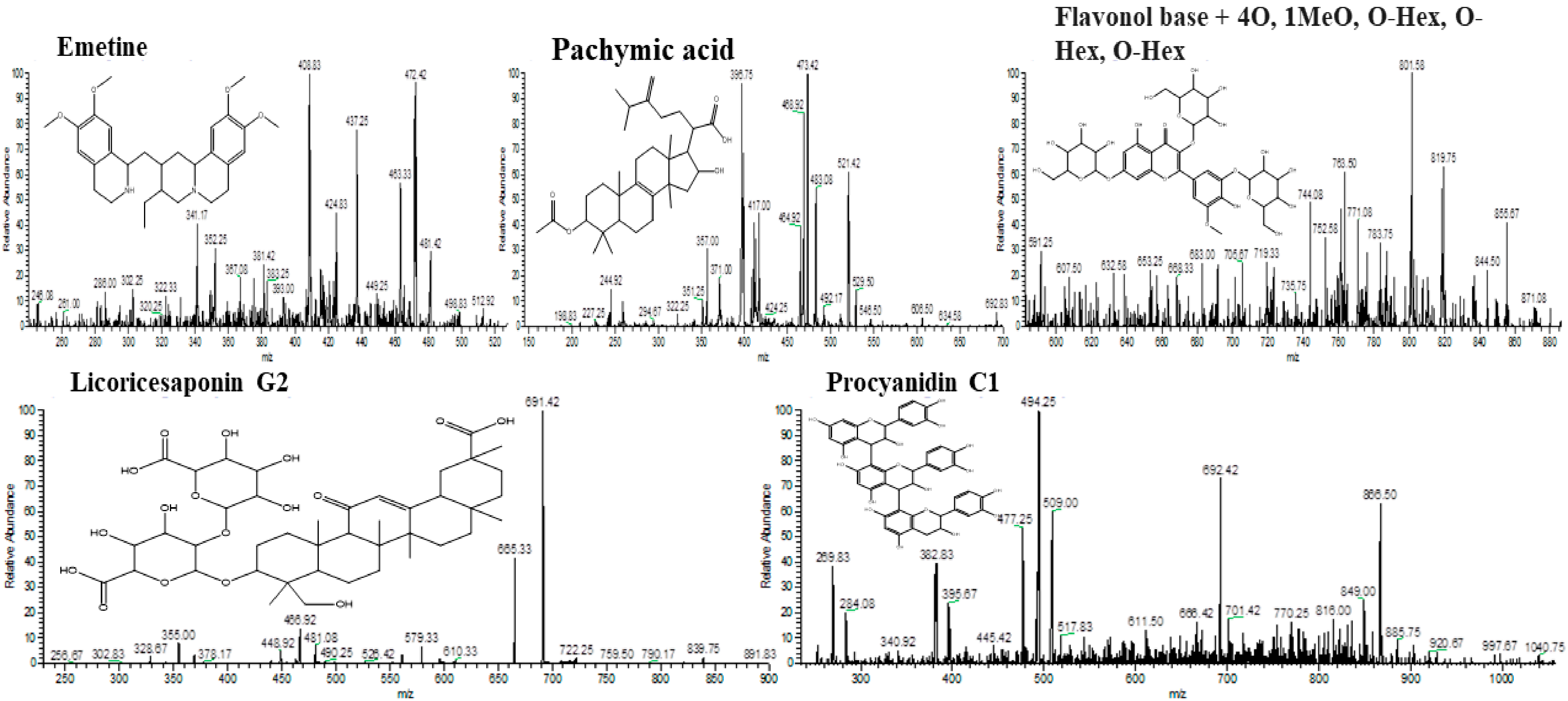

Supplementary Figure S5.
The chemical structures and ESI-MS/MS spectra of 53 tentatively identified phytochemicals in negative and positive modes of analysis.

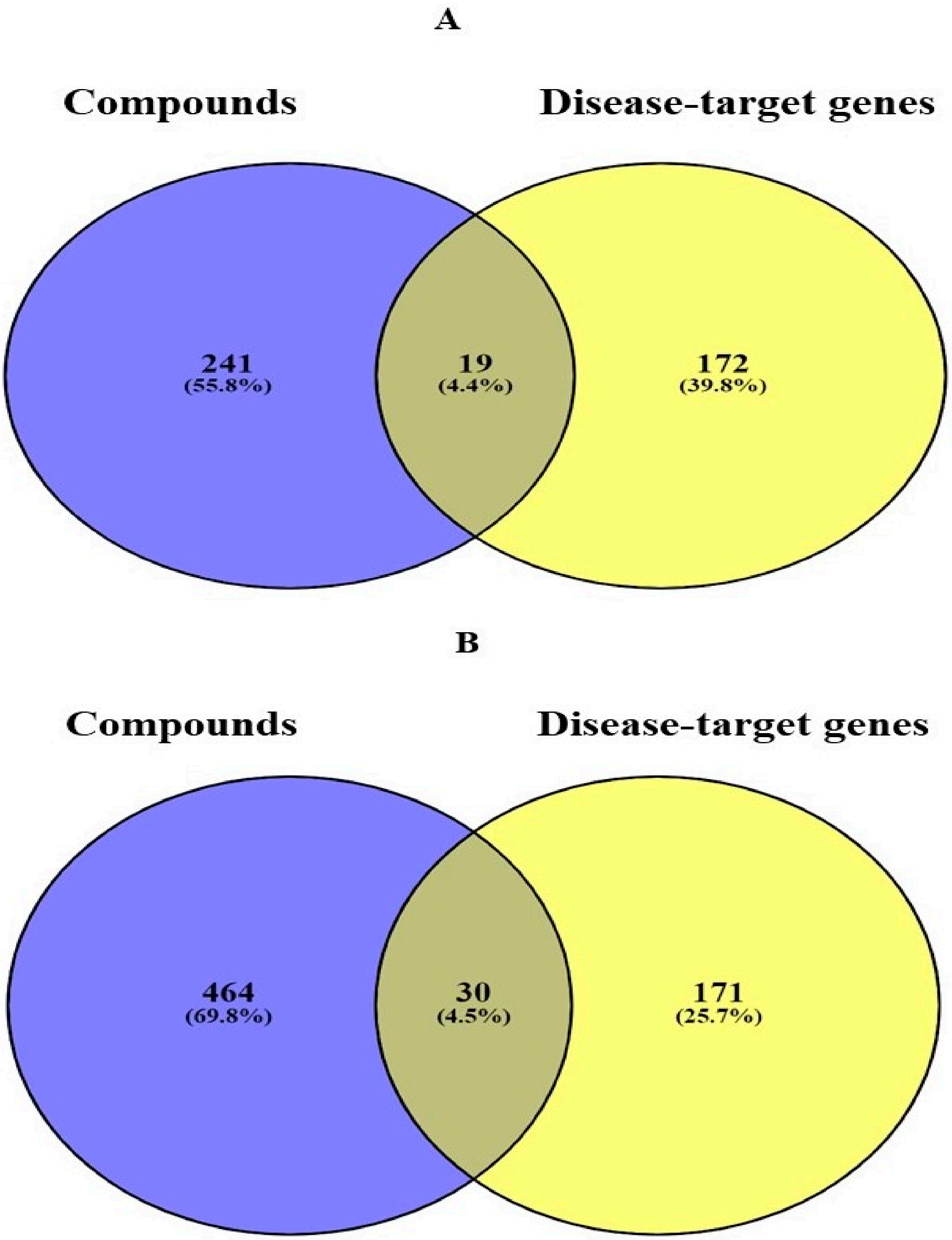

Supplementary Figure S6. Venn diagram between CLAE phytochemicals and disease-target genes related to myocardial infarction **(A.)** and anxiety, depression and memory deficits **(B.)**

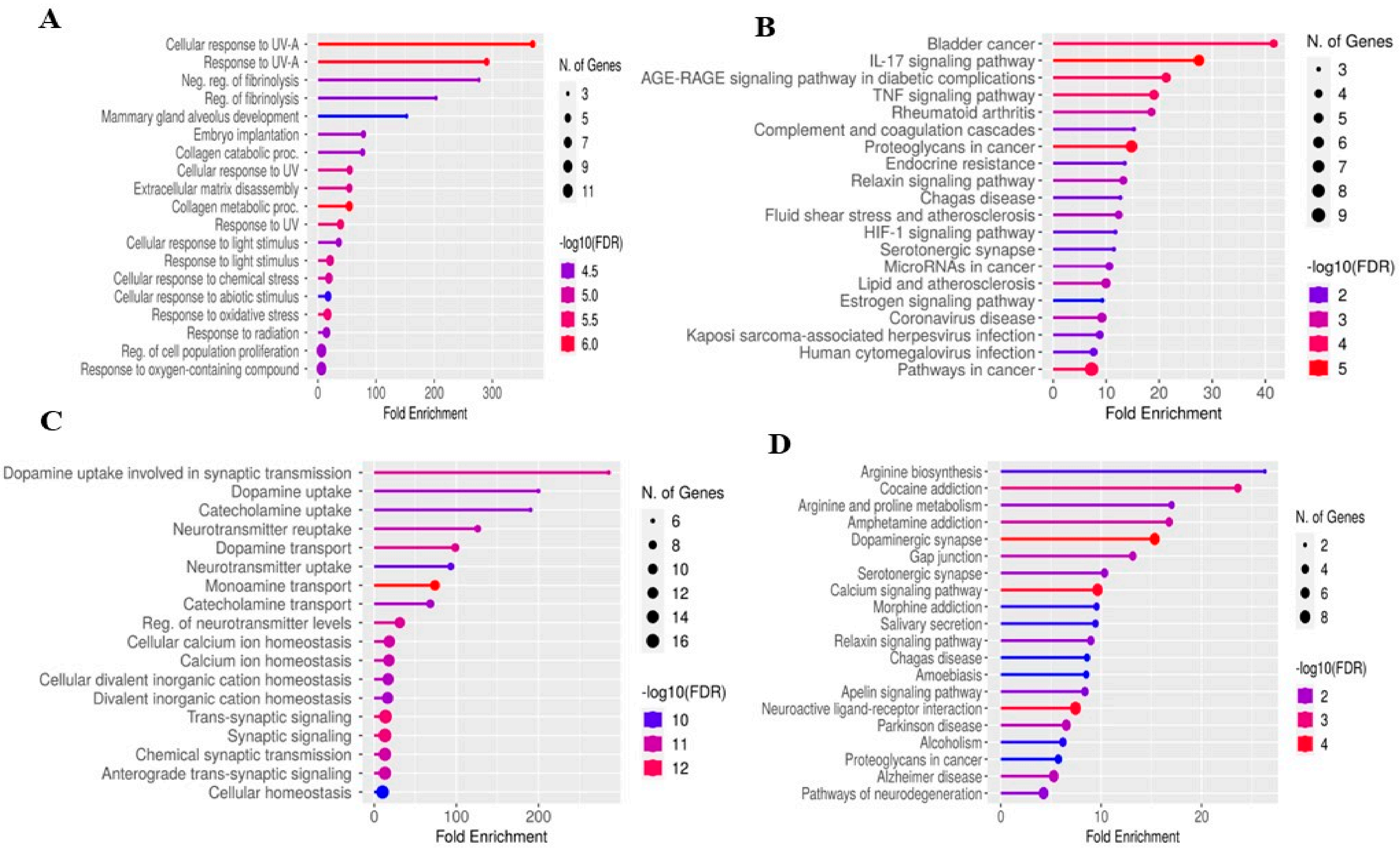

Supplementary Figure S7. GO biological processes **(A.)** and KEGG analysis **(B.)** of disease-target genes associated with myocardial infarction and GO biological processes **(C.)** and KEGG analysis **(D.)** of disease-target genes related to anxiety, depression and memory deficits.

Table S1. Effects of *Conocarpus lancifolius* aqueous extract (CLAE) in behavioral novel object recognition (NOR) test on the recognition memory of experimental rats.

Group/Dose	Test Phase	
	**Novel object exploration time (sec)**	**Time spent to explore familiar object (sec)**
Control	18.14 ± 3.37	5.26 ± 1.71
Stressed	8.98 ± 4.83 $^{\#\#}$	13.18 ± 8.51 $^{\#}$
100 mg/kg CLAE	9.62 ± 4.25	6.56 ± 2.27
200 mg/kg CLAE	11.62 ± 7.06	6.32 ± 2.12
300 mg/kg CLAE	12.52 ± 12.44	5.98 ± 1.97 *

Results are expressed as mean ± SD (n=5). Data was analyzed by using one way ANOVA followed by Dunnett's multiple comparison test. $^{\#}$ $p < 0.05$ and $^{\#\#}$ $p < 0.01$ comparisons between control and stressed groups; * $p < 0.05$ comparison between stressed and CLAE-treated groups (300 mg/kg).

Table S2. Prediction of drug-likeness parameters of selected CLAE phytoconstituents by using SwissADME.

Compound name	M.W	HBAs	HBDs	XLOGP3	Log *S* (ESOL model)	TPSA (Å^2)	Flexibility RBs	Inhibitors CYP2D6	Inhibitors CYP3A4	PAINS alerts	Ro5 violations
Malic acid	134.02	5	3	-1.26	0.32	94.83	3	No	No	0	0
Vanillic acid	168.04	4	2	1.43	-2.02	66.76	2	No	No	0	0
Gallic acid	170.02	5	4	0.7	-1.64	97.99	1	No	Yes	1	0
Syringic acid	198.05	5	2	1.04	-1.84	75.99	3	No	No	0	0
3,4-Dimethoxycinnamic acid	208.21	4	1	2.34	-2.64	55.76	4	No	No	0	0
Capensine	276.09	5	1	3	-3.55	68.9	4	No	No	0	0
Fustin	288.06	6	4	0.87	-2.53	107.22	1	No	No	1	0
Enterolactone	298.33	4	2	3.32	-3.92	66.76	4	Yes	No	0	0
Quercetin	302.04	7	5	1.54	-3.16	131.36	1	Yes	Yes	1	0
Epigallocatechin	306.07	7	6	0.92	-2.08	130.61	1	No	No	1	1
Andrographolide	350.20	5	3	2.16	-3.18	86.99	3	No	No	0	0
14,15-Dehydro-16-epi-vincamine	352.43	4	1	2.75	-3.82	54.7	3	Yes	No	0	0
Otosenine	381.17	8	1	0.63	-2.6	105.67	0	No	No	0	0
Voacristine	384.47	5	2	2.36	-3.68	74.79	4	Yes	No	0	0
1-*O*-Sinapoylglucose	386.35	10	5	-0.34	-1.72	155.14	7	No	No	0	0
Scaposin	390.09	9	3	2.55	-3.96	127.82	5	No	Yes	0	0
Kaempferol-3-*O*-arabinoside	418.35	10	6	0.78	-3.12	170.05	3	No	No	0	1
Isoangustone A	422.17	6	4	6.16	-6.39	111.13	5	No	No	1	1
Gancaonin E	424.18	6	4	5.89	-6.14	107.22	5	No	Yes	1	1
Naringenin-7-*O*-glucoside	434.39	10	6	0.65	-2.97	166.14	4	No	No	0	1
Isoorientin	448.10	11	8	-0.15	-2.7	201.28	3	No	No	1	2
Isosilybin A	482.12	10	5	1.9	-4.14	155.14	4	No	Yes	0	0
Harringtonine	531.24	10	2	0.46	-2.88	123.99	10	Yes	Yes	0	1
Benzoic acid	122.03	2	1	1.87	-2.2	37.3	1	No	No	0	0
4-Aminobenzoic acid	137.04	2	2	0.83	-1.59	63.32	1	No	No	0	0

Compound name	M.W	HBAs	HBDs	XLOGP3	Log *S* (ESOL model)	TPSA (Å^2)	Flexibility RBs	Inhibitors CYP2D6	CYP3A4	PAINS alerts	Ro5 violations
p-Coumaraldehyde	148.16	2	1	1.75	-2.13	37.3	2	No	No	0	0
3-Indoleacetonitrile	156.06	1	1	1.60	-2.31	39.58	1	No	No	0	0
4-Hydroxycoumarin	162.03	3	1	1.27	-2.26	50.44	0	No	No	0	0
cis-Aconitic acid	174.01	6	3	-0.97	-0.04	111.9	4	No	No	0	0
Caffeic acid	180.04	4	3	1.15	-1.89	77.76	2	No	No	1	0
Vasicine	188.23	2	1	0.44	-1.6	35.83	0	No	No	0	0
Scopoletin	192.04	4	1	1.53	-2.46	59.67	1	No	No	0	0
Vasicinone	202.21	3	1	0.52	-1.91	55.12	0	No	No	0	0
Harmaline	214.11	2	1	2.07	-2.82	37.38	1	Yes	No	0	0
5,7-Dimethoxy-4-methylcoumarin	220.22	4	0	1.77	-2.65	48.67	2	No	No	0	0
3,4,5-Trihydroxystilbene	228.24	3	3	3.13	-3.62	60.69	2	No	Yes	1	0
Chrysin	254.05	4	2	3.52	-4.19	70.67	1	Yes	Yes	0	0
(*R*)-Semivioxanthin	274.08	5	2	3.3	-3.92	75.99	1	No	No	0	0
Biochanin A	284.06	5	2	2.99	-3.92	79.9	2	Yes	Yes	0	0
Ellagic acid	302.19	8	4	1.1	-2.94	141.34	0	No	No	1	0
Taxifolin	304.25	7	5	0.95	-2.66	127.45	1	No	No	1	0
Justicidin B	364.09	6	0	3.98	-4.85	63.22	3	Yes	Yes	0	0
Maruchantin E	440.16	5	4	5.41	-6.52	90.15	0	No	No	0	1
Emetine	480.29	6	1	4.74	-5.6	52.19	7	No	No	0	0
Pachymic acid	528.38	5	2	7.23	-7.15	83.83	8	No	Yes	0	3
Flavonol base + 4O, 1MeO, O-Hex-Hex, O-Hex	818.68	23	14	-3.28	-2.33	378.04	11	No	No	0	3
Licoricesaponin G2	838.94	17	9	1.58	-5.51	287.27	8	No	No	0	3
Procyanidin C1	866.20	18	15	3.31	-7.39	331.14	5	No	No	1	3

M.W: Molecular weight (< 500 g/mol); HBAs: Hydrogen bond acceptors (≤ 10); HBDs: Hydrogen bond donors (< 5); For lipophilicity: XLOGP3 between −0.7 and +5.0; For solubility: Log S between −6 and +0.5; ESOL: Estimated solubility; TPSA: Topological polar surface area (< 140 Å^2); Flexibility: no more than 9 rotatable bonds (RBs); PAINS: Pan assay interference compounds; Ro5: Lipinski's rule of five.

Table S3. Prediction of absorption, distribution, metabolism, excretion and toxicity (ADMET) properties of selected CLAE compounds by using pkCSM program.

Compound name	Absorption			Distribution		Metabolism		Excretion	Toxicity					
				Permeability		Substrates					Inhibitors			
	C2P	HIA (%)	P-gp sub.	BBB	CNS	CYP3A4	CYP2D6	Total clearance	AMES toxicity	Oral rat acute toxicity (LD_{50})	hERG I	hERG II	Hepato-toxicity	Skin sens.
Malic acid	-0.395	13.831	No	-0.788	-3.523	No	No	0.81	No	1.818	No	No	No	No
Vanillic acid	0.33	78.152	No	-0.38	-2.628	No	No	0.628	No	2.454	No	No	No	No
Gallic acid	-0.081	43.374	No	-1.102	-3.74	No	No	0.518	No	2.218	No	No	No	No
Syringic acid	0.495	73.076	Yes	-0.191	-2.701	No	No	0.646	No	2.157	No	No	No	No
3,4-Dimethoxycinnamic acid	1.225	94.767	No	0.502	-2.518	No	No	0.719	No	2.186	No	No	No	No
Capensine	1.103	94.652	Yes	-0.258	-2.855	No	No	0.764	No	2.112	No	No	No	No
Fustin	-0.174	73.643	Yes	-0.739	-3.111	No	No	-0.067	Yes	2.329	No	No	No	No
Enterolactone	1.045	93.934	Yes	-0.62	-2.383	Yes	Yes	0.161	No	2.02	No	No	No	No
Quercetin	-0.229	77.207	Yes	-1.098	-3.065	No	No	0.407	No	2.471	No	No	No	No
Epigallocatechin	-0.375	54.128	Yes	-1.377	-3.507	No	No	0.328	No	2.492	No	No	No	No
Andrographolide	1.07	95.357	No	-0.598	-2.691	Yes	Yes	1.183	No	2.162	No	Yes	No	No
14,15-Dehydro-16-epi-vincamine	0.946	90.873	Yes	0.74	-2.127	Yes	Yes	0.933	No	3.098	No	No	Yes	No
Otosenine	0.639	83.228	Yes	-0.701	-3.053	No	No	0.627	Yes	3.913	No	Yes	Yes	No
Voacristine	1.02	95.72	Yes	-0.67	-2.515	Yes	Yes	1.088	No	3.017	No	No	No	No
1-*O*-Sinapoylglucose	0.172	47.505	Yes	-1.308	-4.05	No	No	0.71	No	1.823	No	No	No	No
Scaposin	1.274	94.64	Yes	-1.546	-3.643	No	No	0.575	No	2.316	No	Yes	No	No
Kaempferol-3-*O*-arabinoside	0.128	59.181	Yes	-1.243	-4.053	No	No	0.431	No	2.607	No	Yes	No	No
Isoangustone A	-0.116	89.78	Yes	-1.025	-1.929	Yes	Yes	0.259	No	2.557	No	No	No	No
Gancaonin E	0.237	90.127	Yes	-0.875	-2.665	Yes	Yes	0.088	No	2.408	No	No	No	No
Naringenin-7-*O*-glucoside	0.415	36.035	Yes	-1.261	-4.053	No	No	0.378	No	2.683	No	No	No	No
Isoorientin	-0.912	61.768	Yes	-1.564	-3.939	No	No	0.372	No	2.55	No	Yes	No	No
Isosilybin A	0.43	69.767	Yes	-1.181	-3.671	No	No	-0.106	No	2.557	No	No	Yes	No
Harringtonine	0.601	71.074	Yes	-1.102	-3.585	Yes	Yes	1.299	No	2.017	No	No	No	No
Benzoic acid	1.707	100	No	-0.22	-2.002	No	No	0.707	No	2.17	No	No	No	No

Compound name	Absorption			Distribution		Metabolism		Excretion	Toxicity					
	C2P	HIA (%)	P-gp sub.	Permeability BBB	CNS	Substrates CYP3A4	CYP2D6	Total clearance	AMES toxicity	Oral rat acute toxicity (LD_{50})	Inhibitors hERG I	hERG II	Hepato-toxicity	Skin sens.
4-Aminobenzoic acid	0.558	81.966	No	-0.389	-3.222	No	No	0.492	No	1.548	No	No	No	No
p-Coumaraldehyde	1.725	92.221	No	0.445	-1.834	No	No	0.16	No	1.943	No	No	No	Yes
3-Indoleacetonitrile	1.491	92.011	No	0.381	-1.814	No	No	0.529	Yes	2.339	No	No	Yes	Yes
4-Hydroxycoumarin	1.206	94.551	No	-0.278	-2.741	No	No	0.751	No	2.047	No	No	No	No
cis-Aconitic acid	-0.274	0	No	-0.886	-3.156	No	No	0.99	No	2.138	No	No	No	No
Caffeic acid	0.634	69.407	No	-0.647	-2.608	No	No	0.508	No	2.383	No	No	No	No
Vasicine	1.595	86.22	No	-0.127	-2.159	Yes	Yes	0.58	Yes	2.697	No	No	No	No
Scopoletin	1.184	95.277	No	-0.299	-2.32	No	No	0.73	No	1.95	No	No	No	No
Vasicinone	1.172	92.532	Yes	-0.206	-2.323	No	No	0.568	No	1.91	No	No	No	No
Harmaline	1.621	93.622	Yes	0.39	-2.017	No	No	0.576	No	2.452	No	No	No	No
5,7-Dimethoxy-4-methylcoumarin	1.298	96.974	Yes	0.396	-2.289	No	No	0.831	No	2.001	No	No	No	No
3,4,5-Trihydroxystilbene	1.197	90.975	Yes	0.5	-2.075	Yes	Yes	-0.014	Yes	2.486	No	No	No	No
Chrysin	0.945	93.761	Yes	0.047	-1.912	No	No	0.405	No	2.289	No	No	No	No
(*R*)-Semivioxanthin	1.101	92.346	Yes	0.079	-2.169	No	No	0.49	No	1.737	No	No	No	No
Biochanin A	0.897	93.028	Yes	-0.221	-2.115	Yes	Yes	0.247	No	1.851	No	No	No	No
Ellagic acid	0.335	86.684	Yes	-1.272	-3.533	No	No	0.537	No	2.399	No	No	No	No
Taxifolin	0.924	64.709	Yes	-0.725	-3.198	No	No	-0.078	No	2.261	No	Yes	No	No
Justicidin B	1.151	99.578	No	-0.728	-2.876	Yes	Yes	0.34	Yes	2.843	No	Yes	Yes	No
Maruchantin E	0.667	97.775	Yes	-0.826	-1.469	Yes	Yes	-0.125	Yes	2.59	No	Yes	No	No
Emetine	0.751	91.032	Yes	-0.394	-2.067	Yes	Yes	0.993	No	2.793	No	No	No	No
Pachymic acid	0.694	97.531	No	-0.293	-1.748	Yes	Yes	0.246	No	2.284	No	Yes	No	No
Flavonol base + 4O, 1MeO, O-Hex-Hex, O-Hex	-1.116	0	Yes	-3.22	-6.434	No	No	-0.577	No	2.482	No	No	No	No
Licoricesaponin G2	-0.83	0	Yes	-1.644	-4.613	No	No	-0.194	No	2.481	No	Yes	No	No
Procyanidin C1	-1.826	60.469	Yes	-2.843	-4.855	No	No	-3.326	No	2.482	No	Yes	No	No

C2P: Caco-2 cell line permeability (log Papp value in 10^{-6} cm/s, > 0.90 represents higher permeability); HIA: Human intestinal absorption (< 30% is poorly absorbed); P-gp sub.: P-glycoprotein substrate; BBB: Blood brain barrier (expressed in log BB, logBB value > 0.3 indicates that a compound is considered to be readily cross the blood brain barrier); CNS: Central nervous system permeability is stated in terms of logPS (a compound with logPS > −2 is considered to penetrate the CNS); The predicted total

clearance is expressed as log(CL_{tot}) which is measured in log(mL/min/kg); AMES toxicity is the measurement of compound's mutagenic potential using bacteria; Oral rat acute toxicity values are calculated in terms of LD_{50} (lethal dose, 50%) and its unit is mol/kg; hERG: human ether-a-go-go related gene; Skin sens.: Skin sensitization.

Table S4. Myocardial infarction related pathogenic genes for CLAE.

No.	Symbol	Description	Category	Matched phenotypes	Matched phenotypes count	-LOG10(P)	Average disease-causing likelihood
1.	ACE	Angiotensin I Converting Enzyme	Protein Coding	Myocardial Infarction; Cardiac Hypertrophy; Acute Myocardial Infarction	3	4.47	15.82%
2.	MIAT	Myocardial Infarction Associated Transcript	RNA Gene	Myocardial Infarction; Cardiac Hypertrophy; Acute Myocardial Infarction	3	4.17	0.00%
3.	MEF2A	Myocyte Enhancer Factor 2A	Protein Coding	Myocardial Infarction; Cardiac Hypertrophy; Acute Myocardial Infarction	3	3.87	68.17%
4.	ITGB3	Integrin Subunit Beta 3	Protein Coding	Myocardial Infarction; Cardiac Hypertrophy; Acute Myocardial Infarction	3	3.63	58.75%
5.	CRP	C-Reactive Protein	Protein Coding	Myocardial Infarction; Cardiac Hypertrophy; Acute Myocardial Infarction	3	3.57	61.71%
6.	PLAT	Plasminogen Activator; Tissue Type	Protein Coding	Myocardial Infarction; Acute Myocardial Infarction	2	4.00	53.97%
7.	NPPB	Natriuretic Peptide B	Protein Coding	Myocardial Infarction; Cardiac Hypertrophy; Acute Myocardial Infarction	3	3.52	46.17%
8.	APOB	Apolipoprotein B	Protein Coding	Myocardial Infarction; Cardiac Hypertrophy; Acute Myocardial Infarction	3	3.47	41.09%
9.	MIR499A	MicroRNA 499a	RNA Gene	Myocardial Infarction; Cardiac Hypertrophy; Acute Myocardial Infarction	3	3.43	0.00%
10.	MIR208B	MicroRNA 208b	RNA Gene	Myocardial Infarction; Cardiac Hypertrophy; Acute Myocardial Infarction	3	3.33	0.00%
11.	OLR1	Oxidized Low Density Lipoprotein Receptor 1	Protein Coding	Myocardial Infarction; Acute Myocardial Infarction	2	3.70	42.84%
12.	IL6	Interleukin 6	Protein Coding	Myocardial Infarction; Cardiac Hypertrophy; Acute Myocardial Infarction	3	3.22	56.07%
13.	TNNT2	Troponin T2; Cardiac Type	Protein Coding	Myocardial Infarction; Cardiac Hypertrophy; Acute Myocardial Infarction	3	3.19	46.22%

No.	Symbol	Description	Category	Matched phenotypes	Matched phenotypes count	-LOG10(P)	Average disease-causing likelihood
14.	NPPA	Natriuretic Peptide A	Protein Coding	Myocardial Infarction; Cardiac Hypertrophy; Acute Myocardial Infarction	3	3.17	21.30%
15.	SERPINE1	Serpin Family E Member 1	Protein Coding	Myocardial Infarction; Cardiac Hypertrophy; Acute Myocardial Infarction	3	3.15	47.40%
16.	APOE	Apolipoprotein E	Protein Coding	Myocardial Infarction; Cardiac Hypertrophy; Acute Myocardial Infarction	3	3.13	30.00%
17.	TNNI3	Troponin I3; Cardiac Type	Protein Coding	Myocardial Infarction; Cardiac Hypertrophy; Acute Myocardial Infarction	3	3.11	58.63%
18.	AGTR1	Angiotensin II Receptor Type 1	Protein Coding	Myocardial Infarction; Cardiac Hypertrophy; Acute Myocardial Infarction	3	3.09	63.82%
19.	LTA	Lymphotoxin Alpha	Protein Coding	Myocardial Infarction; Acute Myocardial Infarction	2	3.39	12.09%
20.	APOA1	Apolipoprotein A1	Protein Coding	Myocardial Infarction; Cardiac Hypertrophy; Acute Myocardial Infarction	3	3.06	70.10%
21.	F7	Coagulation Factor VII	Protein Coding	Myocardial Infarction; Acute Myocardial Infarction	2	3.36	36.95%
22.	ESR1	Estrogen Receptor 1	Protein Coding	Myocardial Infarction; Cardiac Hypertrophy; Acute Myocardial Infarction	3	3.04	68.53%
23.	EDN1	Endothelin 1	Protein Coding	Myocardial Infarction; Cardiac Hypertrophy; Acute Myocardial Infarction	3	3.03	32.66%
24.	MMP9	Matrix Metallopeptidase 9	Protein Coding	Myocardial Infarction; Cardiac Hypertrophy; Acute Myocardial Infarction	3	3.01	20.28%
25.	F13A1	Coagulation Factor XIII A Chain	Protein Coding	Myocardial Infarction; Acute Myocardial Infarction	2	3.30	33.82%
26.	MB	Myoglobin	Protein Coding	Myocardial Infarction; Acute Myocardial Infarction	2	3.27	77.88%
27.	THBD	Thrombomodulin	Protein	Myocardial Infarction; Acute Myocardial Infarction	2	3.24	26.24%

No.	Symbol	Description	Category	Matched phenotypes	Matched phenotypes count	-LOG10(P)	Average disease-causing likelihood
			Coding				
28.	IL10	Interleukin 10	Protein Coding	Myocardial Infarction; Cardiac Hypertrophy; Acute Myocardial Infarction	3	2.94	63.14%
29.	LRP6	LDL Receptor Related Protein 6	Protein Coding	Myocardial Infarction; Cardiac Hypertrophy; Acute Myocardial Infarction	3	2.88	61.60%
30.	TNF	Tumor Necrosis Factor	Protein Coding	Myocardial Infarction; Cardiac Hypertrophy; Acute Myocardial Infarction	3	2.87	70.41%
31.	NOS3	Nitric Oxide Synthase 3	Protein Coding	Myocardial Infarction; Cardiac Hypertrophy; Acute Myocardial Infarction	3	2.86	58.50%
32.	FABP3	Fatty Acid Binding Protein 3	Protein Coding	Myocardial Infarction; Cardiac Hypertrophy; Acute Myocardial Infarction	3	2.79	52.95%
33.	PON1	Paraoxonase 1	Protein Coding	Myocardial Infarction; Cardiac Hypertrophy; Acute Myocardial Infarction	3	2.76	14.02%
34.	AGT	Angiotensinogen	Protein Coding	Myocardial Infarction; Cardiac Hypertrophy; Acute Myocardial Infarction	3	2.75	20.68%
35.	F3	Coagulation Factor III; Tissue Factor	Protein Coding	Myocardial Infarction; Acute Myocardial Infarction	2	3.00	45.38%
36.	LRP8	LDL Receptor Related Protein 8	Protein Coding	Myocardial Infarction; Acute Myocardial Infarction	2	2.98	50.12%
37.	LPL	Lipoprotein Lipase	Protein Coding	Myocardial Infarction; Acute Myocardial Infarction	2	2.92	25.04%
38.	ITGA2B	Integrin Subunit Alpha 2b	Protein Coding	Myocardial Infarction; Cardiac Hypertrophy; Acute Myocardial Infarction	3	2.70	50.67%
39.	ADIPOQ	Adiponectin; C1Q And Collagen Domain Containing	Protein Coding	Myocardial Infarction; Cardiac Hypertrophy; Acute Myocardial Infarction	3	2.70	38.10%
40.	SELP	Selectin P	Protein Coding	Myocardial Infarction; Acute Myocardial Infarction	2	2.91	14.14%

No.	Symbol	Description	Category	Matched phenotypes	Matched phenotypes count	-LOG10(P)	Average disease-causing likelihood
41.	JAK2	Janus Kinase 2	Protein Coding	Myocardial Infarction; Cardiac Hypertrophy; Acute Myocardial Infarction	3	2.69	71.65%
42.	VEGFA	Vascular Endothelial Growth Factor A	Protein Coding	Myocardial Infarction; Cardiac Hypertrophy; Acute Myocardial Infarction	3	2.68	64.83%
43.	CETP	Cholesteryl Ester Transfer Protein	Protein Coding	Myocardial Infarction; Cardiac Hypertrophy; Acute Myocardial Infarction	3	2.65	43.59%
44.	VWF	Von Willebrand Factor	Protein Coding	Myocardial Infarction; Acute Myocardial Infarction	2	2.85	1.01%
45.	F2	Coagulation Factor II; Thrombin	Protein Coding	Myocardial Infarction; Acute Myocardial Infarction	2	2.83	56.27%
46.	MMP3	Matrix Metallopeptidase 3	Protein Coding	Myocardial Infarction; Cardiac Hypertrophy; Acute Myocardial Infarction	3	2.64	52.50%
47.	PCSK9	Proprotein Convertase Subtilisin/Kexin Type 9	Protein Coding	Myocardial Infarction; Acute Myocardial Infarction	2	2.82	15.35%
48.	MIR210	MicroRNA 210	RNA Gene	Myocardial Infarction; Acute Myocardial Infarction	2	2.80	0.00%
49.	F5	Coagulation Factor V	Protein Coding	Myocardial Infarction; Acute Myocardial Infarction	2	2.78	3.19%
50.	LDLR	Low Density Lipoprotein Receptor	Protein Coding	Myocardial Infarction; Acute Myocardial Infarction	2	2.77	58.01%
51.	TNFSF4	TNF Superfamily Member 4	Protein Coding	Myocardial Infarction; Acute Myocardial Infarction	2	2.77	71.26%
52.	REN	Renin	Protein Coding	Myocardial Infarction; Cardiac Hypertrophy; Acute Myocardial Infarction	3	2.62	75.65%
53.	TGFB1	Transforming Growth Factor Beta 1	Protein Coding	Myocardial Infarction; Cardiac Hypertrophy; Acute Myocardial Infarction	3	2.62	42.49%
54.	SERPINC1	Serpin Family C Member 1	Protein	Myocardial Infarction; Acute Myocardial Infarction	2	2.74	60.97%

No.	Symbol	Description	Category	Matched phenotypes	Matched phenotypes count	-LOG10(P)	Average disease-causing likelihood
			Coding				
55.	HFE	Homeostatic Iron Regulator	Protein Coding	Myocardial Infarction; Cardiac Hypertrophy; Acute Myocardial Infarction	3	2.60	22.09%
56.	ADM	Adrenomedullin	Protein Coding	Myocardial Infarction; Cardiac Hypertrophy; Acute Myocardial Infarction	3	2.59	45.97%
57.	CDKN2B-AS1	CDKN2B Antisense RNA 1	RNA Gene	Myocardial Infarction; Cardiac Hypertrophy; Acute Myocardial Infarction	3	2.59	0.00%
58.	MTHFR	Methylenetetrahydrofolate Reductase	Protein Coding	Myocardial Infarction; Acute Myocardial Infarction	2	2.73	22.21%
59.	TLR4	Toll Like Receptor 4	Protein Coding	Myocardial Infarction; Acute Myocardial Infarction	2	2.71	25.45%
60.	LGALS2	Galectin 2	Protein Coding	Myocardial Infarction	1	3.08	27.24%
61.	MMP2	Matrix Metallopeptidase 2	Protein Coding	Myocardial Infarction; Cardiac Hypertrophy; Acute Myocardial Infarction	3	2.57	71.78%
62.	MPO	Myeloperoxidase	Protein Coding	Myocardial Infarction; Cardiac Hypertrophy; Acute Myocardial Infarction	3	2.56	33.79%
63.	PTGS2	Prostaglandin-Endoperoxide Synthase 2	Protein Coding	Myocardial Infarction; Cardiac Hypertrophy; Acute Myocardial Infarction	3	2.55	66.54%
64.	PPARG	Peroxisome Proliferator Activated Receptor Gamma	Protein Coding	Myocardial Infarction; Cardiac Hypertrophy; Acute Myocardial Infarction	3	2.54	58.38%
65.	ITGA2	Integrin Subunit Alpha 2	Protein Coding	Myocardial Infarction; Cardiac Hypertrophy; Acute Myocardial Infarction	3	2.52	45.90%
66.	IL1B	Interleukin 1 Beta	Protein Coding	Myocardial Infarction; Cardiac Hypertrophy; Acute Myocardial Infarction	3	2.51	81.84%
67.	IGF1	Insulin Like Growth Factor 1	Protein Coding	Myocardial Infarction; Cardiac Hypertrophy; Acute Myocardial Infarction	3	2.50	50.87%

No.	Symbol	Description	Category	Matched phenotypes	Matched phenotypes count	-LOG10(P)	Average disease-causing likelihood
68.	LPA	Lipoprotein(A)	Protein Coding	Myocardial Infarction; Acute Myocardial Infarction	2	2.67	3.20%
69.	FGA	Fibrinogen Alpha Chain	Protein Coding	Myocardial Infarction; Acute Myocardial Infarction	2	2.66	63.13%
70.	MYH7	Myosin Heavy Chain 7	Protein Coding	Myocardial Infarction; Cardiac Hypertrophy; Acute Myocardial Infarction	3	2.50	79.57%
71.	ICAM1	Intercellular Adhesion Molecule 1	Protein Coding	Myocardial Infarction; Cardiac Hypertrophy; Acute Myocardial Infarction	3	2.49	20.08%
72.	ADRB1	Adrenoceptor Beta 1	Protein Coding	Myocardial Infarction; Cardiac Hypertrophy; Acute Myocardial Infarction	3	2.48	63.76%
73.	IL18	Interleukin 18	Protein Coding	Myocardial Infarction; Cardiac Hypertrophy; Acute Myocardial Infarction	3	2.48	64.91%
74.	ALB	Albumin	Protein Coding	Myocardial Infarction; Cardiac Hypertrophy; Acute Myocardial Infarction	3	2.47	82.33%
75.	VCAM1	Vascular Cell Adhesion Molecule 1	Protein Coding	Myocardial Infarction; Cardiac Hypertrophy; Acute Myocardial Infarction	3	2.47	53.48%
76.	SELE	Selectin E	Protein Coding	Myocardial Infarction; Acute Myocardial Infarction	2	2.65	29.04%
77.	INS	Insulin	Protein Coding	Myocardial Infarction; Cardiac Hypertrophy; Acute Myocardial Infarction	3	2.46	74.53%
78.	CKB	Creatine Kinase B	Protein Coding	Myocardial Infarction; Cardiac Hypertrophy; Acute Myocardial Infarction	3	2.46	69.75%
79.	MIR21	MicroRNA 21	RNA Gene	Myocardial Infarction; Cardiac Hypertrophy; Acute Myocardial Infarction	3	2.46	0.00%
80.	PECAM1	Platelet And Endothelial Cell Adhesion Molecule 1	Protein Coding	Myocardial Infarction; Acute Myocardial Infarction	2	2.63	0.00%
81.	CTNNB1	Catenin Beta 1	Protein	Myocardial Infarction; Cardiac Hypertrophy; Acute	3	2.45	91.86%

No.	Symbol	Description	Category	Matched phenotypes	Matched phenotypes count	-LOG10(P)	Average disease-causing likelihood
			Coding	Myocardial Infarction			
82.	PSMA6	Proteasome 20S Subunit Alpha 6	Protein Coding	Myocardial Infarction	1	2.97	82.23%
83.	ADRB2	Adrenoceptor Beta 2	Protein Coding	Myocardial Infarction; Cardiac Hypertrophy; Acute Myocardial Infarction	3	2.44	41.04%
84.	MMP1	Matrix Metallopeptidase 1	Protein Coding	Myocardial Infarction; Cardiac Hypertrophy; Acute Myocardial Infarction	3	2.44	34.17%
85.	TP53	Tumor Protein P53	Protein Coding	Myocardial Infarction; Cardiac Hypertrophy; Acute Myocardial Infarction	3	2.42	72.85%
86.	AGER	Advanced Glycosylation End-Product Specific Receptor	Protein Coding	Myocardial Infarction; Cardiac Hypertrophy; Acute Myocardial Infarction	3	2.42	27.13%
87.	ELN	Elastin	Protein Coding	Myocardial Infarction; Cardiac Hypertrophy; Acute Myocardial Infarction	3	2.41	28.69%
88.	GCLC	Glutamate-Cysteine Ligase Catalytic Subunit	Protein Coding	Myocardial Infarction	1	2.95	68.34%
89.	ADAMTS13	ADAM Metallopeptidase with Thrombospondin Type 1 Motif 13	Protein Coding	Myocardial Infarction; Acute Myocardial Infarction	2	2.61	9.02%
90.	APOC3	Apolipoprotein C3	Protein Coding	Myocardial Infarction; Cardiac Hypertrophy; Acute Myocardial Infarction	3	2.40	58.93%
91.	CXCL12	C-X-C Motif Chemokine Ligand 12	Protein Coding	Myocardial Infarction; Acute Myocardial Infarction	2	2.60	61.77%
92.	AGTR2	Angiotensin II Receptor Type 2	Protein Coding	Myocardial Infarction; Cardiac Hypertrophy; Acute Myocardial Infarction	3	2.40	41.73%
93.	CBS	Cystathionine Beta-Synthase	Protein Coding	Myocardial Infarction; Cardiac Hypertrophy; Acute Myocardial Infarction	3	2.39	54.73%
94.	GCLM	Glutamate-Cysteine Ligase	Protein	Myocardial Infarction	1	2.89	78.79%

No.	Symbol	Description	Category	Matched phenotypes	Matched phenotypes count	-LOG10(P)	Average disease-causing likelihood
		Modifier Subunit	Coding				
95.	GATA4	GATA Binding Protein 4	Protein Coding	Myocardial Infarction; Cardiac Hypertrophy; Acute Myocardial Infarction	3	2.39	33.78%
96.	CD14	CD14 Molecule	Protein Coding	Myocardial Infarction; Acute Myocardial Infarction	2	2.58	66.35%
97.	NR3C2	Nuclear Receptor Subfamily 3 Group C Member 2	Protein Coding	Myocardial Infarction; Cardiac Hypertrophy; Acute Myocardial Infarction	3	2.38	63.80%
98.	ABCA1	ATP Binding Cassette Subfamily A Member 1	Protein Coding	Myocardial Infarction; Acute Myocardial Infarction	2	2.58	51.73%
99.	CCL2	C-C Motif Chemokine Ligand 2	Protein Coding	Myocardial Infarction; Acute Myocardial Infarction	2	2.56	69.23%
100.	PTX3	Pentraxin 3	Protein Coding	Myocardial Infarction; Acute Myocardial Infarction	2	2.55	30.68%
101.	PPARA	Peroxisome Proliferator Activated Receptor Alpha	Protein Coding	Myocardial Infarction; Cardiac Hypertrophy; Acute Myocardial Infarction	3	2.38	36.99%
102.	LGALS3	Galectin 3	Protein Coding	Myocardial Infarction; Cardiac Hypertrophy; Acute Myocardial Infarction	3	2.37	22.69%
103.	CYP2C19	Cytochrome P450 Family 2 Subfamily C Member 19	Protein Coding	Myocardial Infarction; Acute Myocardial Infarction	2	2.53	21.81%
104.	CKM	Creatine Kinase; M-Type	Protein Coding	Myocardial Infarction; Acute Myocardial Infarction	2	2.52	71.23%
105.	RETN	Resistin	Protein Coding	Myocardial Infarction; Cardiac Hypertrophy; Acute Myocardial Infarction	3	2.36	75.34%
106.	FGB	Fibrinogen Beta Chain	Protein Coding	Myocardial Infarction; Acute Myocardial Infarction	2	2.51	42.36%
107.	GUCY1A1	Guanylate Cyclase 1 Soluble Subunit Alpha 1	Protein Coding	Myocardial Infarction	1	2.84	62.09%

No.	Symbol	Description	Category	Matched phenotypes	Matched phenotypes count	-LOG10(P)	Average disease-causing likelihood
108.	PON2	Paraoxonase 2	Protein Coding	Myocardial Infarction; Cardiac Hypertrophy; Acute Myocardial Infarction	3	2.36	33.29%
109.	GNB3	G Protein Subunit Beta 3	Protein Coding	Myocardial Infarction; Cardiac Hypertrophy; Acute Myocardial Infarction	3	2.35	65.39%
110.	KNG1	Kininogen 1	Protein Coding	Myocardial Infarction; Cardiac Hypertrophy; Acute Myocardial Infarction	3	2.34	39.10%
111.	CSF3	Colony Stimulating Factor 3	Protein Coding	Myocardial Infarction; Cardiac Hypertrophy; Acute Myocardial Infarction	3	2.34	42.61%
112.	ALDH2	Aldehyde Dehydrogenase 2 Family Member	Protein Coding	Myocardial Infarction; Cardiac Hypertrophy; Acute Myocardial Infarction	3	2.32	51.06%
113.	LIPC	Lipase C; Hepatic Type	Protein Coding	Myocardial Infarction; Cardiac Hypertrophy; Acute Myocardial Infarction	3	2.32	40.73%
114.	HMOX1	Heme Oxygenase 1	Protein Coding	Myocardial Infarction; Cardiac Hypertrophy; Acute Myocardial Infarction	3	2.32	31.92%
115.	AVP	Arginine Vasopressin	Protein Coding	Myocardial Infarction; Acute Myocardial Infarction	2	2.49	0.00%
116.	IFNG	Interferon Gamma	Protein Coding	Myocardial Infarction; Cardiac Hypertrophy; Acute Myocardial Infarction	3	2.31	81.00%
117.	PF4	Platelet Factor 4	Protein Coding	Myocardial Infarction; Acute Myocardial Infarction	2	2.45	71.14%
118.	PAPPA	Pappalysin 1	Protein Coding	Myocardial Infarction; Acute Myocardial Infarction	2	2.44	60.82%
119.	PLG	Plasminogen	Protein Coding	Myocardial Infarction; Acute Myocardial Infarction	2	2.43	38.18%
120.	CXCL8	C-X-C Motif Chemokine Ligand 8	Protein Coding	Myocardial Infarction; Acute Myocardial Infarction	2	2.43	70.13%
121.	CLCN1	Chloride Voltage-Gated	Protein	Myocardial Infarction	1	2.72	36.16%

No.	Symbol	Description	Category	Matched phenotypes	Matched phenotypes count	-LOG10(P)	Average disease-causing likelihood
		Channel 1	Coding				
122.	MIR29A	MicroRNA 29a	RNA Gene	Myocardial Infarction; Cardiac Hypertrophy; Acute Myocardial Infarction	3	2.26	0.00%
123.	HP	Haptoglobin	Protein Coding	Myocardial Infarction; Acute Myocardial Infarction	2	2.41	32.66%
124.	BDKRB2	Bradykinin Receptor B2	Protein Coding	Myocardial Infarction; Cardiac Hypertrophy; Acute Myocardial Infarction	3	2.26	39.75%
125.	GJA4	Gap Junction Protein Alpha 4	Protein Coding	Myocardial Infarction; Acute Myocardial Infarction	2	2.41	23.73%
126.	TLR2	Toll Like Receptor 2	Protein Coding	Myocardial Infarction; Cardiac Hypertrophy; Acute Myocardial Infarction	3	2.25	33.06%
127.	HMGB1	High Mobility Group Box 1	Protein Coding	Myocardial Infarction; Cardiac Hypertrophy; Acute Myocardial Infarction	3	2.24	64.54%
128.	TIMP1	TIMP Metallopeptidase Inhibitor 1	Protein Coding	Myocardial Infarction; Cardiac Hypertrophy; Acute Myocardial Infarction	3	2.24	13.68%
129.	MIR199A1	MicroRNA 199a-1	RNA Gene	Myocardial Infarction; Cardiac Hypertrophy; Acute Myocardial Infarction	3	2.23	0.00%
130.	MIR22	MicroRNA 22	RNA Gene	Myocardial Infarction; Cardiac Hypertrophy; Acute Myocardial Infarction	3	2.23	0.00%
131.	ITGB1	Integrin Subunit Beta 1	Protein Coding	Myocardial Infarction; Cardiac Hypertrophy; Acute Myocardial Infarction	3	2.23	87.68%
132.	ACE2	Angiotensin Converting Enzyme 2	Protein Coding	Myocardial Infarction; Cardiac Hypertrophy; Acute Myocardial Infarction	3	2.22	59.57%
133.	HIF1A	Hypoxia Inducible Factor 1 Subunit Alpha	Protein Coding	Myocardial Infarction; Cardiac Hypertrophy; Acute Myocardial Infarction	3	2.22	61.97%
134.	MIR146A	MicroRNA 146a	RNA Gene	Myocardial Infarction; Cardiac Hypertrophy; Acute Myocardial Infarction	3	2.22	0.00%

No.	Symbol	Description	Category	Matched phenotypes	Matched phenotypes count	-LOG10(P)	Average disease-causing likelihood
135.	IL17A	Interleukin 17A	Protein Coding	Myocardial Infarction; Cardiac Hypertrophy; Acute Myocardial Infarction	3	2.22	61.60%
136.	STAT3	Signal Transducer and Activator Of Transcription 3	Protein Coding	Myocardial Infarction; Cardiac Hypertrophy; Acute Myocardial Infarction	3	2.21	78.94%
137.	MEF2C	Myocyte Enhancer Factor 2C	Protein Coding	Myocardial Infarction; Cardiac Hypertrophy; Acute Myocardial Infarction	3	2.21	87.81%
138.	NPY	Neuropeptide Y	Protein Coding	Myocardial Infarction; Cardiac Hypertrophy; Acute Myocardial Infarction	3	2.20	51.25%
139.	HTR2A	5-Hydroxytryptamine Receptor 2A	Protein Coding	Myocardial Infarction; Cardiac Hypertrophy; Acute Myocardial Infarction	3	2.20	62.08%
140.	CTF1	Cardiotrophin 1	Protein Coding	Myocardial Infarction; Cardiac Hypertrophy; Acute Myocardial Infarction	3	2.19	0.00%
141.	F10	Coagulation Factor X	Protein Coding	Myocardial Infarction; Acute Myocardial Infarction	2	2.38	62.81%
142.	CD40LG	CD40 Ligand	Protein Coding	Myocardial Infarction; Acute Myocardial Infarction	2	2.37	63.35%
143.	SERPINA3	Serpin Family A Member 3	Protein Coding	Myocardial Infarction; Acute Myocardial Infarction	2	2.37	20.59%
144.	CYP11B2	Cytochrome P450 Family 11 Subfamily B Member 2	Protein Coding	Myocardial Infarction; Cardiac Hypertrophy; Acute Myocardial Infarction	3	2.18	33.11%
145.	IL1RN	Interleukin 1 Receptor Antagonist	Protein Coding	Myocardial Infarction; Acute Myocardial Infarction	2	2.36	79.13%
146.	FAS	Fas Cell Surface Death Receptor	Protein Coding	Myocardial Infarction; Cardiac Hypertrophy; Acute Myocardial Infarction	3	2.17	44.27%
147.	ANXA5	Annexin A5	Protein Coding	Myocardial Infarction; Cardiac Hypertrophy; Acute Myocardial Infarction	3	2.16	59.65%
148.	MIR208A	MicroRNA 208a	RNA	Myocardial Infarction; Cardiac Hypertrophy; Acute	3	2.16	0.00%

No.	Symbol	Description	Category	Matched phenotypes	Matched phenotypes count	-LOG10(P)	Average disease-causing likelihood
			Gene	Myocardial Infarction			
149.	CA3	Carbonic Anhydrase 3	Protein Coding	Myocardial Infarction; Acute Myocardial Infarction	2	2.35	32.84%
150.	EPO	Erythropoietin	Protein Coding	Myocardial Infarction; Cardiac Hypertrophy; Acute Myocardial Infarction	3	2.16	50.01%
151.	GP1BA	Glycoprotein Ib Platelet Subunit Alpha	Protein Coding	Myocardial Infarction; Acute Myocardial Infarction	2	2.34	46.66%
152.	LEP	Leptin	Protein Coding	Myocardial Infarction; Acute Myocardial Infarction	2	2.33	59.73%
153.	THBS4	Thrombospondin 4	Protein Coding	Myocardial Infarction; Cardiac Hypertrophy; Acute Myocardial Infarction	3	2.15	41.35%
154.	MIR150	MicroRNA 150	RNA Gene	Myocardial Infarction; Acute Myocardial Infarction	2	2.33	0.00%
155.	HGF	Hepatocyte Growth Factor	Protein Coding	Myocardial Infarction; Acute Myocardial Infarction	2	2.33	70.64%
156.	SLC6A4	Solute Carrier Family 6 Member 4	Protein Coding	Myocardial Infarction; Cardiac Hypertrophy; Acute Myocardial Infarction	3	2.15	72.97%
157.	CD36	CD36 Molecule	Protein Coding	Myocardial Infarction; Cardiac Hypertrophy; Acute Myocardial Infarction	3	2.15	20.40%
158.	THBS1	Thrombospondin 1	Protein Coding	Myocardial Infarction; Acute Myocardial Infarction	2	2.31	43.29%
159.	CYP2C9	Cytochrome P450 Family 2 Subfamily C Member 9	Protein Coding	Myocardial Infarction; Acute Myocardial Infarction	2	2.31	18.56%
160.	MIR223	MicroRNA 223	RNA Gene	Myocardial Infarction; Acute Myocardial Infarction	2	2.30	0.00%
161.	APLN	Apelin	Protein Coding	Myocardial Infarction; Cardiac Hypertrophy; Acute Myocardial Infarction	3	2.13	0.00%

No.	Symbol	Description	Category	Matched phenotypes	Matched phenotypes count	-LOG10(P)	Average disease-causing likelihood
162.	CST3	Cystatin C	Protein Coding	Myocardial Infarction; Acute Myocardial Infarction	2	2.30	0.00%
163.	MIR126	MicroRNA 126	RNA Gene	Myocardial Infarction; Acute Myocardial Infarction	2	2.30	0.00%
164.	MIR132	MicroRNA 132	RNA Gene	Myocardial Infarction; Cardiac Hypertrophy; Acute Myocardial Infarction	3	2.13	0.00%
165.	NPR1	Natriuretic Peptide Receptor 1	Protein Coding	Myocardial Infarction; Cardiac Hypertrophy; Acute Myocardial Infarction	3	2.12	40.51%
166.	FLT1	Fms Related Receptor Tyrosine Kinase 1	Protein Coding	Myocardial Infarction; Cardiac Hypertrophy; Acute Myocardial Infarction	3	2.12	77.15%
167.	IL4	Interleukin 4	Protein Coding	Myocardial Infarction; Cardiac Hypertrophy; Acute Myocardial Infarction	3	2.12	68.48%
168.	CXCR4	C-X-C Motif Chemokine Receptor 4	Protein Coding	Myocardial Infarction; Cardiac Hypertrophy; Acute Myocardial Infarction	3	2.11	78.34%
169.	LCN2	Lipocalin 2	Protein Coding	Myocardial Infarction; Cardiac Hypertrophy; Acute Myocardial Infarction	3	2.11	42.06%
170.	FN1	Fibronectin 1	Protein Coding	Myocardial Infarction; Acute Myocardial Infarction	2	2.29	56.86%
171.	MIR34A	MicroRNA 34a	RNA Gene	Myocardial Infarction; Acute Myocardial Infarction	2	2.29	0.00%
172.	UTS2	Urotensin 2	Protein Coding	Myocardial Infarction; Cardiac Hypertrophy; Acute Myocardial Infarction	3	2.11	10.48%
173.	PROCR	Protein C Receptor	Protein Coding	Myocardial Infarction; Acute Myocardial Infarction	2	2.29	31.98%
174.	MIR145	MicroRNA 145	RNA	Myocardial Infarction; Acute Myocardial Infarction	2	2.28	0.00%

No.	Symbol	Description	Category	Matched phenotypes	Matched phenotypes count	-LOG10(P)	Average disease-causing likelihood
			Gene				
175.	ITGAM	Integrin Subunit Alpha M	Protein Coding	Myocardial Infarction; Cardiac Hypertrophy; Acute Myocardial Infarction	3	2.10	46.05%
176.	APOA2	Apolipoprotein A2	Protein Coding	Myocardial Infarction; Cardiac Hypertrophy; Acute Myocardial Infarction	3	2.10	77.05%
177.	CASP3	Caspase 3	Protein Coding	Myocardial Infarction; Cardiac Hypertrophy; Acute Myocardial Infarction	3	2.10	64.18%
178.	KCNQ1	Potassium Voltage-Gated Channel Subfamily Q Member 1	Protein Coding	Myocardial Infarction; Cardiac Hypertrophy; Acute Myocardial Infarction	3	2.10	73.58%
179.	POSTN	Periostin	Protein Coding	Myocardial Infarction; Acute Myocardial Infarction	2	2.28	56.11%
180.	CD40	CD40 Molecule	Protein Coding	Myocardial Infarction; Acute Myocardial Infarction	2	2.27	60.13%
181.	GDF15	Growth Differentiation Factor 15	Protein Coding	Myocardial Infarction; Acute Myocardial Infarction	2	2.27	41.31%
182.	APOA5	Apolipoprotein A5	Protein Coding	Myocardial Infarction; Acute Myocardial Infarction	2	2.27	25.94%
183.	SIRT1	Sirtuin 1	Protein Coding	Myocardial Infarction; Cardiac Hypertrophy; Acute Myocardial Infarction	3	2.09	73.01%
184.	IGFBP1	Insulin Like Growth Factor Binding Protein 1	Protein Coding	Myocardial Infarction; Acute Myocardial Infarction	2	2.26	50.90%
185.	CYBA	Cytochrome B-245 Alpha Chain	Protein Coding	Myocardial Infarction; Cardiac Hypertrophy; Acute Myocardial Infarction	3	2.08	0.00%
186.	APOH	Apolipoprotein H	Protein Coding	Myocardial Infarction; Acute Myocardial Infarction	2	2.25	25.92%
187.	P2RY12	Purinergic Receptor P2Y12	Protein Coding	Myocardial Infarction; Acute Myocardial Infarction	2	2.25	67.32%

No.	Symbol	Description	Category	Matched phenotypes	Matched phenotypes count	-LOG10(P)	Average disease-causing likelihood
188.	MYH6	Myosin Heavy Chain 6	Protein Coding	Myocardial Infarction; Cardiac Hypertrophy	2	2.25	44.00%
189.	AHSG	Alpha 2-HS Glycoprotein	Protein Coding	Myocardial Infarction; Cardiac Hypertrophy; Acute Myocardial Infarction	3	2.07	42.52%
190.	WRN	WRN RecQ Like Helicase	Protein Coding	Myocardial Infarction	1	2.50	13.62%
191.	NPPC	Natriuretic Peptide C	Protein Coding	Myocardial Infarction; Cardiac Hypertrophy; Acute Myocardial Infarction	3	2.06	45.81%
192.	CPB2	Carboxypeptidase B2	Protein Coding	Myocardial Infarction; Acute Myocardial Infarction	2	2.23	40.67%
193.	SELL	Selectin L	Protein Coding	Myocardial Infarction; Acute Myocardial Infarction	2	2.21	26.44%
194.	IL1A	Interleukin 1 Alpha	Protein Coding	Myocardial Infarction; Acute Myocardial Infarction	2	2.20	17.73%
195.	FGF2	Fibroblast Growth Factor 2	Protein Coding	Myocardial Infarction; Cardiac Hypertrophy; Acute Myocardial Infarction	3	2.05	65.39%
196.	HSPA4	Heat Shock Protein Family A (Hsp70) Member 4	Protein Coding	Myocardial Infarction; Cardiac Hypertrophy; Acute Myocardial Infarction	3	2.05	63.02%
197.	PTGS1	Prostaglandin-Endoperoxide Synthase 1	Protein Coding	Myocardial Infarction; Cardiac Hypertrophy; Acute Myocardial Infarction	3	2.04	25.27%
198.	ENG	Endoglin	Protein Coding	Myocardial Infarction; Acute Myocardial Infarction	2	2.19	58.11%
199.	TNFRSF1A	TNF Receptor Superfamily Member 1A	Protein Coding	Myocardial Infarction; Acute Myocardial Infarction	2	2.19	55.74%
200.	SOD1	Superoxide Dismutase 1	Protein Coding	Myocardial Infarction; Cardiac Hypertrophy; Acute Myocardial Infarction	3	2.04	77.48%

Table S5. Anxiety, depression and impaired memory related pathogenic genes for CLAE.

No.	Symbol	Description	Category	Matched phenotypes	Matched phenotypes count	-LOG10(P)	Average disease-causing likelihood
1.	SLC6A4	Solute Carrier Family 6 Member 4	Protein Coding	Anxiety Disorder; Anxiety Disease; Mental Depression; Memory Loss; Memory Impairment	5	4.47	72.97%
2.	HTR2A	5-Hydroxytryptamine Receptor 2A	Protein Coding	Anxiety Disorder; Anxiety Disease; Mental Depression; Memory Loss; Memory Impairment	5	3.87	62.08%
3.	BDNF	Brain Derived Neurotrophic Factor	Protein Coding	Anxiety Disorder; Anxiety Disease; Mental Depression; Memory Loss; Memory Impairment	5	3.63	36.81%
4.	APP	Amyloid Beta Precursor Protein	Protein Coding	Anxiety Disorder; Memory Loss; Memory Impairment	3	3.70	77.65%
5.	PRNP	Prion Protein	Protein Coding	Anxiety Disease; Memory Loss; Memory Impairment	3	3.57	29.89%
6.	GRN	Granulin Precursor	Protein Coding	Anxiety Disease; Mental Depression; Memory Loss; Memory Impairment	4	3.43	69.69%
7.	DRD2	Dopamine Receptor D2	Protein Coding	Anxiety Disorder; Anxiety Disease; Mental Depression; Memory Loss; Memory Impairment	5	3.17	63.33%
8.	OXT	Oxytocin/Neurophysin I Prepropeptide	Protein Coding	Anxiety Disorder; Mental Depression; Memory Loss; Memory Impairment	4	3.22	72.30%
9.	CHMP2B	Charged Multivesicular Body Protein 2B	Protein Coding	Anxiety Disease; Mental Depression; Memory Loss; Memory Impairment	4	3.13	63.84%
10.	TMEM106B	Transmembrane Protein 106B	Protein Coding	Anxiety Disorder; Anxiety Disease; Mental Depression; Memory Loss; Memory Impairment	5	3.08	70.63%
11.	DCTN1	Dynactin Subunit 1	Protein	Anxiety Disease; Mental Depression;	4	3.11	66.27%

No.	Symbol	Description	Category	Matched phenotypes	Matched phenotypes count	-LOG10(P)	Average disease-causing likelihood
			Coding	Memory Loss; Memory Impairment			
12.	CRHR1	Corticotropin Releasing Hormone Receptor 1	Protein Coding	Anxiety Disorder; Mental Depression; Memory Loss; Memory Impairment	4	3.09	78.05%
13.	VCP	Valosin Containing Protein	Protein Coding	Anxiety Disease; Mental Depression; Memory Loss; Memory Impairment	4	3.06	90.66%
14.	GRIN2A	Glutamate Ionotropic Receptor NMDA Type Subunit 2A	Protein Coding	Anxiety Disorder; Anxiety Disease; Mental Depression; Memory Loss; Memory Impairment	5	2.92	78.30%
15.	FMR1	Fragile X Messenger Ribonucleoprotein 1	Protein Coding	Anxiety Disorder; Anxiety Disease; Memory Loss; Memory Impairment	4	3.04	46.07%
16.	SLC6A3	Solute Carrier Family 6 Member 3	Protein Coding	Anxiety Disorder; Mental Depression; Memory Loss; Memory Impairment	4	3.03	84.90%
17.	POMC	Proopiomelanocortin	Protein Coding	Anxiety Disorder; Mental Depression; Memory Loss; Memory Impairment	4	3.00	32.82%
18.	TSPO	Translocator Protein	Protein Coding	Anxiety Disorder; Mental Depression; Memory Loss; Memory Impairment	4	2.94	0.00%
19.	SLC6A2	Solute Carrier Family 6 Member 2	Protein Coding	Anxiety Disorder; Mental Depression; Memory Loss	3	3.01	57.52%
20.	SNCA	Synuclein Alpha	Protein Coding	Anxiety Disorder; Anxiety Disease; Mental Depression; Memory Loss; Memory Impairment	5	2.65	65.02%
21.	AKT1	AKT Serine/Threonine Kinase 1	Protein Coding	Anxiety Disorder; Mental Depression; Memory Loss; Memory Impairment	4	2.82	90.77%
22.	TPH1	Tryptophan Hydroxylase 1	Protein Coding	Anxiety Disorder; Mental Depression; Memory Loss	3	2.97	54.69%
23.	H2AC18	H2A Clustered Histone 18	Protein Coding	Anxiety Disorder; Mental Depression; Memory Loss; Memory Impairment	4	2.76	0.00%

No.	Symbol	Description	Category	Matched phenotypes	Matched phenotypes count	-LOG10(P)	Average disease-causing likelihood
24.	IL1B	Interleukin 1 Beta	Protein Coding	Anxiety Disorder; Mental Depression; Memory Loss; Memory Impairment	4	2.75	81.84%
25.	DRD4	Dopamine Receptor D4	Protein Coding	Anxiety Disorder; Mental Depression; Memory Loss	3	2.89	0.00%
26.	HTR2C	5-Hydroxytryptamine Receptor 2C	Protein Coding	Anxiety Disorder; Mental Depression; Memory Loss	3	2.85	33.28%
27.	CREB1	CAMP Responsive Element Binding Protein 1	Protein Coding	Anxiety Disorder; Mental Depression; Memory Loss; Memory Impairment	4	2.63	63.02%
28.	PDGFB	Platelet Derived Growth Factor Subunit B	Protein Coding	Anxiety Disease; Memory Loss; Memory Impairment	3	2.80	54.61%
29.	TNF	Tumor Necrosis Factor	Protein Coding	Anxiety Disorder; Mental Depression; Memory Loss; Memory Impairment	4	2.61	70.41%
30.	INS	Insulin	Protein Coding	Anxiety Disorder; Mental Depression; Memory Loss; Memory Impairment	4	2.60	74.53%
31.	HCRT	Hypocretin Neuropeptide Precursor	Protein Coding	Anxiety Disorder; Mental Depression; Memory Loss; Memory Impairment	4	2.58	0.00%
32.	MIR22	MicroRNA 22	RNA Gene	Anxiety Disorder; Memory Loss; Memory Impairment	3	2.77	0.00%
33.	PDGFRB	Platelet Derived Growth Factor Receptor Beta	Protein Coding	Anxiety Disease; Memory Loss; Memory Impairment	3	2.68	62.04%
34.	CSF1R	Colony Stimulating Factor 1 Receptor	Protein Coding	Memory Loss; Memory Impairment	2	2.91	39.96%
35.	SQSTM1	Sequestosome 1	Protein Coding	Anxiety Disease; Memory Loss; Memory Impairment	3	2.62	40.03%
36.	ABCA7	ATP Binding Cassette Subfamily A Member 7	Protein Coding	Memory Loss; Memory Impairment	2	2.84	2.00%
37.	GRIA1	Glutamate Ionotropic Receptor AMPA Type	Protein	Anxiety Disorder; Mental Depression;	4	2.42	61.34%

No.	Symbol	Description	Category	Matched phenotypes	Matched phenotypes count	-LOG10(P)	Average disease-causing likelihood
		Subunit 1	Coding	Memory Loss; Memory Impairment			
38.	MIR34A	MicroRNA 34a	RNA Gene	Anxiety Disorder; Memory Loss; Memory Impairment	3	2.59	0.00%
39.	HLA-DQB1	Major Histocompatibility Complex; Class II; DQ Beta 1	Protein Coding	Anxiety Disease; Memory Loss; Memory Impairment	3	2.56	0.14%
40.	ACHE	Acetylcholinesterase (Cartwright Blood Group)	Protein Coding	Anxiety Disorder; Memory Loss; Memory Impairment	3	2.55	43.95%
41.	MYORG	Myogenesis Regulating Glycosidase (Putative)	Protein Coding	Memory Loss; Memory Impairment	2	2.79	0.00%
42.	NGF	Nerve Growth Factor	Protein Coding	Anxiety Disorder; Mental Depression; Memory Loss; Memory Impairment	4	2.37	35.16%
43.	AARS2	Alanyl-TRNA Synthetase 2; Mitochondrial	Protein Coding	Memory Loss; Memory Impairment	2	2.74	23.96%
44.	DNMT1	DNA Methyltransferase 1	Protein Coding	Memory Loss; Memory Impairment	2	2.73	73.22%
45.	PVALB	Parvalbumin	Protein Coding	Anxiety Disorder; Mental Depression; Memory Loss; Memory Impairment	4	2.36	51.42%
46.	CCK	Cholecystokinin	Protein Coding	Anxiety Disorder; Mental Depression; Memory Loss	3	2.48	62.52%
47.	TARDBP	TAR DNA Binding Protein	Protein Coding	Anxiety Disease; Mental Depression; Memory Loss; Memory Impairment	4	2.35	86.35%
48.	MDD1	Major Depressive Disorder	Genetic Locus	Anxiety Disorder; Mental Depression	2	2.67	0.00%
49.	SLC25A13	Solute Carrier Family 25 Member 13	Protein Coding	Memory Loss; Memory Impairment	2	2.64	78.03%
50.	OSTF1	Osteoclast Stimulating Factor 1	Protein Coding	Anxiety Disorder; Mental Depression; Memory Loss	3	2.41	68.60%

No.	Symbol	Description	Category	Matched phenotypes	Matched phenotypes count	-LOG10(P)	Average disease-causing likelihood
51.	ALB	Albumin	Protein Coding	Anxiety Disorder; Mental Depression; Memory Loss; Memory Impairment	4	2.30	82.33%
52.	MIR132	MicroRNA 132	RNA Gene	Mental Depression; Memory Loss; Memory Impairment	3	2.38	0.00%
53.	GAD2	Glutamate Decarboxylase 2	Protein Coding	Anxiety Disorder; Mental Depression; Memory Loss	3	2.38	66.16%
54.	CCR1	C-C Motif Chemokine Receptor 1	Protein Coding	Memory Loss; Memory Impairment	2	2.56	61.45%
55.	CYLD	CYLD Lysine 63 Deubiquitinase	Protein Coding	Memory Loss; Memory Impairment	2	2.54	88.26%
56.	IL23R	Interleukin 23 Receptor	Protein Coding	Memory Loss; Memory Impairment	2	2.53	38.44%
57.	C4A	Complement C4A (Rodgers Blood Group)	Protein Coding	Memory Loss; Memory Impairment	2	2.53	0.00%
58.	EIF2B3	Eukaryotic Translation Initiation Factor 2B Subunit Gamma	Protein Coding	Memory Loss; Memory Impairment	2	2.52	49.31%
59.	FRAXA	Fragile Site; Folic Acid Type; Rare; Fra(X) (Q27.3) A	Biological Region	Anxiety Disorder; Memory Loss; Memory Impairment	3	2.31	0.00%
60.	MAOB	Monoamine Oxidase B	Protein Coding	Anxiety Disorder; Mental Depression; Memory Loss; Memory Impairment	4	2.24	84.51%
61.	TAC1	Tachykinin Precursor 1	Protein Coding	Anxiety Disorder; Mental Depression; Memory Loss; Memory Impairment	4	2.23	77.26%
62.	STUB1	STIP1 Homology And U-Box Containing Protein 1	Protein Coding	Anxiety Disease; Memory Loss; Memory Impairment	3	2.31	72.64%
63.	VAMP1	Vesicle Associated Membrane Protein 1	Protein Coding	Memory Loss; Memory Impairment	2	2.49	68.90%
64.	JPH3	Junctophilin 3	Protein	Anxiety Disease; Memory Loss;	3	2.30	39.71%

No.	Symbol	Description	Category	Matched phenotypes	Matched phenotypes count	-LOG10(P)	Average disease-causing likelihood
			Coding	Memory Impairment			
65.	IL12A	Interleukin 12A	Protein Coding	Memory Loss; Memory Impairment	2	2.47	67.00%
66.	STAT4	Signal Transducer and Activator of Transcription 4	Protein Coding	Memory Loss; Memory Impairment	2	2.47	73.39%
67.	MEFV	MEFV Innate Immunity Regulator; Pyrin	Protein Coding	Memory Loss; Memory Impairment	2	2.47	10.89%
68.	XPR1	Xenotropic And Polytropic Retrovirus Receptor 1	Protein Coding	Memory Loss; Memory Impairment	2	2.43	88.78%
69.	JAM2	Junctional Adhesion Molecule 2	Protein Coding	Memory Loss; Memory Impairment	2	2.42	35.69%
70.	MSH2	MutS Homolog 2	Protein Coding	Anxiety Disease; Memory Loss; Memory Impairment	3	2.29	65.16%
71.	NIPA1	NIPA Magnesium Transporter 1	Protein Coding	Memory Loss; Memory Impairment	2	2.40	60.19%
72.	CNR1	Cannabinoid Receptor 1	Protein Coding	Anxiety Disorder; Memory Loss; Memory Impairment	3	2.29	87.47%
73.	TIA1	TIA1 Cytotoxic Granule Associated RNA Binding Protein	Protein Coding	Memory Loss; Memory Impairment	2	2.39	65.55%
74.	ATXN3	Ataxin 3	Protein Coding	Memory Loss; Memory Impairment	2	2.39	31.68%
75.	HFE	Homeostatic Iron Regulator	Protein Coding	Anxiety Disorder; Memory Loss; Memory Impairment	3	2.26	22.09%
76.	FUS	FUS RNA Binding Protein	Protein Coding	Anxiety Disease; Mental Depression; Memory Loss; Memory Impairment	4	2.17	75.66%
77.	ACE	Angiotensin I Converting Enzyme	Protein Coding	Anxiety Disorder; Memory Loss; Memory Impairment	3	2.25	15.82%

No.	Symbol	Description	Category	Matched phenotypes	Matched phenotypes count	-LOG10(P)	Average disease-causing likelihood
78.	DRD1	Dopamine Receptor D1	Protein Coding	Anxiety Disorder; Mental Depression; Memory Loss; Memory Impairment	4	2.15	73.93%
79.	DRD3	Dopamine Receptor D3	Protein Coding	Anxiety Disorder; Mental Depression; Memory Loss	3	2.24	60.23%
80.	TNFRSF1A	TNF Receptor Superfamily Member 1A	Protein Coding	Memory Loss; Memory Impairment	2	2.36	55.74%
81.	CCKBR	Cholecystokinin B Receptor	Protein Coding	Anxiety Disorder; Memory Loss; Memory Impairment	3	2.23	36.96%
82.	AD10	Alzheimer Disease-10	Genetic Locus	Memory Loss; Memory Impairment	2	2.35	0.00%
83.	SLC20A2	Solute Carrier Family 20 Member 2	Protein Coding	Memory Loss; Memory Impairment	2	2.35	69.55%
84.	SMARCE1	SWI/SNF Related; Matrix Associated; Actin Dependent Regulator of Chromatin; Subfamily E; Member 1	Protein Coding	Memory Loss; Memory Impairment	2	2.35	82.41%
85.	EIF2B2	Eukaryotic Translation Initiation Factor 2B Subunit Beta	Protein Coding	Memory Loss; Memory Impairment	2	2.32	73.46%
86.	SORL1	Sortilin Related Receptor 1	Protein Coding	Memory Loss; Memory Impairment	2	2.32	46.42%
87.	OPRM1	Opioid Receptor Mu 1	Protein Coding	Anxiety Disorder; Mental Depression; Memory Loss	3	2.22	7.81%
88.	KRAS	KRAS Proto-Oncogene; GTPase	Protein Coding	Anxiety Disease; Memory Loss; Memory Impairment	3	2.21	79.60%
89.	GAL	Galanin And GMAP Prepropeptide	Protein Coding	Anxiety Disorder; Mental Depression; Memory Loss; Memory Impairment	4	2.08	44.58%
90.	PTEN	Phosphatase And Tensin Homolog	Protein Coding	Memory Loss; Memory Impairment	2	2.30	78.93%

No.	Symbol	Description	Category	Matched phenotypes	Matched phenotypes count	-LOG10(P)	Average disease-causing likelihood
91.	FAN1	FANCD2 And FANCI Associated Nuclease 1	Protein Coding	Anxiety Disease; Memory Loss; Memory Impairment	3	2.19	28.45%
92.	BMPR1A	Bone Morphogenetic Protein Receptor Type 1A	Protein Coding	Anxiety Disease; Memory Loss; Memory Impairment	3	2.19	44.71%
93.	MT-TL1	Mitochondrially Encoded TRNA-Leu (UUA/G) 1	RNA Gene	Anxiety Disease; Memory Loss; Memory Impairment	3	2.19	0.00%
94.	POLG	DNA Polymerase Gamma; Catalytic Subunit	Protein Coding	Anxiety Disease; Memory Loss; Memory Impairment	3	2.19	56.04%
95.	CCR6	C-C Motif Chemokine Receptor 6	Protein Coding	Mental Depression; Memory Loss; Memory Impairment	3	2.16	81.89%
96.	CD4	CD4 Molecule	Protein Coding	Anxiety Disorder; Mental Depression; Memory Loss; Memory Impairment	4	2.03	34.31%
97.	AFG3L2	AFG3 Like Matrix AAA Peptidase Subunit 2	Protein Coding	Memory Loss; Memory Impairment	2	2.28	86.29%
98.	ATXN1	Ataxin 1	Protein Coding	Memory Loss; Memory Impairment	2	2.27	66.56%
99.	NOD2	Nucleotide Binding Oligomerization Domain Containing 2	Protein Coding	Memory Loss; Memory Impairment	2	2.27	18.32%
100	PSTPIP1	Proline-Serine-Threonine Phosphatase Interacting Protein 1	Protein Coding	Memory Loss; Memory Impairment	2	2.27	24.66%
101	CP	Ceruloplasmin	Protein Coding	Memory Loss; Memory Impairment	2	2.25	20.55%
102	PPIG	Peptidylprolyl Isomerase G	Protein Coding	Anxiety Disorder; Mental Depression; Memory Loss; Memory Impairment	4	2.01	47.60%
103	MT-ND4	Mitochondrially Encoded NADH: Ubiquinone Oxidoreductase Core Subunit 4	Protein Coding	Anxiety Disease; Memory Loss; Memory Impairment	3	2.12	0.00%
104	EPCAM	Epithelial Cell Adhesion Molecule	Protein	Anxiety Disease; Memory Loss;	3	2.12	11.96%

No.	Symbol	Description	Category	Matched phenotypes	Matched phenotypes count	-LOG10(P)	Average disease-causing likelihood
			Coding	Memory Impairment			
105	TGFBR2	Transforming Growth Factor Beta Receptor 2	Protein Coding	Anxiety Disease; Memory Loss; Memory Impairment	3	2.12	61.24%
106	MT-CO3	Mitochondrially Encoded Cytochrome C Oxidase III	Protein Coding	Anxiety Disease; Memory Loss; Memory Impairment	3	2.12	0.00%
107	MLH3	MutL Homolog 3	Protein Coding	Anxiety Disease; Memory Loss; Memory Impairment	3	2.12	10.60%
108	PAND1	Panic Disorder 1	Genetic Locus	Anxiety Disorder	1	2.50	0.00%
109	HTR1D	5-Hydroxytryptamine Receptor 1D	Protein Coding	Anxiety Disorder; Mental Depression	2	2.24	43.78%
110	ATN1	Atrophin 1	Protein Coding	Memory Loss; Memory Impairment	2	2.23	59.89%
111	SOD1	Superoxide Dismutase 1	Protein Coding	Anxiety Disease; Memory Loss; Memory Impairment	3	2.08	77.48%
112	HTR7	5-Hydroxytryptamine Receptor 7	Protein Coding	Anxiety Disorder; Mental Depression	2	2.23	82.77%
113	NOS1	Nitric Oxide Synthase 1	Protein Coding	Anxiety Disorder; Mental Depression; Memory Loss; Memory Impairment	4	1.99	50.65%
114	GRIK2	Glutamate Ionotropic Receptor Kainate Type Subunit 2	Protein Coding	Anxiety Disorder; Anxiety Disease; Mental Depression; Memory Impairment	4	1.98	75.34%
115	SPG11	SPG11 Vesicle Trafficking Associated; Spatacsin	Protein Coding	Memory Loss; Memory Impairment	2	2.20	49.61%
116	HTRA1	HtrA Serine Peptidase 1	Protein Coding	Memory Loss; Memory Impairment	2	2.19	63.08%
117	TOMM40	Translocase Of Outer Mitochondrial Membrane 40	Protein Coding	Memory Loss; Memory Impairment	2	2.19	72.15%

No.	Symbol	Description	Category	Matched phenotypes	Matched phenotypes count	-LOG10(P)	Average disease-causing likelihood
118	ACTB	Actin Beta	Protein Coding	Anxiety Disorder; Mental Depression; Memory Loss; Memory Impairment	4	1.96	93.46%
119	SCN1A	Sodium Voltage-Gated Channel Alpha Subunit 1	Protein Coding	Anxiety Disease; Memory Loss; Memory Impairment	3	2.01	75.96%
120	CACNA1C	Calcium Voltage-Gated Channel Subunit Alpha1 C	Protein Coding	Mental Depression; Memory Loss; Memory Impairment	3	2.01	70.84%
121	IL4	Interleukin 4	Protein Coding	Anxiety Disorder; Mental Depression; Memory Loss; Memory Impairment	4	1.95	68.48%
122	ANKLE2	Ankyrin Repeat and LEM Domain Containing 2	Protein Coding	Memory Loss; Memory Impairment	2	2.15	33.55%
123	PLAU	Plasminogen Activator; Urokinase	Protein Coding	Memory Loss; Memory Impairment	2	2.14	32.24%
124	NEFL	Neurofilament Light Chain	Protein Coding	Memory Loss; Memory Impairment	2	2.13	0.00%
125	UBQLN2	Ubiquilin 2	Protein Coding	Anxiety Disease; Mental Depression; Memory Loss; Memory Impairment	4	1.94	89.57%
126	CA4	Carbonic Anhydrase 4	Protein Coding	Anxiety Disorder; Mental Depression; Memory Loss; Memory Impairment	4	1.93	44.21%
127	NIPA2	NIPA Magnesium Transporter 2	Protein Coding	Memory Loss; Memory Impairment	2	2.13	67.44%
128	MOG	Myelin Oligodendrocyte Glycoprotein	Protein Coding	Anxiety Disorder; Memory Loss; Memory Impairment	3	1.98	31.84%
129	PSAP	Prosaposin	Protein Coding	Memory Loss; Memory Impairment	2	2.13	69.37%
130	PAH	Phenylalanine Hydroxylase	Protein Coding	Memory Loss; Memory Impairment	2	2.09	48.92%
131	CACNA1H	Calcium Voltage-Gated Channel Subunit	Protein	Anxiety Disease; Memory Loss;	3	1.98	40.67%

No.	Symbol	Description	Category	Matched phenotypes	Matched phenotypes count	-LOG10(P)	Average disease-causing likelihood
		Alpha1 H	Coding	Memory Impairment			
132	TACR1	Tachykinin Receptor 1	Protein Coding	Anxiety Disorder; Mental Depression; Memory Loss	3	1.97	79.39%
133	HM13	Histocompatibility Minor 13	Protein Coding	Anxiety Disorder; Memory Loss	2	2.07	78.83%
134	AMY1A	Amylase Alpha 1A	Protein Coding	Anxiety Disorder; Mental Depression	2	2.07	0.00%
135	LEP	Leptin	Protein Coding	Anxiety Disorder; Mental Depression; Memory Impairment	3	1.96	59.73%
136	PRKACA	Protein Kinase CAMP-Activated Catalytic Subunit Alpha	Protein Coding	Memory Loss; Memory Impairment	2	2.06	88.75%
137	PICALM	Phosphatidylinositol Binding Clathrin Assembly Protein	Protein Coding	Anxiety Disorder; Memory Loss; Memory Impairment	3	1.95	79.10%
138	SETD2	SET Domain Containing 2; Histone Lysine Methyltransferase	Protein Coding	Anxiety Disorder; Anxiety Disease; Memory Impairment	3	1.95	51.01%
139	UGT1A1	UDP Glucuronosyltransferase Family 1 Member A1	Protein Coding	Memory Loss; Memory Impairment	2	2.02	63.13%
140	USP8	Ubiquitin Specific Peptidase 8	Protein Coding	Memory Loss; Memory Impairment	2	2.02	31.98%
141	MYD88	MYD88 Innate Immune Signal Transduction Adaptor	Protein Coding	Memory Loss; Memory Impairment	2	2.02	75.67%
142	SIM1	SIM BHLH Transcription Factor 1	Protein Coding	Memory Loss; Memory Impairment	2	2.02	51.36%
143	SNCB	Synuclein Beta	Protein Coding	Memory Loss; Memory Impairment	2	2.01	76.69%
144	CRHBP	Corticotropin Releasing Hormone Binding Protein	Protein Coding	Anxiety Disorder; Mental Depression; Memory Impairment	3	1.93	53.95%

No.	Symbol	Description	Category	Matched phenotypes	Matched phenotypes count	-LOG10(P)	Average disease-causing likelihood
145	MIRLET7D	MicroRNA Let-7d	RNA Gene	Mental Depression; Memory Loss	2	2.00	0.00%
146	MIR142	MicroRNA 142	RNA Gene	Mental Depression; Memory Loss	2	2.00	0.00%
147	SLC18A2	Solute Carrier Family 18 Member A2	Protein Coding	Anxiety Disorder; Mental Depression; Memory Loss; Memory Impairment	4	1.89	78.94%
148	DNMT3A	DNA Methyltransferase 3 Alpha	Protein Coding	Anxiety Disorder; Anxiety Disease; Memory Loss; Memory Impairment	4	1.87	86.72%
149	GRM3	Glutamate Metabotropic Receptor 3	Protein Coding	Anxiety Disorder; Mental Depression; Memory Impairment	3	1.92	80.24%
150	S100B	S100 Calcium Binding Protein B	Protein Coding	Anxiety Disorder; Memory Loss; Memory Impairment	3	1.92	72.81%
151	AARS1	Alanyl-TRNA Synthetase 1	Protein Coding	Memory Loss; Memory Impairment	2	2.00	58.77%
152	GFAP	Glial Fibrillary Acidic Protein	Protein Coding	Memory Loss; Memory Impairment	2	2.00	40.30%
153	DTNBP1	Dystrobrevin Binding Protein 1	Protein Coding	Anxiety Disorder; Mental Depression; Memory Impairment	3	1.91	47.57%
154	HTR4	5-Hydroxytryptamine Receptor 4	Protein Coding	Anxiety Disorder; Mental Depression; Memory Loss	3	1.91	61.76%
155	MIR155	MicroRNA 155	RNA Gene	Mental Depression; Memory Impairment	2	1.99	0.00%
156	ASS1	Argininosuccinate Synthase 1	Protein Coding	Memory Loss; Memory Impairment	2	1.98	63.54%
157	XK	X-Linked Kx Blood Group Antigen; Kell and VPS13A Binding Protein	Protein Coding	Anxiety Disorder; Anxiety Disease; Memory Impairment	3	1.90	85.78%
158	CHAT	Choline O-Acetyltransferase	Protein Coding	Memory Loss; Memory Impairment	2	1.96	7.28%
159	HCRTR1	Hypocretin Receptor 1	Protein Coding	Anxiety Disorder; Mental Depression	2	1.96	21.33%

No.	Symbol	Description	Category	Matched phenotypes	Matched phenotypes count	-LOG10(P)	Average disease-causing likelihood
160	AD6	Alzheimer Disease 6	Genetic Locus	Memory Loss	1	2.16	0.00%
161	TP53	Tumor Protein P53	Protein Coding	Anxiety Disorder; Anxiety Disease; Memory Loss; Memory Impairment	4	1.83	72.85%
162	AMY1B	Amylase Alpha 1B	Protein Coding	Anxiety Disorder; Mental Depression	2	1.94	0.00%
163	MME	Membrane Metalloendopeptidase	Protein Coding	Memory Loss; Memory Impairment	2	1.93	69.79%
164	CXCL8	C-X-C Motif Chemokine Ligand 8	Protein Coding	Anxiety Disorder; Memory Loss; Memory Impairment	3	1.85	70.13%
165	ATP13A2	ATPase Cation Transporting 13A2	Protein Coding	Mental Depression; Memory Loss; Memory Impairment	3	1.85	67.39%
166	ITGB2	Integrin Subunit Beta 2	Protein Coding	Anxiety Disorder; Memory Loss; Memory Impairment	3	1.85	71.45%
167	MIR21	MicroRNA 21	RNA Gene	Memory Loss; Memory Impairment	2	1.91	0.00%
168	SYP	Synaptophysin	Protein Coding	Memory Loss; Memory Impairment	2	1.91	66.45%
169	CHRNB2	Cholinergic Receptor Nicotinic Beta 2 Subunit	Protein Coding	Anxiety Disease; Memory Loss; Memory Impairment	3	1.84	84.82%
170	LRP8	LDL Receptor Related Protein 8	Protein Coding	Anxiety Disorder; Mental Depression; Memory Loss	3	1.84	50.12%
171	AD5	Alzheimer Disease 5	Genetic Locus	Memory Loss	1	2.06	0.00%
172	AD8	Alzheimer Disease 8	Genetic Locus	Memory Loss	1	2.06	0.00%
173	MIR298	MicroRNA 298	RNA Gene	Memory Loss	1	2.06	0.00%
174	MIR29B1	MicroRNA 29b-1	RNA Gene	Memory Loss	1	2.06	0.00%

No.	Symbol	Description	Category	Matched phenotypes	Matched phenotypes count	-LOG10(P)	Average disease-causing likelihood
175	AD11	Alzheimer Disease-11	Genetic Locus	Memory Loss	1	2.06	0.00%
176	AD13	Alzheimer Disease-13	Genetic Locus	Memory Loss	1	2.06	0.00%
177	MIR107	MicroRNA 107	RNA Gene	Memory Loss	1	2.06	0.00%
178	MIR106B	MicroRNA 106b	RNA Gene	Memory Loss	1	2.06	0.00%
179	MIR146A	MicroRNA 146a	RNA Gene	Memory Loss	1	2.06	0.00%
180	AD16	Alzheimer Disease 16	Genetic Locus	Memory Loss	1	2.06	0.00%
181	AIF1	Allograft Inflammatory Factor 1	Protein Coding	Memory Loss; Memory Impairment	2	1.91	22.37%
182	PINK1	PTEN Induced Kinase 1	Protein Coding	Anxiety Disease; Memory Loss; Memory Impairment	3	1.83	16.06%
183	C19orf12	Chromosome 19 Open Reading Frame 12	Protein Coding	Memory Loss; Memory Impairment	2	1.89	47.95%
184	ATP1A2	ATPase Na+/K+ Transporting Subunit Alpha 2	Protein Coding	Memory Loss; Memory Impairment	2	1.89	85.48%
185	ASMT	Acetyl serotonin O-Methyltransferase	Protein Coding	Anxiety Disorder; Mental Depression; Memory Impairment	3	1.83	10.57%
186	NR3C2	Nuclear Receptor Subfamily 3 Group C Member 2	Protein Coding	Anxiety Disorder; Mental Depression; Memory Impairment	3	1.83	63.80%
187	BCHE	Butyrylcholinesterase	Protein Coding	Memory Loss; Memory Impairment	2	1.89	17.99%
188	ENO2	Enolase 2	Protein Coding	Memory Loss; Memory Impairment	2	1.89	86.64%
189	CREBBP	CREB Binding Protein	Protein Coding	Anxiety Disorder; Memory Loss; Memory Impairment	3	1.77	69.94%

No.	Symbol	Description	Category	Matched phenotypes	Matched phenotypes count	-LOG10(P)	Average disease-causing likelihood
190	AIP	Aryl Hydrocarbon Receptor Interacting Protein	Protein Coding	Anxiety Disease; Memory Impairment	2	1.87	51.53%
191	CST3	Cystatin C	Protein Coding	Memory Loss; Memory Impairment	2	1.86	0.00%
192	RNASEH1	Ribonuclease H1	Protein Coding	Memory Loss; Memory Impairment	2	1.86	82.83%
193	YY1	YY1 Transcription Factor	Protein Coding	Anxiety Disease; Memory Loss; Memory Impairment	3	1.76	55.78%
194	NOS2	Nitric Oxide Synthase 2	Protein Coding	Memory Loss; Memory Impairment	2	1.86	46.59%
195	RHO	Rhodopsin	Protein Coding	Anxiety Disorder; Mental Depression; Memory Impairment	3	1.76	73.02%
196	RORA	RAR Related Orphan Receptor A	Protein Coding	Anxiety Disorder; Mental Depression; Memory Impairment	3	1.76	86.61%
197	SPTLC1	Serine Palmitoyl transferase Long Chain Base Subunit 1	Protein Coding	Memory Impairment	1	1.99	65.31%
198	DLGAP2	DLG Associated Protein 2	Protein Coding	Anxiety Disorder	1	1.99	0.00%
199	PSENEN	Presenilin Enhancer; Gamma-Secretase Subunit	Protein Coding	Memory Loss; Memory Impairment	2	1.85	68.50%
200	CDK5	Cyclin Dependent Kinase 5	Protein Coding	"Memory Loss", "Memory Impairment"	2	1.85	83.41%

Table S6. Myocardial infarction related pathogenic genes retrieved for the CLAE compounds.

**Compounds**	**Common name**
Isooientin	AKR1B1, CA7, CA12, TNF, IL2, AKT1, ABCB1, ALOX5, NOX4, CDK5R1 CDK5, XDH, MAOA, FLT3, CA2, CCNB3 CDK1 CCNB1 CCNB2, GLO1, APP, SYK, GSK3B, PARP1, TTR, MMP9, MMP2, CA4, MMP12, CD38, CYP1B1, ABCG2, AKR1B10, TNKS2, TNKS, ADORA1, ARG1, HIF1A, ABCC1, RPS6KA3, EGFR, PTPRS, SLC29A1, PRKCG, PRKCB, PRKCZ, SQLE, PLG, MAPT, KDM4E, GPR35, AVPR2, TOP2A, IGF1R, CYP19A1, INSR, F2, PIM1, AURKB, DRD4, ACHE, MYLK, MPO, PIK3R1, DAPK1, PYGL, CA1, SRC, PTK2, HSD17B2, KDR, MMP13, MMP3, CA3, ALOX15, PLK1, CA6, CDK1, PIK3CG, PKN1, CA14, CA9, CSNK2A1, ALOX12, MET, NEK2, CXCR1, CAMK2B, ALK, NEK6, PLA2G1B, CA5A, AXL, APEX1, NUAK1, AKR1C2, AKR1C1, AKR1C3, AKR1C4, CA13, ADORA2A, AKR1A1, PRKACA, BCL2L1.
Isosilybin A	CA2, CA7, CA1, CA3, CA6, CA12, CA4, CA13, CA5B, ABCB1, CA5A, MMP13, MMP12, CYP1B1, TAS2R31, KLK1, KLK2, CYP19A1, KIT, MMP2, MMP14, PTGS1, MAPT, TERT, PGD, ST3GAL3, FUT7, FUT4, STAT1, SQLE, HIF1A, GABRA1 GABRB2 GABRG2, BCL2, ESR2, ABCG2, ABCC1, SHBG, CBR1, MAOB, KDR, RXRA, KCNH2, MET, ADORA3, MT-ND4, ADORA1, SRC, CA9, FGFR1, APP, BACE1, FFAR1, DNMT1, MMP8, ESR1, PLA2G1B, CES1, CES2, HSD17B1, PTGS2, MAPK14, MAP2K1, CYP2C19, MMP9, DYRK1A, NADK, GRM2, PGF, VEGFA, PRKACA, F2, IRAK4, AURKA, ADORA2A, EIF4A1, PIK3C2A, PIK3C3, TYMS, SYK, MTOR, MAP3K9, PARP1, PIK3CD, PIK3CB, PIK3CG, PIK3CA, FGR, PIK3C2B, TNKS2, TNKS, ADAM10, CHRM4, CHRM5, CHRM2, CHRM1, CHRM3, POLB, ADORA2B, AKR1C3, PLA2G2A.
Vanillic acid	CA2, CA7, CA1, CA12, CA14, CA9, CA3, CA6, CA5A, CA4, TPMT, TTR, CA5B, CA13, FUT7, KDM4E, KDM4A, KDM3A, KDM6B, FTO, KDM4C, FYN, LCK, FBP1, AKR1C3, KDM2A, MMP9, MMP1, MMP2, MMP8, SQLE, POLA1, POLB, SERPINE1, TUBB1, NGFR, KMO, TYMS, PTPRB, LDHA, LDHB, LAP3, GPR17, MIF, IDO1, CTBP2, IGF1R, ALK, SRD5A2, PLA2G4B, ELANE, ERN1, LIG1, ACHE, FUCA1, GRM2, DPP4, SLC13A5, OGA, MAOA, MAOB, HSP90AA1, COMT, AKR1B1, BCL2L1, ESR2, CNR2, SLC29A1, CPA1, ALB, DTYMK, CASP3, SHBG, PIN1, RXRB, RXRG, RXRA, APEX1, CDA, TYR, ACE, ADA, NAALAD2, AMPD3, TRPM8, FABP4, FABP3, FABP5, MCL1, GGH, ECE1, ESR1, TBXAS1, MME, ST14, PLAU, HMGCR, DBF4 CDC7, SLC16A1, AURKB.

Table S7. Anxiety, depression and impaired memory related pathogenic genes retrieved for CLAE compounds.

Compound name	Genes
14,15-Dehydro-16-epi-vincamine	CYP17A1, SLC6A2, KCNA5, PARP1, MDM2, CTSS, CAPN2, CAPN1, CCNE2 CDK2 CCNE1, CCNB3 CDK1 CCNB1 CCNB2, ALOX5, CTSK, SCN9A, CYP19A1, JAK3, HSD17B2, CASP1, FNTA FNTB, ADORA1, PGGT1B, PPOX, CTSB, CASP3, CASP6, CASP7, CASP8, PTK2B, DUSP3, MCL1, TLR9, NR3C2, NR3C1, MAPK8, MMP9, MMP2, MMP8, TNNC1 TNNT2 TNNI3, TERT, STAT3, HSD11B1, AURKA, DUT, TTR, ERBB2, JAK2, GRM5, HMOX1, NTRK1, ADAMTS5, MET, PGGT1B FNTA, PER2, HCRTR2, HCRTR1, BACE1, EIF2AK1, LRRK2, CTSL, PAK4, PAK1, STK3, STK26, FAAH, AGTR1, MMP1, NPY5R, PRCP, MAPK10, HIF1A, PTGS2, MAPK14, GSK3B, SRC, SOAT1, MMP3, ADAM17, MMP7, QRFPR, CHRM4, CCNE1 CDK2, HRH2, FLT3, CHRM5, RET, CHRM2, CHRM3, PTAFR, BACE2, LCK, FGFR3, MUSK, NR4A1, AURKB, TACR1, ADORA2A, ITK, NOS1, NPY2R, KCNA3, NOS3.
Biochanin A	CYP19A1, CA7, HSD17B2, CA12, CA4, CBR1, ABCB1, HSD17B1, ESR1, ESR2, ALOX12, EGFR, ABCG2, ALDH2, TBXAS1, MAOA, MGAM, HTR2A, HTR2C, ADORA1, ADORA2A, ESRRA, ESRRB, IL2, PTGS1, SLC6A2, ALOX15, XDH, TYR, MIF, CA2, PTPRS, PPARA, TLR9, CA1, CYP1B1, PFKFB3, PON1, CA5B, MAOB, NOX4, STS, ACHE, PTPN1, HSP90AB1, CA9, CDC7, GCGR, PLAT, F10, PLAU, MCL1, GPR84, DUSP3, CDC25B, ABCC1, HSP90B1, BAD, TBXA2R, CHEK1, WEE1, ALPL, HSP90AA1, CA3, CA6, CA14, CA13, CA5A, IGFBP3, PPARG, TNNC1 TNNT2 TNNI3, SNCA, PTK6, BCL2, TLR8, SORT1, BACE1, PRMT1, IGFBP6, IGFBP4, IGFBP5, IGFBP2, IGFBP1, GRIN2B, AKR1B1, KIF11, CCND1 CDK4, PRSS1, CCR4, CHEK2, TNF, RPS6KA3, PDE10A, OPRD1, PLA2G4A, RAF1, COMT, NTRK1, PIM2, BRAF.
Ellagic Acid	GPR35, ERBB2, AKR1B1, CCND1 CDK4, PDGFRB, FLT4, IGF1R, INSR, EGFR, CA2, CDK2 CCNA1 CCNA2, AURKB, CA7, CA1, GSK3B, SRC, PTK2, KDR, PLK1, CA6, CA12, CA14, CA9, CSNK2A1, MET, CA4, PLK4, CA13, TEK, AKT1, AURKA, CA5A, BACE1, MAP3K8, BRAF, EPHB4, HSPA1A, NUAK1, SQLE, FGR, LYN, GSR, TNNC1 TNNT2 TNNI3, XDH, DAO, PTGS2, ESR1, ESR2, MAOA, HSD17B3, PTPN1, ALOX5, CBR1, CDK5R1 CDK5, CES2, CA5B, SNCA, ACHE, CYP1A2, ALDH2, GRK6, SRD5A1, CA3, KCNA3, MAOB, AKR1C3, AKR1C1, STS, PIM1, ALK, AXL, MIF, ERN1, CNR1, CNR2, NFKB1, KCNA5, APEX1, FYN, LCK, PON1, DYRK1A, BCHE, AR, ALOX15, PFKFB3, NQO1, SHBG, OPRK1, CYP19A1, PDE4D, IKBKB, NTRK2, TTR, CYP1B1, SLC6A3, SLC16A3, SIRT1, TERT, GPR55.
Enterolactone	CYP19A1, SHBG, MAP2K1, HSD17B2, NR1H3, ESR1, ESR2, PPARD, SLC6A3, SLC6A2, F2, LYPLA1, LYPLA2, ALPL, PRSS1, ALOX5, HSD17B1, HSP90AA1, CAPN1, PARP1, CDK2 CCNA1 CCNA2, MYLK, MAPK14, TGFBR1, GSTA1, NQO2, CHRNA4 CHRNB2, ADORA2A, MMP3, MMP7, HSD11B1, NOX4, ADORA2B, JUN, PSEN2 PSENEN NCSTN APH1A PSEN1 APH1B, KIT, FLT4, FLT3, PDGFRA, MAP2K2, PITRM1, BRAF, CPA1, CPA3, CPB2, ABL1, MAP2, CCKBR, GCGR, MTNR1B, DRD1, BCL2, JAK2, RET, ALB, RIPK2, FTO, SGK1, HDAC5, GSK3B, HDAC7, ROCK2, ROCK1, LIMK1, CDC42BPA, TNK2, DMPK, RAF1, PDPK1, MAPK8, CDK2, PLK1, NOS1, FGFR1, MKNK2, NOS2, PAK4,

Compound name	Genes
	CHEK1, PTGFR, LRRK2, GRK7, ERBB2, CCNE1 CDK2, ANPEP, JAK3, DYRK1A, CAMK1, RPS6KA1, CAMK2D, JAK1, PKN2, JAK3 JAK1, JAK1 JAK2, TYK2 JAK2 JAK1, TYK2 JAK1, TYK2 JAK2, PKN1, PLA2G7, TYK2, RPS6KA2.
Gancaonin E	ABCG2, ESR1, ESR2, CYP19A1, ACHE, PPARG, CES2, CA7, CA12, PLA2G1B, BCHE, HSD17B1, CA4, CYP1B1, SLC5A2, BACE1, MMP12, MMP13, CES1, RXRA, PTGS1, PTPN1, ODC1, ADORA1, ADORA3, MAOB, SHBG, CTSL, KLK2, TAS2R31, EPHB4, ABL1, EPHA2, EPHB2, EPHA5, EPHA4, EPHA8, EPHA7, EPHB3, EPHA3, EPHB1, EPHA1, RPS6KB1, AURKA, MMP3, ABCC1, KLK1, CBR1, HSD17B2, WEE1, RAF1, BCL2L1, BRAF, SERPINE1, CA6, CA13, CA5B, CA5A, PDGFRB, SRC, AKR1B10, HSP90AA1, CA2, CA1, DRD5, DRD1, DRD4, DRD3, F3, ESRRA, ESRRB, TRPM8, CAPN1, CHEK1, MET, HDAC3, GCGR, HDAC9, CCNE1 CDK2, CCNE1 CDK3, POLB, ANPEP, PDK1, NOX4, CDK1 CCNB1, MYLK, GRK2, KIT, PDE7A, CDK1, CA14, MMP8, PLA2G5, PLA2G10, HDAC7, MTOR, MMP1, ADAM17, FYN, YES1.
Isoorientin	AKR1B1, CA7, CA12, TNF, IL2, AKT1, ABCB1, ALOX5, NOX4, CDK5R1 CDK5, XDH, MAOA, FLT3, CA2, CCNB3 CDK1 CCNB1 CCNB2, GLO1, APP, SYK, GSK3B, PARP1, TTR, MMP9, MMP2, CA4, MMP12, CD38, CYP1B1, ABCG2, AKR1B10, TNKS2, TNKS, ADORA1, ARG1, HIF1A, ABCC1, RPS6KA3, EGFR, PTPRS, SLC29A1, PRKCG, PRKCB, PRKCZ, SQLE, PLG, MAPT, KDM4E, GPR35, AVPR2, TOP2A, IGF1R, CYP19A1, INSR, F2, PIM1, AURKB, DRD4, ACHE, MYLK, MPO, PIK3R1, DAPK1, PYGL, CA1, SRC, PTK2, HSD17B2, KDR, MMP13, MMP3, CA3, ALOX15, PLK1, CA6, CDK1, PIK3CG, PKN1, CA14, CA9, CSNK2A1, ALOX12, MET, NEK2, CXCR1, CAMK2B, ALK, NEK6, PLA2G1B, CA5A, AXL, APEX1, NUAK1, AKR1C2, AKR1C1, AKR1C3, AKR1C4, CA13, ADORA2A, AKR1A1, PRKACA, BCL2L1.
Isosilybin A	CA2, CA7, CA1, CA3, CA6, CA12, CA4, CA13, CA5B, ABCB1, CA5A, MMP13, MMP12, CYP1B1, TAS2R31, KLK1, KLK2, CYP19A1, KIT, MMP2, MMP14, PTGS1, MAPT, TERT, PGD, ST3GAL3, FUT7, FUT4, STAT1, SQLE, HIF1A, GABRA1 GABRB2 GABRG2, BCL2, ESR2, ABCG2, ABCC1, SHBG, CBR1, MAOB, KDR, RXRA, KCNH2, MET, ADORA3, MT-ND4, ADORA1, SRC, CA9, FGFR1, APP, BACE1, FFAR1, DNMT1, MMP8, ESR1, PLA2G1B, CES1, CES2, HSD17B1, PTGS2, MAPK14, MAP2K1, CYP2C19, MMP9, DYRK1A, NADK, GRM2, PGF, VEGFA, PRKACA, F2, IRAK4, AURKA, ADORA2A, EIF4A1, PIK3C2A, PIK3C3, TYMS, SYK, MTOR, MAP3K9, PARP1, PIK3CD, PIK3CB, PIK3CG, PIK3CA, FGR, PIK3C2B, TNKS2, TNKS, ADAM10, CHRM4, CHRM5, CHRM2, CHRM1, CHRM3, POLB, ADORA2B, AKR1C3, PLA2G2A.
Quercetin	NOX4, AVPR2, AKR1B1, XDH, MAOA, IGF1R, FLT3, CYP19A1, EGFR, F2, CA2, PIM1, ALOX5, AURKB, DRD4, ADORA1, CA7, GLO1, MPO, PIK3R1, ADORA2A, DAPK1, PYGL, CA1, GSK3B, SRC, PTK2, HSD17B2, KDR, MMP13, MMP3, CA3, ALOX15, ABCC1, PLK1, CA6, CDK1, MMP9, CA12, MMP2, PKN1, CA14, CA9, CSNK2A1, ALOX12, MET, CA4, NEK2, CXCR1, CAMK2B, ALK, AKT1, ABCB1, NEK6, PLA2G1B, CA5A, BACE1, CYP1B1, AXL, ABCG2, NUAK1, AKR1C2, AKR1C1, AKR1C3, AKR1C4, CA13, AKR1A1, GPR35, MAPT, KDM4E, TOP2A, INSR, ACHE, MYLK, SYK,

Compound name	Genes
	PIK3CG, APEX1, PTPRS, ESR2, MPG, SLC22A12, CDK5R1 CDK5, CCNB3 CDK1 CCNB1 CCNB2, ARG1, CDK6, CDK2, TYR, HSD17B1, AHR, ESRRA, APP, PARP1, TTR, MMP12, CD38, AKR1B10, TNKS2, TNKS, TOP1, TERT.
Scaposin	AKR1B1, ABCG2, OPRD1, KIT, CA2, CA4, NOS2, OPRM1, ABCC1, NOX4, PTGS2, ADORA3, XDH, PLA2G2A, ALOX5, CA12, CYP1B1, PFKFB3, CA9, MCL1, CA7, MAPT, KDM4E, TOP2A, DRD4, GLO1, MYLK, MPO, PIK3R1, DAPK1, PYGL, SYK, MMP3, CA3, CA6, CSNK2A1, CA13, PLA2G1B, CA5A, APEX1, AKR1C2, AKR1C1, AKR1C3, AKR1C4, AKR1A1, APP, GSK3B, BACE1, CDK1, PARP1, TTR, MMP12, CD38, AKR1B10, TNKS2, TNKS, TOP1, ARG1, ABCB1, ALOX15, ALOX12, PIM1, AKT1, F2, CYP1A1, CYP1A2, MMP9, MMP2, PLG, ESR2, TERT, AMY1A, HSD17B2, FLT3, TYR, AHR, ESRRA, AXL, CA1, ADORA2A, IKBKB, IGF1R, AURKB, SRC, PTK2, KDR, PLK1, PKN1, MET, NEK2, ALK, NEK6, NUAK1, ADORA1, NTRK2, CYP19A1, PIK3CG, INSR, EGFR, MPG.
Scopoletin	CA7, CA12, CA9, CA13, CA1, CA14, CA6, CA4, EGFR, CA5A, XDH, CA2, MAOA, CBR1, SRD5A1, CDK2 CCNA1 CCNA2, CA3, CCND1 CDK4, FLT4, INSR, PTK2, TEK, MAP3K8, HSPA1A, NUAK1, FGR, ESR2, AKR1C1, ALOX5, CA5B, GSK3B, DAO, KCNA3, GSR, KDR, ACHE, PTGS2, KCNA5, HSD17B3, SRC, CYP1A2, MAOB, NAT1, SQLE, PTPN1, KCNMA1, MB, BACE1, ERBB2, ESR1, PARP1, AKR1C3, MET, ALPG, PLAA, ALPL, COMT, GPR35, AURKA, CDK9 CCNT1, HMGCR, CSNK2A1, AKR1B1, PLEC, CSNK1A1, CSNK1D, AOC3, AURKB, AKT1, APEX1, CHRM1, CISD1, ALDH5A1, ABAT, BRAF, IGF1R, PIK3CG, PTPRC, GRK6, HTR2B, CXCR1, CLK1, DYRK1B, TNNC1 TNNT2 TNNI3, TAAR1, EPHB4, F2, ADRA2A, ADRA2C, ADRA2B, TERT, NISCH, CHEK1, WEE1, PLK1, PNP, PDGFRB, LYN, PLK4, CHRNA7.
Vasicine	BCHE, ACHE, HRH3, NOS1, NOS2, NOS3, JAK1, JAK2, HMGCR, JAK3, SYK, ZAP70, STAT5A, AR, PSEN2 PSENEN NCSTN APH1A PSEN1 APH1B, ADORA1, ADORA3, ADRB2, ADRB1, ADRB3, ADRA1D, ADRA1A, ADRA1B, CYP19A1, CHEK1, PYGL, NR3C1, CA7, SERPINA6, CA1, CA12, CA9, CA13, SCN9A, PGR, SHBG, SLC6A3, PRKCA, PRKDC, MAPK1, ADORA2A, TTR, PIM1, PIM3, OPRK1, MMP3, MMP9, MMP1, MMP2, MMP7, MMP8, LIMK2, CYP11B1, CYP11B2, NEK1, MAPK14, CDC25A, CA2, TYK2, CDK2 CCNA1 CCNA2, LRRK2, CHRNA3 CHRNB4, GABRB3 GABRA3 GABRG2, GABRB3 GABRG2 GABRA1, GABRB3 GABRG2 GABRA5, GABRA2 GABRB3 GABRG2, PDE7A, CDK2, ADAM17, AKR1C3, POLA1, DPP4, MAP2K1, DPP8, FAP, DPP9, TTL, IMPDH1, IMPDH2, EGFR, DRD1, GSK3B, IKBKB, OPRL1, HLCS, PARP1, MMP13, TK1, PER2, HSD11B1, PDE10A, ADAM10, ESR2, ADK, DRD3, EDNRA, KDR, HRH4, MAP3K7, COMT.

Table S8. Top 20 GO biological process of CLAE phytocompounds for myocardial infarction related target genes.

No.	Enrichment FDR	No. of genes	Pathway Genes	Fold Enrichment	Pathway	Genes
1.	4.12E-07	4	11	369.67	Cellular response to UV-A	MMP2, MMP9, MMP3, MMP1
2.	5.59E-07	6	113	53.98	Collagen metabolic process	MMP2, HIF1A, MMP9, MMP3, F2, MMP1
3.	5.59E-07	4	14	290.45	Response to UV-A	MMP2, MMP9, MMP3, MMP1
4.	3.05E-06	6	157	38.85	Response to UV	PTGS2, MMP2, MMP9, MMP3, CASP3, MMP1
5.	3.59E-06	8	490	16.60	Response to oxidative stress	MPO, PTGS2, MMP2, HIF1A, MMP9, MMP3, CASP3, CA3
6.	6.33E-06	7	339	20.99	Response to light stimulus	PTGS2, MMP2, HIF1A, MMP9, MMP3, CASP3, MMP1
7.	6.33E-06	5	94	54.07	Extracellular matrix disassembly	MMP2, MMP9, PLG, MMP3, MMP1
8.	6.33E-06	5	93	54.65	Cellular response to UV	PTGS2, MMP2, MMP9, MMP3, MMP1
9.	8.44E-06	7	375	18.98	Cellular response to chemical stress	MPO, PTGS2, MMP2, HIF1A, MMP9, MMP3, CASP3
10.	2.48E-05	3	11	277.25	Negative regulation of fibrinolysis	SERPINE1, PLG, F2
11.	2.66E-05	4	52	78.20	Embryo implantation	PTGS2, MMP2, MMP9, VEGFA
12.	2.66E-05	7	483	14.73	Response to radiation	PTGS2, MMP2, HIF1A, MMP9, MMP3, CASP3, MMP1
13.	2.66E-05	4	53	76.72	Collagen catabolic process	MMP2, MMP9, MMP3, MMP1
14.	2.66E-05	11	1817	6.15	Regulation of cell population proliferation	PTGS2, MMP2, ESR1, HIF1A, MMP9, VEGFA, FABP3, PLG, ACE, CASP3, F2

No.	Enrichment FDR	No. of genes	Pathway Genes	Fold Enrichment	Pathway	Genes
15.	2.66E-05	5	142	35.80	Cellular response to light stimulus	PTGS2, MMP2, MMP9, MMP3, MMP1
16.	2.66E-05	11	1832	6.10	Response to oxygen-containing compound	MPO, PTGS2, MMP2, ESR1, HIF1A, MMP9, SERPINE1, FABP3, MMP3, CASP3, CA3
17.	4.02E-05	3	15	203.32	Regulation of fibrinolysis	SERPINE1, PLG, F2
18.	7.44E-05	6	350	17.43	Cellular response to abiotic stimulus	PTGS2, MMP2, MMP9, MMP3, CASP3, MMP1
19.	7.44E-05	6	350	17.43	Cellular response to environmental stimulus	PTGS2, MMP2, MMP9, MMP3, CASP3, MMP1
20.	8.12E-05	3	20	152.49	Mammary gland alveolus development	ESR1, HIF1A, VEGFA

Table S9. Top 20 KEGG pathway of CLAE phytocompounds for myocardial infarction related target genes.

No.	Enrichment FDR	No. of genes	Pathway genes	Fold enrichment	Pathway	Genes
1.	6.57E-06	6	93	27.50	IL-17 signaling pathway	PTGS2, MMP9, MMP3, CASP3, MMP1, TNF
2.	1.48E-05	7	202	14.77	Proteoglycans in cancer	MMP2, ESR1, HIF1A, MMP9, VEGFA, CASP3, TNF
3.	4.91E-05	9	530	7.24	Pathways in cancer	PTGS2, MMP2, ESR1, HIF1A, MMP9, VEGFA, CASP3, F2, MMP1
4.	7.26E-05	4	41	41.58	Bladder cancer	MMP2, MMP9, VEGFA, MMP1
5.	7.37E-05	5	100	21.31	AGE-RAGE signaling pathway in diabetic complications	MMP2, SERPINE1, VEGFA, CASP3, TNF
6.	1.08E-04	5	112	19.03	TNF signaling pathway	PTGS2, MMP9, MMP3, CASP3, TNF
7.	1.06E-03	4	92	18.53	Rheumatoid arthritis	VEGFA, MMP3, MMP1, TNF
8.	1.85E-03	5	214	9.96	Lipid and atherosclerosis	MMP9, MMP3, CASP3, MMP1, TNF
9.	2.41E-03	5	232	9.19	Coronavirus disease	MMP3, ACE, F2, MMP1, TNF
10.	2.77E-03	4	129	13.22	Relaxin signaling pathway	MMP2, MMP9, VEGFA, MMP1
11.	3.26E-03	4	138	12.35	Fluid shear stress and atherosclerosis	MMP2, MMP9, VEGFA, TNF
12.	5.39E-03	4	161	10.59	MicroRNAs in cancer	PTGS2, MMP9, VEGFA, CASP3
13.	9.33E-03	3	84	15.22	Complement and coagulation cascades	SERPINE1, PLG, F2
14.	9.33E-03	4	194	8.79	Kaposi sarcoma-associated herpesvirus infection	PTGS2, HIF1A, VEGFA, CASP3
15.	1.23E-02	3	95	13.46	Endocrine resistance	MMP2, ESR1, MMP9
16.	1.31E-02	3	101	12.66	Chagas disease	SERPINE1, ACE, TNF
17.	1.31E-02	4	224	7.61	Human cytomegalovirus infection	PTGS2, VEGFA, CASP3, TNF
18.	1.53E-02	3	109	11.73	HIF-1 signaling pathway	HIF1A, SERPINE1, VEGFA
19.	1.56E-02	3	112	11.42	Serotonergic synapse	PTGS2, CASP3, CYP2C19
20.	2.69E-02	3	138	9.27	Estrogen signaling pathway	MMP2, ESR1, MMP9

Table S10. Top 20 GO biological process of CLAE phytocompounds for anxiety, depression and impaired memory related target genes.

No.	Enrichment FDR	No. of genes	Pathway Genes	Fold Enrichment	Pathway	Genes
1.	1.23E-13	10	90	74.00	Monoamine transport	MAOB, NOS1, HTR2A, SLC6A2, CNR1, SNCA, DRD3, DRD1, DRD4, SLC6A3
2.	1.89E-12	16	776	13.73	Trans-synaptic signaling	ACHE, NOS1, HTR2A, SLC6A2, OPRM1, BCHE, CNR1, HCRTR1, APP, AKT1, SNCA, DRD3, DRD1, DRD4, SLC6A3, PRKACA
3.	2.71E-12	16	815	13.08	Synaptic signaling	ACHE, NOS1, HTR2A, SLC6A2, OPRM1, BCHE, CNR1, HCRTR1, APP, AKT1, SNCA, DRD3, DRD1, DRD4, SLC6A3, PRKACA
4.	5.10E-12	8	54	98.67	Dopamine transport	HTR2A, SLC6A2, CNR1, SNCA, DRD3, DRD1, DRD4, SLC6A3
5.	6.42E-12	11	235	31.18	Regulation of neurotransmitter levels	MAOB, ACHE, NOS1, HTR2A, SLC6A2, CNR1, SNCA, DRD3, DRD1, DRD4, SLC6A3
6.	6.42E-12	6	14	285.44	Dopamine uptake involved in synaptic transmission	SLC6A2, SNCA, DRD3, DRD1, DRD4, SLC6A3
7.	6.42E-12	6	14	285.44	Catecholamine uptake involved in synaptic transmission	SLC6A2, SNCA, DRD3, DRD1, DRD4, SLC6A3
8.	9.70E-12	13	470	18.42	Cellular calcium ion homeostasis	NOS1, HTR2A, CCKBR, OPRM1, TACR1, CNR1, HCRTR1, APP, SNCA, DRD3, DRD1, DRD4, PRKACA
9.	9.70E-12	15	767	13.03	Chemical synaptic transmission	ACHE, HTR2A, SLC6A2, OPRM1, BCHE, CNR1, HCRTR1, APP, AKT1, SNCA, DRD3, DRD1, DRD4, SLC6A3, PRKACA
10.	9.70E-12	15	767	13.03	Anterograde trans-synaptic signaling	ACHE, HTR2A, SLC6A2, OPRM1, BCHE, CNR1, HCRTR1, APP, AKT1, SNCA, DRD3, DRD1, DRD4, SLC6A3, PRKACA
11.	1.22E-11	13	482	17.96	Calcium ion homeostasis	NOS1, HTR2A, CCKBR, OPRM1, TACR1, CNR1, HCRTR1, APP, SNCA, DRD3, DRD1, DRD4, PRKACA
12.	1.50E-11	7	37	126.01	Neurotransmitter reuptake	NOS1, SLC6A2, SNCA, DRD3, DRD1, DRD4, SLC6A3
13.	2.23E-11	13	512	16.91	Cellular divalent inorganic cation homeostasis	NOS1, HTR2A, CCKBR, OPRM1, TACR1, CNR1, HCRTR1, APP, SNCA, DRD3, DRD1, DRD4, PRKACA

No.	Enrichment FDR	No. of genes	Pathway Genes	Fold Enrichment	Pathway	Genes
14.	3.07E-11	8	78	68.31	Catecholamine transport	HTR2A, SLC6A2, CNR1, SNCA, DRD3, DRD1, DRD4, SLC6A3
15.	3.07E-11	13	531	16.31	Divalent inorganic cation homeostasis	NOS1, HTR2A, CCKBR, OPRM1, TACR1, CNR1, HCRTR1, APP, SNCA, DRD3, DRD1, DRD4, PRKACA
16.	3.60E-11	6	20	199.81	Dopamine uptake	SLC6A2, SNCA, DRD3, DRD1, DRD4, SLC6A3
17.	4.75E-11	6	21	190.30	Catecholamine uptake	SLC6A2, SNCA, DRD3, DRD1, DRD4, SLC6A3
18.	9.56E-11	7	50	93.24	Neurotransmitter uptake	NOS1, SLC6A2, SNCA, DRD3, DRD1, DRD4, SLC6A3
19.	1.53E-10	13	616	14.06	Cellular metal ion homeostasis	NOS1, HTR2A, CCKBR, OPRM1, TACR1, CNR1, HCRTR1, APP, SNCA, DRD3, DRD1, DRD4, PRKACA
20.	1.53E-10	15	979	10.20	Cellular homeostasis	NOS2, NOS1, HTR2A, SLC6A2, CCKBR, OPRM1, TACR1, CNR1, HCRTR1, APP, SNCA, DRD3, DRD1, DRD4, PRKACA

Table S11. Top 20 KEGG pathway of CLAE phytocompounds for anxiety, depression and impaired memory related target genes.

No.	Enrichment FDR	No. of genes	Pathway Genes	Fold Enrichment	Pathway	Genes
1.	4.64E-05	7	132	15.34	Dopaminergic synapse	MAOB, AKT1, DRD3, DRD1, DRD4, SLC6A3, PRKACA
2.	9.86E-05	8	240	9.64	Calcium signaling pathway	NOS2, NOS1, HTR2A, CCKBR, PDGFRB, TACR1, DRD1, PRKACA
3.	1.05E-04	9	350	7.44	Neuroactive ligand-receptor interaction	HTR2A, CCKBR, OPRM1, TACR1, CNR1, HCRTR1, DRD3, DRD1, DRD4
4.	1.09E-03	4	49	23.61	Cocaine addiction	MAOB, DRD1, SLC6A3, PRKACA
5.	3.39E-03	4	69	16.77	Amphetamine addiction	MAOB, DRD1, SLC6A3, PRKACA
6.	6.25E-03	4	88	13.15	Gap junction	HTR2A, PDGFRB, DRD1, PRKACA
7.	6.25E-03	7	383	5.29	Alzheimer disease	NOS2, NOS1, APP, AKT1, SNCA, MT-ND4, TNF
8.	6.25E-03	6	266	6.52	Parkinson disease	MAOB, SNCA, DRD1, MT-ND4, SLC6A3, PRKACA
9.	1.22E-02	4	112	10.33	Serotonergic synapse	MAOB, HTR2A, APP, PRKACA
10.	1.38E-02	3	51	17.01	Arginine and proline metabolism	NOS2, MAOB, NOS1
11.	1.56E-02	4	129	8.97	Relaxin signaling pathway	NOS2, NOS1, AKT1, PRKACA
12.	1.56E-02	7	475	4.26	Pathways of neurodegeneration	NOS2, NOS1, APP, SNCA, MT-ND4, TNF, SLC6A3
13.	1.85E-02	4	138	8.38	Apelin signaling pathway	NOS2, NOS1, AKT1, PRKACA
14.	3.65E-02	2	22	26.29	Arginine biosynthesis	NOS2, NOS1
15.	4.45E-02	3	92	9.43	Salivary secretion	NOS1, AMY1A, PRKACA
16.	4.45E-02	3	91	9.53	Morphine addiction	OPRM1, DRD1, PRKACA
17.	4.45E-02	4	187	6.19	Alcoholism	MAOB, DRD1, SLC6A3, PRKACA
18.	4.82E-02	3	101	8.59	Chagas disease	NOS2, AKT1, TNF
19.	4.82E-02	3	102	8.51	Amoebiasis	NOS2, TNF, PRKACA

No.	Enrichment FDR	No. of genes	Pathway Genes	Fold Enrichment	Pathway	Genes
20.	4.82E-02	4	202	5.73	Proteoglycans in cancer	PLAU, AKT1, TNF, PRKACA

Table S12. Network analysis of myocardial infarction related target genes interaction with CLAE compounds.

No.	Name	Betweenness centrality	Closeness centrality	Degree	Topological coefficient	Type
1.	FABP3	0	0.259259259	1	0	Gene
2.	ACE	0	0.259259259	1	0	Gene
3.	CASP3	0	0.259259259	1	0	Gene
4.	ALB	0	0.259259259	1	0	Gene
5.	SERPINE1	0	0.355932203	1	0	Gene
6.	MMP1	0	0.355932203	1	0	Gene
7.	VEGFA	0	0.355932203	1	0	Gene
8.	CYP2C19	0	0.355932203	1	0	Gene
9.	PTGS2	0	0.355932203	1	0	Gene
10.	Vanillic acid	0.352381	0.344262295	5	0.2	Compound
11.	ESR1	0.380952	0.428571429	2	0.5	Gene
12.	CA3	0.057143	0.428571429	2	0.642857	Gene
13.	MMP3	0	0.287671233	1	0	Gene
14.	MPO	0	0.287671233	1	0	Gene
15.	F2	0.057143	0.428571429	2	0.642857	Gene
16.	PLG	0	0.287671233	1	0	Gene
17.	HIF1A	0.057143	0.428571429	2	0.642857	Gene
18.	MMP2	0.057143	0.428571429	2	0.642857	Gene
19.	Isosilybin A	0.738095	0.538461538	11	0.272727	Compound
20.	MMP9	0.057143	0.428571429	2	0.642857	Gene
21.	Isoorientin	0.37619	0.396226415	9	0.555556	Compound
22.	TNF	0	0.287671233	1	0	Gene

Table S13. Network analysis of myocardial infarction related target genes interaction with CLAE compounds and GO biological process (BP).

No.	Name	Betweenness centrality	Closeness centrality	Degree	Topological coefficient	Type
1.	Vanillic acid	0.077978	0.513514	9	0.368687	Compound
2.	Isosilybin A	0.048745	0.5	8	0.426136	Compound
3.	Isooientin	0.054599	0.5	8	0.443182	Compound
4.	Mammary gland alveolus development	0.003216	0.351852	3	0.484848	Pathway
5.	Cellular response to environmental stimulus	0.005936	0.44186	6	0.754386	Pathway
6.	Cellular response to abiotic stimulus	0.005936	0.44186	6	0.754386	Pathway
7.	Regulation of fibrinolysis	0.003329	0.333333	3	0.5	Pathway
8.	Response to oxygen-containing compound	0.094609	0.542857	11	0.409091	Pathway
9.	Cellular response to light stimulus	0.003356	0.431818	5	0.810526	Pathway
10.	ACE	0.000802	0.383838	2	0.692308	Gene
11.	FABP3	0.002216	0.408602	3	0.622222	Gene
12.	ESR1	0.017878	0.426966	5	0.493333	Gene
13.	Regulation of cell population proliferation	0.122507	0.542857	11	0.359504	Pathway
14.	Collagen catabolic process	0.001631	0.422222	4	0.855263	Pathway
15.	Response to radiation	0.012079	0.463415	7	0.678571	Pathway
16.	VEGFA	0.010355	0.383838	4	0.5	Gene
17.	Embryo implantation	0.010501	0.431818	4	0.6625	Pathway
18.	Negative regulation of fibrinolysis	0.003329	0.333333	3	0.5	Pathway
19.	SERPINE1	0.027789	0.408602	4	0.392857	Gene
20.	Cellular response to chemical stress	0.017393	0.463415	7	0.614286	Pathway
21.	Cellular response to UV	0.003356	0.431818	5	0.810526	Pathway
22.	PLG	0.034321	0.426966	5	0.333333	Gene
23.	Extracellular matrix disassembly	0.018788	0.452381	5	0.657143	Pathway
24.	Response to light stimulus	0.012079	0.463415	7	0.678571	Pathway
25.	CA3	0.008135	0.426966	5	0.52	Gene
26.	Response to oxidative stress	0.025699	0.475	8	0.5625	Pathway

No.	Name	Betweenness centrality	Closeness centrality	Degree	Topological coefficient	Type
27.	MPO	0.003436	0.391753	4	0.625	Gene
28.	CASP3	0.03214	0.481013	10	0.453333	Gene
29.	Response to UV	0.005936	0.44186	6	0.754386	Pathway
30.	PTGS2	0.05708	0.520548	13	0.4	Gene
31.	Response to UV-A	0.001631	0.422222	4	0.855263	Pathway
32.	F2	0.047877	0.436782	6	0.366667	Gene
33.	HIF1A	0.057391	0.481013	10	0.44	Gene
34.	Collagen metabolic process	0.023342	0.475	6	0.598485	Pathway
35.	MMP1	0.046216	0.493506	13	0.360947	Gene
36.	MMP3	0.076103	0.535211	16	0.399038	Gene
37.	MMP9	0.16253	0.644068	20	0.37	Gene
38.	Cellular response to UV-A	0.001631	0.422222	4	0.855263	Pathway
39.	MMP2	0.16253	0.644068	20	0.37	Gene

Table S14. Network analysis of myocardial infarction related target genes interaction with CLAE compounds and KEGG pathways.

No.	Name	Betweenness centrality	Closeness centrality	Degree	Topological coefficient	Type
1.	Vanillic acid	0.078589	0.493333	7	0.331169	Compounds
2.	Isooientin	0.086283	0.480519	7	0.326531	Compounds
3.	Isosilybin A	0.089004	0.493333	8	0.333333	Compounds
4.	Estrogen signaling pathway	0.002494	0.373737	3	0.666667	Pathway
5.	CYP2C19	0.003567	0.345794	2	0.5625	Gene
6.	Serotonergic synapse	0.010853	0.345794	3	0.545455	Pathway
7.	HIF-1 signaling pathway	0.010598	0.381443	3	0.444444	Pathway
8.	Human cytomegalovirus infection	0.01099	0.425287	4	0.5	Pathway
9.	Chagas disease	0.011439	0.359223	3	0.410256	Pathway
10.	Endocrine resistance	0.002494	0.373737	3	0.666667	Pathway
11.	Kaposi sarcoma-associated herpesvirus infection	0.008886	0.406593	4	0.485294	Pathway
12.	PLG	0.005037	0.33945	2	0.571429	Gene
13.	Complement and coagulation cascades	0.01149	0.333333	3	0.375	Pathway
14.	MicroRNAs in cancer	0.008884	0.425287	4	0.539474	Pathway
15.	Fluid shear stress and atherosclerosis	0.009854	0.435294	4	0.55	Pathway
16.	Relaxin signaling pathway	0.008129	0.425287	4	0.539474	Pathway
17.	ACE	0.008095	0.366337	3	0.444444	Gene
18.	Coronavirus disease	0.027828	0.406593	5	0.35	Pathway
19.	Lipid and atherosclerosis	0.017424	0.445783	5	0.45	Pathway
20.	Rheumatoid arthritis	0.012353	0.41573	4	0.458333	Pathway
21.	TNF signaling pathway	0.020224	0.445783	5	0.45	Pathway
22.	SERPINE1	0.039859	0.397849	5	0.290909	Gene
23.	AGE-RAGE signaling pathway in diabetic complications	0.031677	0.468354	5	0.409091	Pathway
24.	Bladder cancer	0.008129	0.425287	4	0.539474	Pathway

No.	Name	Betweenness centrality	Closeness centrality	Degree	Topological coefficient	Type
25.	F2	0.047056	0.425287	5	0.385714	Gene
26.	Pathways in cancer	0.076829	0.506849	9	0.380952	Pathway
27.	VEGFA	0.111641	0.480519	12	0.333333	Gene
28.	HIF1A	0.032107	0.425287	6	0.410256	Gene
29.	ESR1	0.023215	0.41573	6	0.430556	Gene
30.	Proteoglycans in cancer	0.039391	0.480519	7	0.435374	Pathway
31.	MMP2	0.088208	0.493333	11	0.324675	Gene
32.	TNF	0.112643	0.480519	11	0.307692	Gene
33.	MMP1	0.04458	0.435294	8	0.375	Gene
34.	CASP3	0.098944	0.480519	11	0.335664	Gene
35.	MMP3	0.021558	0.406593	6	0.393939	Gene
36.	MMP9	0.152187	0.536232	14	0.316327	Gene
37.	IL-17 signaling pathway	0.028769	0.45679	6	0.433333	Pathway
38.	PTGS2	0.046139	0.425287	8	0.397727	Gene

Table S15. Network analysis of anxiety, depression and impaired memory related target genes interaction with CLAE constituents.

No.	Name	Betweenness centrality	Closeness centrality	Degree	Topological coefficient	Type
1.	AMY1A	0	0.277027027	1	0	Gene
2.	OPRM1	0	0.277027027	1	0	Gene
3.	MT-ND4	0	0.259493671	1	0	Gene
4.	PRKACA	0.004378121	0.284722222	2	0.611111111	Gene
5.	APP	0.028845002	0.3203125	4	0.4375	Gene
6.	DRD3	0.003698078	0.315384615	2	0.65	Gene
7.	DRD4	0.018468104	0.325396825	4	0.410714286	Gene
8.	DNMT1	0.048780488	0.262820513	2	0.5	Gene
9.	NOS2	0.062510063	0.347457627	3	0.4	Gene
10.	ALB	0	0.262820513	1	0	Gene
11.	DRD1	0.029694403	0.336065574	3	0.487179487	Gene
12.	CCKBR	0	0.262820513	1	0	Gene
13.	SLC6A3	0.039730535	0.347457627	3	0.444444444	Gene
14.	BCHE	0.012298072	0.336065574	3	0.538461538	Gene
15.	CNR1	0	0.284722222	1	0	Gene
16.	AKT1	0.029763503	0.336065574	5	0.373333333	Gene
17.	PDGFRB	0.00455241	0.305970149	3	0.575757576	Gene
18.	TNF	0.008852841	0.325396825	2	0.583333333	Gene
19.	SNCA	0.007601722	0.330645161	2	0.615384615	Gene
20.	PLAU	0	0.301470588	1	0	Gene
21.	ACHE	0.118744555	0.41	7	0.307142857	Gene
22.	MAOB	0.092031434	0.401960784	5	0.32	Gene
23.	HTR2C	0	0.301470588	1	0	Gene
24.	HTR2A	0	0.301470588	1	0	Gene
25.	Scopoletin	0.019701531	0.347457627	5	0.575	Compound
26.	Scaposin	0.146525192	0.37962963	7	0.301587302	Compound
27.	Quercetin	0.021167229	0.347457627	5	0.575	Compound
28.	Isosilybin A	0.16325299	0.347457627	6	0.333333333	Compound
29.	Isoorientin	0.073714884	0.359649123	7	0.446428571	Compound
30.	Gancaonin E	0.110287341	0.386792453	8	0.375	Compound
31.	Ellagic Acid	0.149186773	0.394230769	9	0.345679012	Compound
32.	CA4	0.201238297	0.455555556	8	0.260869565	Gene
33.	Vasicine	0.111117197	0.37962963	7	0.26984127	Compound
34.	NOS1	0.065515371	0.310606061	3	0.444444444	Gene
35.	TACR1	0	0.227777778	1	0	Gene
36.	HCRTR1	0	0.227777778	1	0	Gene
37.	NR3C2	0	0.227777778	1	0	Gene
38.	Enterolactone	0.148789729	0.353448276	7	0.238095238	Compounds
39.	Biochanin A	0.28927684	0.427083333	9	0.233333333	Compounds
40.	14,15-Dehydro-16-epi-vincamine	0.146248588	0.292857143	5	0.266666667	Compounds

No.	Name	Betweenness centrality	Closeness centrality	Degree	Topological coefficient	Type
41.	SLC6A2	0.152565294	0.359649123	3	0.352941176	Gene

Table S16. Network analysis of anxiety, depression and impaired memory related target genes interaction with CLAE compounds and GO biological process (BP).

No.	Name	Betweenness centrality	Closeness centrality	Degree	Topological coefficient	Type
1.	MAOB	0.018413	0.393701	7	0.387755102	Gene
2.	Monoamine transport	0.018139	0.5	10	0.56	Pathway
3.	NOS1	0.036584	0.49505	15	0.522807018	Gene
4.	HTR2A	0.025688	0.49505	15	0.592982456	Gene
5.	SLC6A2	0.056211	0.526316	18	0.435672515	Gene
6.	CNR1	0.025396	0.49505	15	0.6	Gene
7.	SNCA	0.06932	0.574713	22	0.495215311	Gene
8.	DRD3	0.063845	0.574713	22	0.497607656	Gene
9.	DRD1	0.075672	0.588235	23	0.487414188	Gene
10.	DRD4	0.108737	0.60241	24	0.467105263	Gene
11.	SLC6A3	0.040689	0.505051	17	0.45751634	Gene
12.	ACHE	0.04241	0.45045	12	0.480392157	Gene
13.	Trans-synaptic signaling	0.045514	0.568182	16	0.48125	Pathway
14.	OPRM1	0.012112	0.45045	11	0.686868687	Gene
15.	BCHE	0.006052	0.413223	7	0.630252101	Gene
16.	HCRTR1	0.014307	0.45045	11	0.681818182	Gene
17.	APP	0.043354	0.485437	14	0.545112782	Gene
18.	AKT1	0.025143	0.42735	9	0.503267974	Gene
19.	PRKACA	0.026287	0.46729	12	0.605263158	Gene
20.	Synaptic signaling	0.045514	0.568182	16	0.48125	Pathway
21.	Dopamine transport	0.0046	0.462963	8	0.660714286	Pathway
22.	Regulation of neurotransmitter levels	0.024173	0.510204	11	0.542424242	Pathway
23.	Dopamine uptake involved in synaptic transmission	0.002126	0.446429	6	0.714285714	Pathway
24.	Catecholamine uptake involved in synaptic transmission	0.002126	0.446429	6	0.714285714	Pathway

No.	Name	Betweenness centrality	Closeness centrality	Degree	Topological coefficient	Type
25.	Cellular calcium ion homeostasis	0.023578	0.520833	13	0.49071618	Pathway
26.	CCKBR	0.004423	0.393701	7	0.752380952	Gene
27.	TACR1	0.005002	0.387597	7	0.785714286	Gene
28.	Chemical synaptic transmission	0.039752	0.555556	15	0.482222222	Pathway
29.	Anterograde trans-synaptic signaling	0.039752	0.555556	15	0.482222222	Pathway
30.	Calcium ion homeostasis	0.023578	0.520833	13	0.49071618	Pathway
31.	Neurotransmitter reuptake	0.003586	0.454545	7	0.683673469	Pathway
32.	Cellular divalent inorganic cation homeostasis	0.023578	0.520833	13	0.49071618	Pathway
33.	Catecholamine transport	0.0046	0.462963	8	0.660714286	Pathway
34.	Divalent inorganic cation homeostasis	0.023578	0.520833	13	0.49071618	Pathway
35.	Dopamine uptake	0.002126	0.446429	6	0.714285714	Pathway
36.	Catecholamine uptake	0.002126	0.446429	6	0.714285714	Pathway
37.	Neurotransmitter uptake	0.003586	0.454545	7	0.683673469	Pathway
38.	Cellular metal ion homeostasis	0.023578	0.520833	13	0.49071618	Pathway
39.	NOS2	0.004028	0.4	4	0.402777778	Gene
40.	Cellular homeostasis	0.048098	0.543478	15	0.471264368	Pathway
41.	Biochanin A	0.00638	0.446429	5	0.475862069	Compound
42.	Ellagic Acid	0.01214	0.462963	7	0.403940887	Compound
43.	Gancaonin E	0.009884	0.454545	6	0.511494253	Compound
44.	Isosilybin A	0.003372	0.367647	3	0.526315789	Compound
45.	Scopoletin	0.001767	0.333333	3	0.595238095	Compound
46.	14,15-Dehydro-16-epi-vincamine	0.002287	0.396825	4	0.510869565	Compound
47.	Enterolactone	0.009966	0.431034	6	0.5	Compound
48.	Vasicine	0.014557	0.462963	7	0.458128079	Compound
49.	Isoorientin	0.004311	0.438596	5	0.471428571	Compound
50.	Quercetin	0.002749	0.431034	4	0.491071429	Compound
51.	Scaposin	0.008066	0.438596	5	0.407142857	Compound

Table S17. Network analysis of anxiety, depression, and impaired memory related target genes interaction with CLAE constituents and KEGG signaling pathways.

No.	Name	Betweenness centrality	Closeness centrality	Degree	Topological coefficient	Type
1.	MAOB	0.089248	0.429752	12	0.223958	Gene
2.	Dopaminergic synapse	0.045737	0.448276	7	0.321429	Pathway
3.	AKT1	0.095618	0.429752	11	0.219251	Gene
4.	DRD3	0.00971	0.374101	4	0.366667	Gene
5.	DRD1	0.092533	0.436975	12	0.254902	Gene
6.	DRD4	0.037368	0.396947	6	0.284314	Gene
7.	SLC6A3	0.040099	0.403101	9	0.288889	Gene
8.	PRKACA	0.18071	0.485981	16	0.180921	Gene
9.	NOS2	0.101069	0.444444	12	0.208333	Gene
10.	Calcium signaling pathway	0.084369	0.464286	8	0.25431	Pathway
11.	NOS1	0.101235	0.429752	11	0.213904	Gene
12.	HTR2A	0.029342	0.390977	5	0.294118	Gene
13.	CCKBR	0.005295	0.358621	3	0.487179	Gene
14.	PDGFRB	0.017379	0.374101	5	0.3	Gene
15.	TACR1	0.009231	0.353741	3	0.472222	Gene
16.	Neuroactive ligand-receptor interaction	0.089018	0.393939	9	0.191358	Pathway
17.	OPRM1	0.009559	0.358621	3	0.384615	Gene
18.	CNR1	0.004796	0.339869	2	0.5	Gene
19.	HCRTR1	0.003698	0.315152	2	0.555556	Gene
20.	Cocaine addiction	0.007502	0.4	4	0.46875	Pathway
21.	Amphetamine addiction	0.007502	0.4	4	0.46875	Pathway
22.	Gap junction	0.011559	0.38806	4	0.386364	Pathway
23.	Alzheimer disease	0.042049	0.412698	7	0.310559	Pathway
24.	APP	0.032558	0.379562	7	0.318681	Gene
25.	SNCA	0.016356	0.374101	5	0.371429	Gene

26.	MT-ND4	0.005854	0.348993	4	0.5	Gene
27.	TNF	0.029695	0.374101	7	0.321429	Gene
28.	Parkinson disease	0.02986	0.419355	6	0.346667	Pathway
29.	Serotonergic synapse	0.014749	0.40625	4	0.36	Pathway
30.	Arginine and proline metabolism	0.010019	0.393939	3	0.444444	Pathway
31.	Relaxin signaling pathway	0.013673	0.419355	4	0.425926	Pathway
32.	Pathways of neurodegeneration	0.041866	0.42623	7	0.274286	Pathway
33.	Apelin signaling pathway	0.013673	0.419355	4	0.425926	Pathway
34.	Arginine biosynthesis	0.000638	0.325	2	0.807692	Pathway
35.	Salivary secretion	0.019438	0.382353	3	0.376812	Pathway
36.	AMY1A	0.00227	0.322981	2	0.5	Gene
37.	Morphine addiction	0.010639	0.371429	3	0.466667	Pathway
38.	Alcoholism	0.007502	0.4	4	0.46875	Pathway
39.	Chagas disease	0.005121	0.356164	3	0.5	Pathway
40.	Amoebiasis	0.007253	0.382353	3	0.444444	Pathway
41.	PLAU	0.001159	0.307692	2	0.583333	Gene
42.	Proteoglycans in cancer	0.026405	0.38806	4	0.347826	Pathway
43.	Biochanin A	0.032857	0.376812	5	0.26	Compound
44.	Ellagic Acid	0.041912	0.419355	6	0.253333	Compound
45.	Gancaonin E	0.017952	0.382353	5	0.34	Compound
46.	Isosilybin A	0.013203	0.393939	4	0.364583	Compound
47.	Scopoletin	0.00664	0.371429	3	0.396825	Compound
48.	Isoorientin	0.021882	0.412698	5	0.336	Compound
49.	Quercetin	0.004495	0.346667	3	0.466667	Compound
50.	Scaposin	0.05207	0.40625	6	0.253623	Compound
51.	Vasicine	0.022334	0.393939	5	0.373913	Compound
52.	Enterolactone	0.021728	0.393939	5	0.365217	Compound
53.	14,15-Dehydro-16-epi-vincamine	0.014666	0.325	3	0.393939	Compound
